# A randomized, double-blind, placebo-controlled, parallel group study on the effects of a cathepsin S inhibitor in primary Sjögren’s syndrome

**DOI:** 10.1093/rheumatology/kead092

**Published:** 2023-03-02

**Authors:** Darren Bentley, Benjamin A Fisher, Francesca Barone, Fabrice A Kolb, Gemma Attley

**Affiliations:** Certara UK Ltd, Sheffield, UK; Institute of Inflammation and Ageing, College of Medical and Dental Sciences, University of Birmingham, Birmingham, UK; National Institute of Health Research (NIHR) Birmingham Biomedical Research Centre and Department of Rheumatology, University Hospitals Birmingham NHS Foundation Trust, Birmingham, UK; Candel Therapeutics, Needham, MA, USA; Galapagos GmbH, Basel, Switzerland; Roche Pharma Research and Early Development, Little Falls, NJ, USA

**Keywords:** primary SS, cathepsin S, ESSDAI, phase II, placebo

## Abstract

**Objectives:**

Primary SS (pSS) is a chronic autoimmune disorder characterized by mucosal dryness and systemic symptoms. We tested the effects of inhibition of cathepsin S using the potent and selective inhibitor RO5459072 on disease activity and symptoms of pSS.

**Methods:**

This was a randomized, double-blind, placebo-controlled, parallel-group, Phase IIA study to investigate the effects of RO5459072 (100 mg twice daily; 200 mg per day). Seventy-five patients with pSS were randomized 1:1 to receive either RO5459072 or placebo for 12 weeks. The primary outcome was the proportion of patients with a ≥3 point reduction from baseline in EULAR SS Disease Activity Index (ESSDAI) score. We also investigated the effects of RO5459072 on quality of life, exocrine gland function, biomarkers related to SS, and safety and tolerability.

**Results:**

The proportion of patients showing an improvement in ESSDAI score was not significantly different between the RO5459072 and placebo arms. No clinically meaningful treatment effects were observed in favour of RO5459072 for all secondary outcomes. Analysis of soluble biomarkers indicated target engagement between RO5459072 and cathepsin S. There were modest decreases in the number of circulating B cells and T cells in the RO5459072 group, although these did not reach significance. RO5459072 was safe and well-tolerated.

**Conclusions:**

There was no clinically relevant improvement in ESSDAI score (primary endpoint), and no apparent benefit in favour of RO5459072 in any of the secondary clinical endpoints. Further work is needed in order to understand the mechanisms of MHC-II-mediated immune stimulation in pSS.

**Trial registration:**

ClinicalTrials.gov; NCT02701985.

Rheumatology key messages

- The cathepsin S inhibitor RO5459072 did not confer a clinically meaningful benefit in patients with primary SS.
- Cathepsin S may not be a relevant therapeutic target for treatment of primary SS.

## Introduction

Primary pSS is a chronic autoimmune disease that is characterized by sicca and mainly involves the exocrine glands, affecting between 0.1% and 0.4% of the population depending on the country [1–3]. Lymphocytic infiltration of the exocrine glands and epithelia is the underlying driver of disease pathology, ultimately resulting in secretory gland dysfunction and dryness of the mucosal surfaces, such as the eyes and mouth [4]. Patients also exhibit a wide array of systemic symptoms, such as fatigue, neuropathy, muscle and joint pain, and some experience life-threatening immunological manifestations such as encephalitis, vasculitis and lymphoma [5].

The pathogenesis of pSS is not fully understood, but multiple factors appear to be involved. Among the key steps are the entry of follicular B cells and T cells into exocrine glands, increased cytokine production, B cell hyperactivity and autoantibody production [6, 7]. Other immune cells and chemokines have also been shown to participate in the destruction of glandular architecture, underscoring the complexity of this systemic disorder. To date, there is no disease-modifying therapy. Treatment revolves around supportive therapy (i.e. exercise for treatment of fatigue [8]), symptomatic management [9] and various systemic therapies [10].

Cathepsin S is a cysteine protease that is expressed in antigen-presenting cells including macrophages, B cells and dendritic cells [11]. One of its main roles is the cleavage of the MHC-II-bound invariant chain pro-peptide Lip10 (p10), during the processing of MHC-II antigen–peptide complexes [12, 13]. There is evidence that elevated cathepsin S activity may lead to increased MHC-II expression and autoimmunity, implying that inhibition of cathepsin S may be a potential approach in attenuating auto-antigenic T cell responses [14]. In keeping with this, the tears of patients with primary and secondary SS contain higher levels of cathepsin S activity compared with healthy individuals or those with other autoimmune diseases [15, 16]. Inhibition of cathepsin S in murine models of SS reduced inflammation of the lacrimal and salivary glands and improved the secretion of tears and saliva [14, 17].

RO5459072 is a covalent, reversible and selective inhibitor of cathepsin S developed for the treatment of autoimmune conditions, including SS [18]. Inhibition of cathepsin S by RO5459072 is expected to reduce MHC-II-mediated antigen presentation, and attenuate the activation of CD4+ T cells. This would result in suppression of T cell–dependent autoantibody production and neutralization of the tissue damage caused by activated macrophages and neutrophils [19]. Results from pre-clinical studies suggest that inhibition of cathepsin S by RO5459072 could result in decreased MHC-II maturation and reduced antigen presentation, representing a novel treatment strategy for pSS [12, 19, 20].

The objective of this study was to investigate the effects of RO5459072 on disease activity and symptoms in patients with pSS. The safety and tolerability of RO5459072 was also assessed in our study population of patients with moderate to severe pSS.

## Methods

The protocol for this study and CONSORT checklist are available as Supplementary Material, available at *Rheumatology* online.

### Study design

This was a randomized, double-blind, placebo-controlled, parallel-group Phase IIA trial designed to evaluate the effects of RO5459072 treatment on disease activity and symptoms in adult patients with moderate to severe pSS. Patients were randomized 1:1 to receive either oral RO5459072 [100 mg twice daily (BID), total of 200 mg daily] or matching placebo, and were treated for a maximum of 12 weeks. Patients were recruited between July 2016 and March 2017 in the USA (9 centres), France (3 centres), UK (4 centres), Germany (1 center), Poland (3 centres) and Portugal (3 centres).

### Study participants

**Figure 1.**
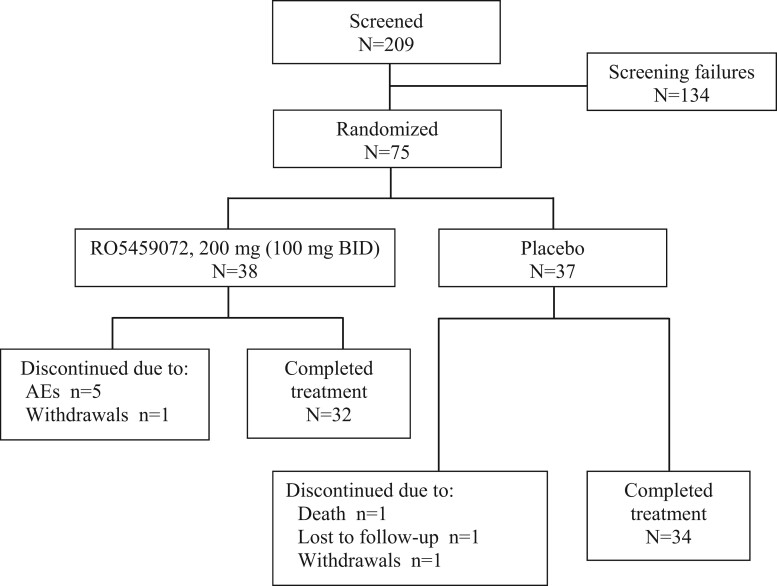
Flow chart of inclusion in the study

We enrolled male and female adults (18–75 years) with moderate to severe pSS, previously diagnosed according to the revised American-European Consensus Group criteria. The study flow diagram is given in Fig. 1.

The severity of disease activity was defined according to the EULAR SS Disease Activity Index (ESSDAI) score of ≥5. Patients also had to have a SS Patient Reported Index (ESSPRI) score of ≥5, and elevated serum titers of anti-SSA and/or anti-SSB antibodies at screening. We excluded patients with secondary SS, and those with severe complications of SS (including vasculitis with renal, neurologic or cardiac involvement; interstitial lung disease and severe myositis). Prohibited concomitant medications included: CS therapy exceeding the equivalent of 7.5 mg prednisone per day, anti-CD20 therapy (e.g. rituximab) or other B cell–depleting therapy within 6 months of screening, immunosuppressant therapy and CYC.

### Study treatment and randomization

RO5459072 (two hard gelatin capsules containing 50 mg drug substance) or matching placebo was taken orally twice daily with food (morning and evening) for 12 weeks. The investigators, patients and study site staff were blinded to the treatment allocation.

### Study assessments and endpoints

**Figure 2.**
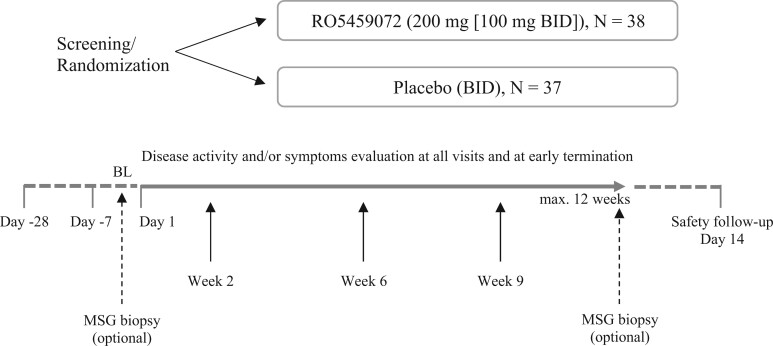
Overview of study design. BL: baseline; MSG: minor salivary gland

Assessments of SS disease activity, symptoms and quality of life were carried out before (at baseline), during and upon completion of the study treatment phase, at early termination visits, and at the safety and follow-up visit (Fig. 2). Patients also underwent assessments of exocrine gland function and provided samples for measurement of biomarkers related to pSS or the mechanism of action of RO5459072. Safety and tolerability were assessed throughout the study. Consenting patients also underwent minor salivary gland biopsies in order to assess histological changes (Fig. 2). Details are given in the study protocol (Supplementary Material available at *Rheumatology* online).

The primary endpoint was the proportion of patients who had a ≥3 point reduction from baseline in the ESSDAI score after 12 weeks of treatment [21, 22]. Secondary endpoints were the mean differences from baseline at week 12 for ESSDAI, ESSPRI, the Short Form-36 Health Survey (SF-36) mental and physical components, unstimulated tear production and mechanically stimulated salivary flow [23–26]. Stimulated salivary flow was measured using mechanical stimulation. Unstimulated tear production rate was measured using the Schirmer method. Salivary and tear samples were also used for biomarker measurements. Soluble biomarkers [cathepsin S mass, cystatin C, B cell activating factor (BAFF), 4-β-hydroxycholesterol, desmosine, isodesmosine], as well as the changes from baseline in mean numbers of B cells, T cells and monocytes, were estimated.

Safety assessments consisted of monitoring and recording adverse events (AEs), including serious AEs (SAEs) and non-serious AEs of special interest, monitoring of vital signs (systolic blood pressure, diastolic blood pressure, pulse rate), safety ECGs and laboratory safety tests.

### Statistical analysis

The sample size was calculated to detect a difference in ESSDAI response rates between treatment arms in the range of 25–33% (two-sided χ^2^ test assuming α = 0.05 and placebo rate ≤25%). Seventy patients (35 per group) would provide ∼70% power to detect a difference of 3 points between treatment arms in the change from baseline in ESSDAI score (*t*-test assuming two-sided α = 0.05 and s.d. of 5.0). All efficacy outcomes were analysed according to the modified intent-to-treat (mITT) principle. The mITT population consisted of all patients who were randomized and who had received at least one dose of study drug.

The proportion of patients who had ≥3-point reduction from baseline in ESSDAI score after 12 weeks of treatment were compared between the two treatment arms using a Pearson χ^2^ test (two-sided *P*-values, α = 0.05). Secondary endpoints that were binary were analysed with similar methods used for analysing the primary endpoint. Continuous secondary endpoints were analysed using a Mixed Model for Repeated Measures (MMRM) approach.

All patients who received at least one dose of the study medication were included in the safety analysis population.

### Ethics

The study was conducted according to the principles of the Declaration of Helsinki and Good Clinical Practice. The study protocol was approved by the Ethics Committees of each centre; all patients provided written informed consent prior to enrolment.

## Results

### Patient characteristics

Seventy-five patients were enrolled in 23 study centers in the USA, UK, Poland, Portugal, France and Germany. Thirty-eight patients were randomized to RO5459072 treatment and 37 to placebo (Fig. 1). Sixty-six (88.0%) completed treatment; nine patients discontinued from the study (Fig. 1). All 75 randomized patients were included in the analyses of efficacy and safety.

Baseline characteristics of the patients were similar in both arms (Table 1). The median age was 54 years; the majority were female [68 (90.7%)]. The proportion of males was slightly higher in the RO5459072 arm [6 (15.8%) compared with 1 (2.7%) in the placebo arm]. Positive ANA autoantibodies were detected in 62/75 participants (82.7%). ANA-negative patients had a statistically significant lower baseline IgG adjusted mean concentration than ANA-positive patients (–4.42 g/l; *P* = 0.0140). Forty-three patients (57%) were positive for both anti-SSA/Ro and anti-SSB/La antibodies. The adjusted mean concentration (g/l) of IgG at baseline between anti-SSB-negative (13.61) and -positive patients (15.78) did not reach statistical significance (*P* = 0.1251). The domains contributing to the total ESSDAI scores are shown in Supplementary Figs S1 and S2, available at *Rheumatology* online. As in other recent trials, the domains making the largest contribution were the articular, biological, glandular, constitutional and lymphadenopathy domains. The most frequently used concomitant medications (≥20% of patients in each study arm) included analgesics [RO5459072: 11/38 (28.9%); placebo: 2/37 (5.4%)] and antihistamines [RO5459072: 13/38 (34.2%); placebo: 4/37 (10.8%)]. The number of patients with one or more previous medical condition was 35/37 (94.6%) in the placebo arm and 36/38 (94.7%) in the RO5459072 arm.

**Table 1.** Baseline characteristics of the study population.

	Placebo (*N* = 37)	RO5459072 (*N* = 38)	Both (*N* = 75)
Age (years)			
Mean (s.d.)	52.3 (11.8)	52.1 (13.2)	52.2 (12.5)
Median	53.0	54.5	54.0
Min–max	30–73	21–75	21–75
Age group (years), *n* (%)			
<65	32 (86.5)	32 (84.2)	64 (85.3)
≥65	5 (13.5)	6 (15.8)	11 (14.7)
Gender, *n* (%)			
Male	1 (2.7)	6 (15.8)	7 (9.3)
Female	36 (97.3)	32 (84.2)	68 (90.7)
Race, *n* (%)			
Asian	1 (2.7)	1 (2.6)	2 (2.7)
Black or African American	3 (8.1)	2 (5.3)	5 (6.7)
Caucasian	33 (89.2)	35 (92.1)	68 (90.7)
Ethnicity, *n* (%)			
Hispanic or Latino	2 (5.4)	0	2 (2.7)
Non-Hispanic and non-Latino	35 (94.6)	38 (100.0)	73 (97.3)
Country, *n* (%)			
Germany	7 (18.9)	6 (15.8)	13 (17.3)
France	4 (10.8)	3 (7.9)	7 (9.3)
UK	1 (2.7)	7 (18.4)	8 (10.7)
Poland	10 (27.0)	9 (23.7)	19 (25.3)
Portugal	3 (8.1)	3 (7.9)	6 (8.0)
US	12 (32.4)	10 (26.3)	22 (29.3)
Weight (kg)			
Mean (s.d.)	71.98 (24.06)	72.46 (17.60)	72.23 (20.89)
Median	66.40	66.75	66.50
Min–max	50.0–172.0	48.0–111.0	48.0–172.0
Baseline ESSDAI scores			
Mean (SD)	11.27 (5.71)	11.79 (4.69)	–
Baseline ESSPRI scores			
Mean (SD)	7.34 (1.19)	6.98 (0.98)	–
Anti-SSA status, *n* (%)			
Positive	37 (100)	38 (100)	75 (100)
Negative	0	0	0
Anti-SSB status, *n* (%)			
Positive	22 (59.5)	18 (47.4)	40 (53.3)
Negative	15 (40.5)	20 (52.6)	35 (46.7)
RF status, *n* (%)			
Positive	18 (48.6)	17 (44.7)	35 (46.7)
Negative	19 (51.4)	21 (55.3)	40 (53.3)
IgG			
Mean (s.d.)	15.74 (6.77)	13.59 (4.92)	14.65 (5.96)
IgM			
Mean (s.d.)	1.24 (0.82)	1.26 (0.75)	1.25 (0.78)
Baseline mechanically stimulated salivary flow rate, ml/min			
Mean (s.d.)	0.45 (0.29)	0.55 (0.58)	0.50 (0.46)
Baseline Schirmer’s test for tear flow, mm/5 min			
Mean (s.d.)	7.53 (9.66)	7.84 (10.15)	7.69 (9.84)
Received MTX treatment (at least once)	6 (16.2)	10 (26.3)	16 (21.3)
Received HCQ medications (at least once)	16 (43.2)	24 (63.2)	40 (53.3)
Received CS medications (at least once)	19 (51.4)	17 (44.7)	36 (48.0)

### Efficacy

The proportion of patients showing an improvement in ESSDAI score at week 12 was not significantly different between the RO5459072 and placebo arms (42.1% *vs* 37.8%, respectively; Table 2 and Supplementary Fig. S3, available at *Rheumatology* online).

**Table 2.** Primary and secondary efficacy outcomes at week 12

Endpoint	Placebo (*N* = 37)	RO5459072 (*N* = 38)
Primary efficacy endpoint		
ESSDAI responders, *n* (%)	14 (37.8)	16 (42.1)
95% CI	(20.86, 54.82)	(25.09, 59.12)
ESSDAI response rate^a^		
Difference in response rates (95% CI)	4.27 (–20.55, 29.08)	
*P*-value	0.7955	
Secondary efficacy endpoints		
ESSPRI responders, *n* (%)	21 (56.8)	22 (57.9)
ESSPRI response rate^b^		
Difference in response rates (95% CI)	1.14 (–23.92, 26.19)	
*P*-value	0.9877	
ESSDAI score		
Difference of adjusted means (95% CI)	–0.13 (–2.04, 1.78)	
*P*-value	0.8905	
ESSPRI score		
Difference of adjusted means (95% CI)	–0.22 (–1.08, 0.64)	
*P*-value	0.6077	
SF-36 Mental		
Difference of adjusted means (95% CI)	–2.06 (–5.87, 1.75)	
*P*-value	0.2846	
SF-36 Physical		
Difference of adjusted means (95% CI)	–0.33 (–2.43, 3.08)	
*P*-value	0.8134	
Unstimulated tear production		
Difference of adjusted means (95% CI)	0.87 (–1.30, 3.03)	
*P*-value	0.4266	
Mechanically stimulated salivary flow		
Difference of adjusted means (95% CI)	0.06 (–0.21, 0.34)	
*P*-value	0.6429	

Baseline is the patient’s last observation prior to initiation of study drug.

a Patients with ≥3 point reduction from baseline in ESSDAI at week 12 were classified as responders.

b Patients with ≥1 point reduction from baseline in ESSPRI at week 12 were classified as responders. Patients with a missing response were classed as non-responders. ESSDAI: EULAR SS Disease Activity Index; ESSPRI: EULAR SS Patient Reported Index; SF-36: Short Form-36 Health Survey.

There were no statistically significant differences between the RO5459072 and placebo arms for the secondary clinical endpoints (Table 2 and Supplementary Fig. S4, available at *Rheumatology* online). The difference in the mean change from baseline to week 12 in ESSDAI scores between the two groups was –0.13 (95% CI –2.04, 1.78; *P* = 0.8905). No significant differences were seen in any of the ESSDAI individual domains between baseline and week 12 for both groups (results not shown).

There was no significant difference between the placebo and RO5459072 groups in the proportion of ESSPRI responders (defined as patients with ≥1 point reduction from baseline in ESSPRI score at week 12; Table 2). No significant differences were seen in any of the ESSPRI individual domains (results not shown). No significant changes were seen in the SF-36 mental and physical component scores in either arm (Table 2).

The findings for unstimulated tear production and mechanically stimulated salivary flow likewise showed no significant differences. Although the RO5459072 group showed a smaller decline compared with the placebo group at week 12 for unstimulated tear production, these differences were not statistically significant (Table 2). At week 12, mechanically stimulated salivary flow in the RO5459072 group showed a slight (but non-significant) trend towards improvement over the placebo group (Table 2).

### Soluble biomarkers

**Figure 3.**
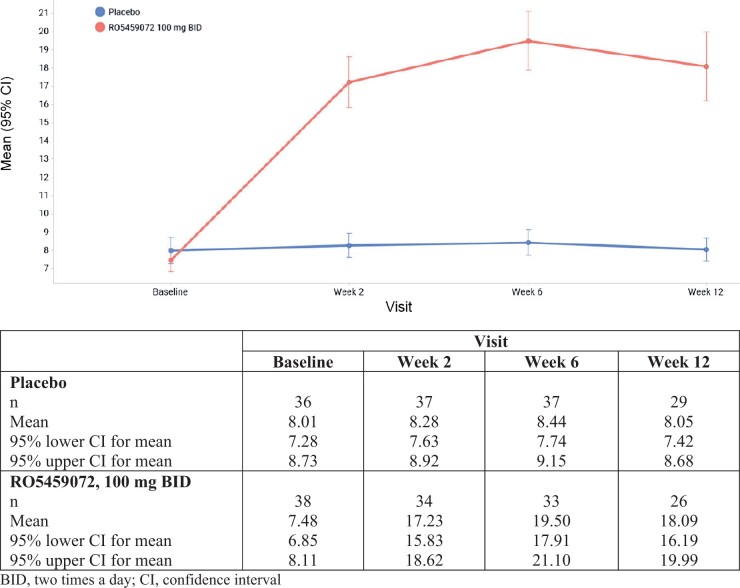
Concentration of plasma cathepsin S mass over the treatment period

Cathepsin S mass is a direct marker of target engagement by RO5459072 [19] and there was an increase in the plasma concentration of cathepsin S over the dosing period in the RO5459072 arm (Fig. 3). There was a 2.4-fold increase at week 12 compared with baseline in the RO5459072 group, with mean (s.d.) values ranging from 7.48 (1.91) ng/ml at baseline to 18.09 (4.71) ng/ml at week 12. No change in cathepsin S mass was observed in the placebo group (results not shown). Throughout the study there were no changes in the levels of cystatin C, an endogenous antagonist of cathepsin S (results not shown).

There was a modest decrease in mean (s.d.) IgG levels of −0.50 (1.13) in the RO5459072 arm compared with 0.48 (1.78) in the placebo arm (*P* = 0.010). Similar trends were seen for IgM with mean values of –0.17 (0.20) and 0.06 (0.25), respectively (*P* < 0.001). There was a modest transient increase in the levels of the cytokine BAFF in the RO5459072 arm compared with the placebo arm, with the mean (s.d.) value peaking at week 2 at 1179.8 (354.4) pg/ml, compared with a baseline value of 1072.7 (323.2) pg/ml. In contrast, levels of BAFF remained stable throughout the treatment period in the placebo group (results not shown). However, these differences were not statistically significant in either arm.

### Circulating white blood cells

There were modest changes in the levels of circulating white blood cells, including T cells, B cells and monocytes; however, the differences between the study arms were not significant due to high individual variability.

There was a small transient decrease in the absolute numbers of circulating B cells in the RO5459072 group, with a maximal reduction at week 2 [mean (s.d.) decrease of –20.3 (60.5) cells/µl]. By comparison, the placebo group showed a mean (s.d.) change from baseline to week 2 of 4.7 (49.8) cells/µl. The mean (s.d.) of the absolute values was 202.4 (117.2) cells/µl at baseline and 197.5 (107.5) cells/µl at week 12 in the placebo group; and 204.9 (114.0) cells/µl at baseline and 205.1 (132.3) cells/µl at week 12 in the RO5459072 group.

A slight decrease was also seen in the absolute numbers of circulating CD8+ T cells at the end of the treatment period (week 12) in the RO5459072 group. The mean (s.d.) change from baseline to week 12 was –4.2 (97.3) cells/µl in the placebo group and –42.1 (149.9) cells/µl in the RO5459072 group. In contrast, no differences were observed in the numbers of circulating CD4+ T cells in both study groups. The mean (s.d.) change from baseline to week 12 was 17.8 (197.3) cells/µl in the placebo group and 10 (188.9) cells/µl in the RO5459072 group.

For monocytes, there was a small increase in the absolute values over the dosing period in the RO5459072 group compared with the placebo group. The mean (s.d.) change from baseline at week 12 was –3.1 (107.8) cells/µl in the placebo group and 42.5 (80.6) cells/µl in the RO5459072 group.

### Salivary gland biopsies

Thirty-five biopsy samples from 19 patients (19 samples) at baseline (8 in the placebo group and 11 in RO5459072 group) and 16 samples after 12 weeks’ treatment (5 in the placebo group and 11 in the RO5459072 group) were processed for histological examination, including the change from baseline in focus score (number of lymphocytic foci per 4 mm^2^), mean foci area and percentage area of lymphocytic infiltration, and changes in the organization of lymphocytic foci. No significant differences in any of these parameters were observed in either arm (results not shown).

### Safety

The mean (s.d.) total cumulative dose of RO5459072 in this study was 14 247.4 (5343.8) mg, with a mean duration of treatment of 73 days in the RO5459072 arm and 82 days in the placebo arm.

Overall, treatment with RO5459072 at 200 mg daily (administered as 100 mg BID) over 12 weeks was well-tolerated (Table 3). There was one death (cardiac arrest in a patient in the placebo group). Three patients reported SAEs: two patients in the placebo group had a total of three SAEs (headache, metabolic acidosis and the incident of cardiac arrest, which led to death) and one patient in the RO5459072 group had one SAE (iron-deficiency anaemia). No SAEs were considered related to study treatment. Six patients had AEs that led to withdrawal from study treatment (all in the RO5459072 group); none was considered serious. The preferred terms were: chest discomfort, limb discomfort, palpitations, peripheral swelling, headache, pain in jaw, rash, pruritus, urticaria, abdominal pain and diarrhoea. Overall, the incidence of AEs was similar in both groups (Table 3). The majority of AEs were mild to moderate in severity. No obvious drug-related trends or signals were observed, and there were no clinically relevant changes in laboratory parameters, ECGs or vital sign measurements.

**Table 3.** Overview of adverse events

	Placebo (*N* = 37)	RO5459072 (*N* = 38)	All (*N* = 75)
	*n* (%)		
Number of patients with ≥1 adverse event	29 (78.4)	29 (76.3)	58 (77.3)
Total number of adverse events	68	113	181
Deaths	1 (2.7)	0	1 (1.3)
Withdrawals from the study due to an adverse event	0	6 (15.8)	6 (8.0)
Number of patients with ≥1:			
Adverse event with fatal outcome	1 (2.7)	0	1 (1.3)
Serious adverse event	2 (5.4)	1 (2.6)	3 (4.0)
Serious adverse event leading to withdrawal from treatment	0	0	0
Serious adverse event leading to dose modification/treatment interruption	0	0	0
Related serious adverse event	0	0	0
Adverse event leading to withdrawal from treatment	0	6 (15.8)	6 (8.0)
Adverse event leading to dose modification/treatment interruption	1 (2.7)	5 (13.2)	6 (8.0)
Related adverse event	8 (21.6)	20 (52.6)	28 (37.3)
Related adverse event leading to withdrawal from treatment	0	5 (13.2)	5 (6.7)
Related adverse event leading to dose modification/treatment interruption	1 (2.7)	5 (13.2)	6 (8.0)

Adverse events were encoded using MedDRA version 20.0. Multiple occurrences of the same adverse event in one individual were counted only once except for within the ‘Total number of adverse events’ row, in which multiple occurrences of the same adverse event were counted separately.

## Discussion

The primary endpoint of this study, defined as the proportion of patients with a ≥3 point reduction in ESSDAI score after 12 weeks’ treatment compared with baseline, was not met. The ESSDAI is a compound clinical scoring system that measures 12 domains which capture disease activity in SS [21, 23]. Although a mean reduction of ≥3 points was observed in around half of the RO5459072-treated patients, this response was unexpectedly matched by those in the placebo group. Similarly, no significant differences were observed in any of the individual ESSDAI domains. The high placebo responder rates could indicate the need to look at more rigorous cut-offs for ESSDAI response [27], although the absence of any difference in mean score over time makes a differential effect between the study arms unlikely.

Although the ESSDAI is widely used to measure disease activity in clinical studies, ensuring an accurate and reproducible rating of each domain remains a challenge [28]. The current means of scoring and weighting the 12 domains leaves much room for subjective scoring [28], thereby increasing the possibility of variance as we also observed, with a wide individual variation in ESSDAI results in both study arms. Furthermore, some individual domains, such as the one for the peripheral nervous system, may not have responded to treatment within the 12-week study period. Altogether, these factors may have contributed towards the study outcome.

In addition to the ESSDAI and ESSPRI, we also evaluated quality of life scores (SF-36 mental and physical components), unstimulated tear production, and mechanically stimulated salivary flow. The scores for both components of the SF-36 were similar between both study arms at the end of the treatment period. Both groups showed a decline in unstimulated tear production at week 12 compared with baseline; however, the RO5459072 group showed less of a decline compared with the placebo group. At week 12, the RO5459072 arm showed a slight trend towards improvement over the placebo arm with respect to mechanically stimulated salivary flow. However, the results for these secondary endpoints were not statistically significant.

Analysis of soluble biomarkers (including cathepsin S mass, cystatin C, 4-β-hydroxycholesterol and BAFF) was performed at baseline and throughout the 12-week treatment period. Over the treatment period, we observed a 2.4-fold increase in the concentration of cathepsin S mass (relative to baseline) in the RO5459072 arm, reaching a plateau at week 2 (Fig. 3). These findings suggest direct target engagement between RO5459072 and cathepsin S in those patients who received the study drug. There was no evidence of an increased endogenous inhibition effect as indicated by similar levels of plasma cystatin C, an endogenous antagonist of cathepsin S, in both study arms [29, 30].

Several other exploratory endpoints were measured in this study to assess the effect of RO5459072 on T cell–dependent activation of B cells. We observed a trend towards a modest and transient decrease in the number of B cells in the RO5459072 group (at week 2), and a small decrease in the number of CD8+ T cells at week 12. These observations are consistent with the expected effects of RO5459072 on MHC-II-mediated immune stimulation of adaptive T cells and B cells. No significant trends were observed in histological analyses of minor salivary gland biopsies. Given the lack of a clear signal for these parameters, our findings highlight the need for further studies to fully understand the mechanisms of MHC-II-mediated immune stimulation in pSS.

There are several limitations to our study. First, there is no formal consensus on the optimal outcome for evaluating treatment efficacy in pSS. Currently, the ESSDAI and ESSPRI are the only disease-specific validated clinical scores available for pSS [22, 24]. Although the ESSPRI has an acceptable reproducibility, the scores do not necessarily correlate with systemic disease activity [31]. pSS is characterized by considerable heterogeneity and its spectrum extends from secretory gland dysfunction to systemic involvement with extra-glandular manifestations. Thus, symptoms can range from mild sicca symptoms, pain, fatigue and arthralgia to more severe systemic manifestations such as arthritis, vasculitis or tubulo-interstitial nephritis. The breadth and complexity of these symptoms pose a challenge for capturing treatment effect and disease outcomes. Not surprisingly, our results showed a wide variation across all of the study endpoints. These may be a reflection of the complexity of the disease, compounded by the difficulties in detecting small changes using the currently available clinical scores. The heterogeneity in disease manifestations and high placebo responses with individual measures, seen also in other recent large multicentre trials [32–34], has led to proposals for composite outcomes [35, 36]; however, these require further validation and were not available at the time this study was designed and analysed. Other possible explanations for the high placebo responses seen include the use of concomitant medications, regression to the mean, and positive expectations given the lack of proven therapeutics in pSS. Another uncertainty is the timeframe in which to detect a treatment effect. Many studies in pSS have ranged in treatment duration between 6–24 weeks [37–40]; however, few studies have identified a clear treatment effect within these windows [33, 40–42]. Despite the changes seen in immunoglobulin levels in our study, it is uncertain if benefits in clinical or patient-reported outcomes would have been observed with a longer study timeframe. In other studies, a change in immunoglobulins was not associated with symptomatic improvement after 24 weeks’ treatment with HCQ or abatacept [32, 37]. Finally, the small size of our study population precluded further analysis of the observed differences between the study arms.

Presentation of antigenic peptide by MHC-II expressed on the surfaces of antigen-presenting cells is central for adaptive immune responses and for autoimmune diseases mediated by CD4+ T cells. Removal of the invariant chain bound to MHC-II is essential for both peptide loading and also for MHC-II to exit the endoplasmic reticulum for subsequent cell surface expression. Removal of the invariant chain is achieved by a number of proteolytic steps, the last being mediated by cathepsin S in B cells and dendritic cells [19]. Cathepsin S inhibition would therefore be anticipated to have efficacy in CD4+ T cell–mediated autoimmune diseases and amelioration of disease in models of SLE, SS, inflammatory arthritis and multiple sclerosis has been observed [14, 17, 43, 44]. Despite this, RO5459072 has also failed to show efficacy in psoriasis [45] and another cathepsin S inhibitor was ineffective in RA [46].

RO5459072 was well tolerated in our study population, albeit 15.8% of the RO5459072 arm discontinued treatment or withdrew. One death was reported (due to cardiac arrest in a placebo patient). No apparent drug-related trends or signals emerged in terms of AEs, ECG, vital signs and safety laboratory parameters.

### Conclusions

The primary endpoint of this study, the proportion of patients showing an improvement in ESSDAI score (≥3 points from baseline to week 12), was not met. RO5459072 was generally well-tolerated in patients with pSS. Further work is needed to clarify the role of cathepsin S and MHC-II-mediated immune stimulation in pSS.

## Supplementary Material

kead092_Supplementary_Data

## Data Availability

Clinical study documentation can be requested via the following link: https://www.roche.com/research_and_development/who_we_are_how_we_work/clinical_trials/our_commitment_to_data_sharing/clinical_study_documents_request_form.htm.
