# Supplementary material for: A randomized, double-blind, placebo-controlled, parallel group study on the effects of a cathepsin S inhibitor in primary Sjögren’s syndrome
Source: Rheumatology (Oxford). 2023 Mar 2;62(11):3644–53. doi: 10.1093/rheumatology/kead092 (PMC10629789; doi:10.1093/rheumatology/kead092)
Supplement: kead092_Supplementary_Data [file kead092_supplementary_data.zip › kead092_Supplementary_Data/rhe-22-2329-File003.pdf]

PROTOCOL

**TITLE:**	**A MULTI-CENTER, RANDOMIZED, DOUBLE-BLIND, PLACEBO-CONTROLLED, PARALLEL GROUP PHASE 2A STUDY TO ASSESS THE EFFICACY OF RO5459072 IN PATIENTS WITH PRIMARY SJÖGREN'S SYNDROME**
**PROTOCOL NUMBER:**	BP30037
**VERSION:**	3
**EUDRACT NUMBER:**	2015-004476-30
**IND NUMBER:**	128528
**TEST PRODUCT:**	RO5459072
**SPONSOR:**	F. Hoffmann-La Roche Ltd
**DATE FINAL:**	Version 1: 28 January 2016
**DATE AMENDED**	Version 2: 01 August 2016<br>Version 3: See electronic date stamp below

FINAL PROTOCOL APPROVAL

Approver's Name	Title	Date and Time (UTC)
Thomas,Dolca	Translational Medicine Leader	30-Oct-2016 15:40:47

CONFIDENTIAL STATEMENT

The information contained in this document, especially any unpublished data, is the property of F.Hoffmann-La Roche Ltd (or under its control) and therefore is provided to you in confidence as an investigator, potential investigator, or consultant, for review by you, your staff, and an applicable Ethics Committee or Institutional Review Board. It is understood that this information will not be disclosed to others without written authorization from Roche except to the extent necessary to obtain informed consent from persons to whom the drug may be administered.

PROTOCOL AMENDMENT, VERSION 3: RATIONALE

Protocol BP30037 has been amended to incorporate the following changes to the protocol:

Section 4.2.3: Implementation of changes to eligibility criteria as recommended by the US Food and Drug Administration (FDA).

The FDA strongly recommended that tuberculosis testing be considered at screening. The eligibility criteria of the protocol have therefore been amended to mandate testing for tuberculosis and exclude patients with positive results.

As per FDA feedback the eligibility criteria have also been amended to exclude women who are breast-feeding or planning to nurse.

The eligibility criteria have been amended to explicitly exclude patients who are using strong inhibitors of CYP3A4 and P-glycoprotein (P-gp), as well as compounds inducing CYP3A4. These medications were already specified as prohibited therapies. For consistency with changes to Section 4.5.2, patients using sensitive substrates of CYP3A4 with a narrow therapeutic window are also explicitly excluded.

Section 4.5.2: Update on the use of prohibited therapy as recommended by US FDA

Explicit prohibition of the use of sensitive substrates of CYP3A4 with a narrow therapeutic index has been added to existing cautionary statements about the concomitant use of CYP3A4 substrates.

Section 4.6.1.4: Addition of bicarbonate to laboratory test panel

The European League against Rheumatism (EULAR) Sjögren's Syndrome Disease Activity Index (ESSDAI) assessment includes evaluation of renal acidosis. Measurement of bicarbonate is important for diagnosis of renal acidosis and has consequently been added to the laboratory test panel.

Section 4.6.1.6: Addition of IgM to antibody titre measurements

The ESSDAI assessment includes, among others, evaluation of hypergammaglobulinemia.

Measurement of IgM is required for this assessment and hence total IgM has been added to the list of antibody titres to be measured. As samples for the above were not collected at all visits where ESSDAI was evaluated, additional assessment time points for cryoglobulin, IgG and IgM have been added to the Schedule of Assessment (SoA)

tables and the complement C3/4 assessment has been moved to the blood biochemistry panel.

Section 5.3.5.6: Liver Function Testing (LFTs)

A section on interpretation and reporting of abnormal liver function tests has been inserted to provide more specific guidance for investigators.

Section 6: Statistical Considerations and Analysis Plan

The alpha level for any hypothesis testing has been clarified as 2-sided at the 0.05 level to align with the use of 95% confidence intervals (CIs). Additionally the primary analysis will use a non-responder approach for missing data and an exploratory analysis was pre-specified adjusting for baseline ESSDAI score.

Section 6.7: Pharmacodynamic Analyses

This section has been modified to clarify that pharmacodynamic data will primarily be summarized and listed according to treatment actually received. Limited exploratory analyses employing methods used for efficacy endpoints on the mITT population may be carried out, but will be detailed in the statistical analysis plan.

PROTOCOL AMENDMENT, VERSION 3: SUMMARY OF CHANGES

PROTOCOL SYNOPSIS

The protocol synopsis has been updated to reflect the changes to the protocol, where applicable.

SECTION 3.2.6: Rationale for Pharmacodynamic Assessments

Elevated circulating titers of a range of auto-antibodies are reported in patients with primary Sjögren's syndrome (Tzioufas et al 2012), and as no single type is considered diagnostic, prognostic or predictive a panel including a representative cross-section will be measured, as well as total IgG*, IgM* and cryoglobulins (Quartuccio et al 2014).

SECTION 4.2.3: Exclusion Criteria

The exclusion criteria have been updated as follows:

6. A positive test result for hepatitis B (HBV), hepatitis C (HCV), or human immunodeficiency virus (HIV), ~~a history of~~*or* tuberculosis, or any other active viral, fungal, yeast or bacterial infection at the screening visit.

....

14. *Women who are lactating.*
15. *Use of other prohibited medication (moderate or potent inhibitors of CYP3A4; strong inducers of CYP3A4; strong inhibitors of the transporter P-glycoprotein [P-gp]; sensitive substrates of CYP3A4 with a narrow therapeutic index).*

SECTION 4.5.2: Prohibited Therapy

Because RO5459072 is a substrate of CYP3A4 and P-gp, other prohibited medications are:

- Moderate or potent inhibitors of CYP3A4 (e.g., itraconazole, erythromycin, nefazodone).
- Strong inducers of CYP3A4 (e.g., rifampicin, carbamazepine).
- *Sensitive substrates of CYP3A4 with a narrow therapeutic index (e.g., alfentanil, colchicine, ethosuxamide, fentanyl, and pimozide).*
- Strong inhibitors of the transporter P-glycoprotein (P-gp) (e.g., quinidine, clarithromycin).

A full list of prohibited concomitant medications is provided in Appendix 4.

SECTION 4.6.1.4: Laboratory Assessments

- *Blood*~~Serum~~ **chemistry:** Albumin, alkaline phosphatase (ALP), alanine aminotransferase (ALT), aspartate aminotransferase (AST), *bicarbonate,* C-reactive protein (CRP), calcium, chloride, creatinine phosphokinase (CPK), gamma-glutamyl transferase (GGT), glucose, phosphate, potassium, creatinine, sodium, total bilirubin, total protein, troponin, urea*, complement (C3 and C4),*
- **Coagulation:** activated partial thromboplastin time (aPTT), prothrombin time (PT)
- **Lipids:** High-density lipoprotein-cholesterol (HDL-C), low-density lipoprotein cholesterol (LDL-C), triglycerides, total cholesterol.
- **Urinalysis** A midstream urine specimen will be collected for dipstick analysis of protein, blood, glucose, and pH.
- <u>At Screening only</u>: HIV (specific tests HIV-1 antibody, HIV-1/2 antibody, HIV-2 antibody), hepatitis B surface antigen (HBsAg), total hepatitis B core antibody (HBcAb), hepatitis C virus (HCV) antibody*, tuberculosis testing*.

SECTION 4.6.1.6: Pharmacodynamic Assessments

Auto-antibodies (anti-SSA/SSB)

Serum will be collected for the determination of anti-SSA/SSB autoantibodies as indicated in the SoA (Appendix 1). These measurements will include anti-SSA, anti-SSB, ANA, and rheumatoid factor, as well as total IgG*, IgM* and cryoglobulins.

....

Soluble Biomarker Assessments

Plasma samples - Samples for measurement of biomarkers connected to Sjögren’s syndrome and extracellular matrix degradation will be collected at specified visits as indicated in the SoA (Appendix 1). Biomarkers to be measured will include~~ complement (C3 and C4),~~ β2-microglobulin, cytokines (including, but not limited to, BAFF, IL-4, IL-6 and IL-10), beta-hydroxycholesterol and desmosine.

SECTION 5.3.5.6: Abnormal Liver Function Tests

The finding of an elevated ALT or AST ($>3 \times$ ULN) in combination with either an elevated total bilirubin ($>2 \times$ ULN) or clinical jaundice in the absence of cholestasis or other causes of hyperbilirubinemia is considered to be an indicator of severe liver injury. Therefore, investigators must report as an adverse event the occurrence of either of the following:

- *Treatment-emergent ALT or AST $>3 \times$ ULN in combination with total bilirubin $>2 \times$ ULN*

- *Treatment-emergent ALT or AST > 3 × ULN in combination with clinical jaundice*

The most appropriate diagnosis or (if a diagnosis cannot be established) the abnormal laboratory values should be recorded on the Adverse Event eCRF (see Section 5.3.5.1) and reported to the Sponsor immediately (i.e., no more than 24 hours after learning of the event), either as a serious adverse event or a non-serious adverse event of special interest (see Section 5.4.2).

SECTION 6.1: Determination of Sample Size

A total of 70 patients will be randomized in a 1:1 ratio to RO5459072 or placebo (35 patients per treatment group). ~~Assuming a maximum drop-out rate of 15%, it is anticipated that enrollment of 70 patients would ensure at least 60 patients complete the study.~~

Seventy~~Sixty~~ patients (3*5*~~0~~ per treatment group) will provide *power in the range of* at least 58 *% to* ~~at least~~ 80 % ~~power~~ to detect a ~~33%~~ difference in ESSDAI response rates between treatment groups *in the range of 25 % to 33 %* (two-sided Chi-square test assuming alpha of 0.*05*~~15~~ and placebo rate ≤ 25%). *This sample size was determined by practical considerations.* S*eventy*~~ixty~~ patients are also sufficient to provide *approximately* ~~8~~*70*~~0~~% power to detect a difference of 3 points between treatment arms in change from baseline in ESSDAI score (t-test assuming two-sided alpha 0.*0*~~1~~5 and standard deviation of 5.0).

Approximately 30 patients will be enrolled into the optional sub-study. It is anticipated that enrollment of a minimum of 30 patients should ensure at least 20 patients (10 per treatment group) complete the study and have biopsy samples taken at both baseline and Week 12*, assuming a maximum drop-out rate of 15%*. ~~This sample size was determined by practical consideration.~~

SECTION 6.3.2: Pharmacodynamic Analysis Population

All patients who have received at least one dose of the study medication and who have pharmacodynamic data available will be included in pharmacodynamic analyses. For these analyses, patients will primarily be grouped according to the treatment actually received; unless otherwise specified in the statistical analysis plan.
~~Analyses of pharmacodynamic data will employ similar methods as used for continuous efficacy endpoints, i.e., analyses will employ the mITT population.~~

SECTION 6.6.1: Primary Efficacy Endpoint

The primary endpoint is the proportion of patients who have ≥ 3 point reduction from baseline in ESSDAI score after 12 weeks of treatment. The proportion of ~~subjects~~ *patients* who have ≥ 3 point reduction from baseline in ESSDAI score after 12 weeks of treatment will be compared between the two treatment arms using *a* Pearson Chi-square

test *(two sided p-values, alpha 0.05). The difference in proportions* ~~Two-sided p-values will be reported. Absolute reduction in risk~~ and corresponding 95% confidence interval (CI) will be provided. *Patients with missing data at Week 12 will be treated as non--responders in the analysis*~~Missing data will be imputed by last observation carried forward (LOCF) method~~.

Sensitivity analyses will be conducted if deemed necessary, including sensitivity analyses ~~for imputational method~~*for handling* ~~for~~ missing data (e.g*.,* ~~.,~~ ~~completers~~*completer's* analysis or *last observation carried forward (LOCF) method*~~imputing non-responders~~ for missing observations).

In addition; the proportion of patient~~subject~~s who have ≥ *3 point reduction from baseline in ESSDAI score after 12 weeks of treatment will be analyzed using a logistic regression model, adjusting for baseline ESSDAI score.*

SECTION 6.6.2: Secondary Efficacy Endpoint

The denominator degrees of freedom will be estimated using *the Kenward-Roger.*~~Satterthwaite's~~ approximation.

SECTION 6.7: PHARMACODYNAMIC ANALYSES

All pharmacodynamic parameters will be presented by listings and descriptive summary statistics separately by treatment group.

Pharmacodynamic variables at each assessment time-point along with corresponding changes from baseline will be listed by individual and summarized using descriptive summary statistics. Descriptive summary statistics will include the calculation of arithmetic means, standard deviation, coefficient variation, median, and range, as appropriate. Graphical plots of individual and mean absolute and change from baseline versus time profiles will also be produced for each treatment.

~~Analyses of pharmacodynamic data will employ similar methods as used for continuous efficacy endpoints, i.e., change from baseline at on-treatment visits will be compared between the treatment groups using a MMRM approach in the mITT population.~~

APPENDIX 1: Schedule of Assessments

The schedule of assessments has been revised to reflect the changes to the protocol.

APPENDIX 4: Prohibited Concomitant Medication

MDR1 (P-gp) inhibitors

Amiodarone, azithromycin, carvedilol, clarithromycin, dronedarone, erythromycin, fluvoxamine, indinavir, itraconazole, ketoconazole, lapatinib, propafenone, quercetin, quinidine, quinine, ranolazine, ritonavir (and ritonavir combinations), telaprevir, verapamil, vorapaxar.

Sensitive substrates of CYP3A4 with a narrow therapeutic index

Alfentanil, colchicine,ethosuxamide, fentanyl, pimozide.

Various sections:

Correction of minor inconsistencies between protocol sections and typographical errors has been done. Additional tuberculosis test at screening has been included. For clarity, tear and salivary flow assessments have been indicated in the SoA table (in line with salivary and tear sample collection time points), and additional biomarker sample collection time points (for the ESSDAI score) have been entered for the early termination/ follow-up visit in the SoA table. Furthermore, Figure 3 Study Design, was adjusted for improved clarity regarding the safety and follow-up visit.

MASTER INFORMED CONSENT FORM

The master Informed Consent Form been revised to reflect the changes to the protocol.

TABLE OF CONTENTS

SYNOPSIS OF PROTOCOL NUMBER BP30037 15

1. BACKGROUND AND RATIONALE 26

1.1 Background on Disease 26

1.2 Background on Cathepsin S 27

1.3 Background on RO5459072 28

1.3.1 Previous Non-Clinical Studies 28

1.3.1.1 Non-Clinical Pharmacology 28

1.3.1.2 Non-Clinical Pharmacokinetics and Product Metabolism 28

1.3.1.3 Toxicology and Safety Pharmacology 29

1.3.2 Previous Clinical Studies 30

1.3.2.1 Studies with RO5459072 30

1.3.2.2 Studies with Other Cathepsin S Inhibitors 34

1.4 Study Rationale and Benefit–Risk Assessment 34

1.4.1 Study Rationale 34

1.4.2 Benefit-Risk Assessment 35

2. OBJECTIVES 36

2.1 Primary Objectives 36

2.2 Secondary Objectives 37

2.3 Exploratory Objectives 37

2.4 Optional Sub-study Objective 37

3. STUDY DESIGN 38

3.1 Description of Study 38

3.1.1 Overview of Study Design 38

3.1.2 End of Study 39

3.2 Rationale for Study Design 39

3.2.1 Rationale for Dosage Selection 39

3.2.2 Rationale for Study Population 40

3.2.3 Rationale for Control Group 41

3.2.4 Rationale for Design 42

3.2.5 Rationale for Efficacy Assessments 42

3.2.6 Rationale for Pharmacodynamic Assessments 43
3.2.7 Rationale for Pharmacokinetic Assessments......................... 44
3.2.8 Rationale for Safety Assessments.. 44
3.2.9 Rationale for Other Assessments 44
3.2.10 Rationale for Biopsy Sub-study 44
3.3 Outcome Measures 44
3.3.1 Efficacy Outcome Measures.. 44
3.3.2 Pharmacodynamic Outcome Measures................................ 45
3.3.3 Pharmacokinetic Outcome Measures................................... 45
3.3.4 Safety Outcome Measures 45
3.3.5 Exploratory Outcome Measures 46
3.3.6 Optional Sub-study Outcome Measures............................... 46

4. MATERIALS AND METHODS 46
4.1 Centers 46
4.2 Study Population 46
4.2.1 Recruitment Procedures 47
4.2.2 Inclusion Criteria 47
4.2.3 Exclusion Criteria 47
4.3 Method of Treatment Assignment and Blinding 48
4.3.1 Treatment Assignment... 48
4.3.2 Blinding 48
4.3.3 Emergency Unblinding... 48
4.4 Study Treatment 49
4.4.1 Formulation, Packaging, and Handling 49
4.4.1.1 RO5459072 and Placebo 49
4.4.2 Dosage, Administration, and Compliance.............................. 50
4.4.2.1 RO5459072 and Placebo 50
4.4.3 Investigational Medicinal Product Accountability 50
4.4.4 Post-Trial Access to RO5459072 51
4.5 Concomitant Therapy and Food 51
4.5.1 Permitted Therapy 51
4.5.2 Prohibited Therapy 52
4.5.3 Vaccination 53

4.5.4 Food ... 53
4.5.5 Contraception ... 53
4.6 Study Assessments ... 54
4.6.1 Description of Study Assessments ... 54
4.6.1.1 Medical History and Demographic Data ... 54
4.6.1.2 Physical Examinations ... 54
4.6.1.3 Disease-Specific Assessments ... 55
4.6.1.4 Laboratory Assessments ... 56
4.6.1.5 Pharmacokinetic Assessments ... 57
4.6.1.6 Pharmacodynamic Assessments ... 57
4.6.1.7 Clinical Genotyping ... 59
4.6.1.8 Electrocardiograms ... 59
4.6.1.9 Vital Signs ... 60
4.6.1.10 Administering the SF36 and ESSPRI (Patient-Reported Outcomes) ... 60
4.6.1.11 MSG Biopsies (Optional Sub-study) ... 60
4.6.1.12 Timing of Assessments - Screening and Pretreatment Assessments ... 61
4.7 Patient, Study, and Site Discontinuation ... 61
4.7.1 Patient Discontinuation ... 61
4.7.2 Study and Site Discontinuation ... 62
5. ASSESSMENT OF SAFETY ... 62
5.1 Safety Parameters and Definitions ... 62
5.1.1 Adverse Events ... 63
5.1.2 Serious Adverse Events (Immediately Reportable to the Sponsor) ... 63
5.1.3 Non-Serious Adverse Events of Special Interest (Immediately Reportable to the Sponsor) ... 64
5.2 Safety Plan ... 64
5.2.1 Management of Specific Adverse Events ... 64
5.3 Methods and Timing for Capturing and Assessing Safety Parameters ... 64
5.3.1 Adverse Event Reporting Period ... 65
5.3.2 Eliciting Adverse Event Information ... 65

5.3.3 Assessment of Severity of Adverse Events ... 65
5.3.4 Assessment of Causality of Adverse Events ... 65
5.3.5 Procedures for Recording Adverse Events ... 66
5.3.5.1 Diagnosis versus Signs and Symptoms ... 66
5.3.5.2 Adverse Events Occurring Secondary to Other Events ... 66
5.3.5.3 Persistent or Recurrent Adverse Events ... 67
5.3.5.4 Abnormal Laboratory Values ... 67
5.3.5.5 Abnormal Vital Sign Values ... 68
5.3.5.6 Abnormal Liver Function Tests ... 68
5.3.5.7 Deaths ... 69
5.3.5.8 Preexisting Medical Conditions ... 69
5.3.5.9 Hospitalization or Prolonged Hospitalization ... 69
5.3.5.10 Overdoses ... 70
5.4 Immediate Reporting Requirements from Investigator to Sponsor ... 70
5.4.1 Emergency Medical Contacts ... 71
5.4.2 Reporting Requirements for Serious Adverse Events and Non-Serious Adverse Events of Special Interest ... 71
5.4.3 Reporting Requirements for Pregnancies ... 71
5.4.3.1 Pregnancies in Female Patients ... 71
5.4.3.2 Pregnancies in Female Partners of Male Patient ... 71
5.4.3.3 Abortions ... 72
5.4.3.4 Congenital Anomalies/Birth Defects ... 72
5.5 Follow-Up of Patients after Adverse Events ... 72
5.5.1 Investigator Follow-Up ... 72
5.5.2 Sponsor Follow-Up ... 72
5.6 Post-Study Adverse Events ... 73
5.7 Expedited Reporting to Health Authorities, Investigators, Institutional Review Boards, and Ethics Committees ... 73

6. STATISTICAL CONSIDERATIONS AND ANALYSIS PLAN ... 73
6.1 Determination of Sample Size ... 74

6.2 Summaries of Conduct of Study ... 74
6.3 Analysis Populations ... 74
6.3.1 Efficacy Analysis Population ... 74
6.3.2 Pharmacodynamic Analysis Population ... 74
6.3.3 Pharmacokinetic Analysis Population ... 75
6.3.4 Safety Analysis Population ... 75
6.4 Summaries of Treatment Group Comparability ... 75
6.5 Safety Analyses ... 75
6.5.1 Adverse Events ... 75
6.5.2 Clinical Laboratory Test Results ... 75
6.5.2.1 Standard Reference Ranges and Transformation of Data ... 76
6.5.2.2 Definition of Laboratory Abnormalities ... 76
6.5.3 Vital Signs ... 76
6.5.4 ECG Data Analysis ... 76
6.5.5 Concomitant Medications ... 77
6.6 Efficacy Analyses ... 77
6.6.1 Primary Efficacy Endpoint ... 77
6.6.2 Secondary Efficacy Endpoints ... 77
6.7 Pharmacodynamic Analyses ... 78
6.8 Pharmacokinetic Analyses ... 78
6.9 Exploratory Analyses ... 78
6.10 Interim Analyses ... 78

7. DATA COLLECTION AND MANAGEMENT ... 78
7.1 Data Quality Assurance ... 78
7.2 Electronic Case Report Forms ... 79
7.3 Patient-Reported Outcome Data ... 79
7.4 Source Data Documentation ... 79
7.5 Use of Computerized Systems ... 80
7.6 Retention of Records ... 80

8. ETHICAL CONSIDERATIONS ... 80
8.1 Compliance with Laws and Regulations ... 80
8.2 Informed Consent ... 81

8.3 Institutional Review Board or Ethics Committee 82
8.4 Confidentiality 82
8.5 Financial Disclosure 82

9. STUDY DOCUMENTATION, MONITORING, AND ADMINISTRATION 83
9.1 Study Documentation 83
9.2 Site Inspections 83
9.3 Administrative Structure... 83
9.4 Publication of Data and Protection of Trade Secrets 83
9.5 Protocol Amendments 84

10. REFERENCES 85

LIST OF TABLES

Table 1 Summary RO5459072 Pharmacokinetic Parameters at Steady-State after Administration with Food 33
Table 2 Adverse Event Severity Grading Scale 65

LIST OF FIGURES

Figure 1 Mean Linear RO5459072 Plasma Concentration versus Time Profiles after a Single Dose and at Steady-State after Twice Daily Administration with Food 32
Figure 2 Mean Change from Baseline in p10 Stimulation Index versus Time Profiles after a Single Oral Dose and after Repeat Dosing with Food 33
Figure 3 Study Design.. 39
Figure 4 Simulation of cathepsin S enzyme inhibition over the dosing interval based on population pharmacokinetic/ pharmacodynamic model. 40

LIST OF APPENDICES

Appendix 1 Schedule of Assessments... 89
Appendix 2 Disease-Specific Assessments 91
Appendix 3 Dose-Equivalents of Systemic Corticosteroids............................ 93
Appendix 4 Prohibited Concomitant Medications... 94

SYNOPSIS OF PROTOCOL NUMBER BP30037

**TITLE:**	**A MULTI-CENTER, RANDOMIZED, DOUBLE-BLIND, PLACEBO-CONTROLLED, PARALLEL GROUP PHASE 2A STUDY TO ASSESS THE EFFICACY OF RO5459072 IN PATIENTS WITH PRIMARY SJÖGREN'S SYNDROME**
**PROTOCOL NUMBER:**	BP30037
**VERSION:**	3
**EUDRACT NUMBER:**	2015-004476-30
**IND NUMBER:**	128528
**TEST PRODUCT:**	RO5459072
**PHASE:**	2A
**INDICATION:**	Primary Sjögren's Syndrome
**SPONSOR:**	F. Hoffmann-La Roche Ltd

OBJECTIVES

Primary Objective:

- To investigate the effects of RO5459072 treatment on disease activity and symptoms of primary Sjögren's syndrome.

Secondary Objectives

To investigate the effects of RO5459072 treatment on:

- quality of life measures.
- auto-antibody concentrations.
- pharmacodynamic measures of exocrine gland function.

Additionally:

- To collect samples for population modelling of RO5459072 pharmacokinetics.
- To investigate the safety and tolerability of RO5459072 treatment.

Exploratory Objectives

To investigate the effects of RO5459072 treatment on:

- cathepsin S enzyme mass and activity.
- sub-populations of circulating white blood cells.
- biomarkers connected to Sjögren's syndrome.
- biomarkers of extracellular matrix degradation.

Additionally:

- To explore the influence of covariates on the effects of RO5459072 treatment.
- To explore alternative composite endpoints as measures for evaluating effects of drug treatment.
- To explore the effects of RO5459072 treatment on the use of concomitant medications.
- To explore the occurrence of acute rebound phenomena on disease activity and symptoms following discontinuation of study drug treatment.

Optional Sub-study

The objective of the optional sub-study is:

To investigate the effects of RO5459072 treatment on salivary gland structure, inflammation and the organization of inflammatory foci assessed by histology and immunohistochemistry of labial biopsy samples.

STUDY DESIGN

Description of Study

Study BP30037 is a randomized, double-blind, placebo-controlled, two-treatment arm, parallel-group study designed to evaluate the effects of RO5459072 treatment on disease activity and symptoms of Sjögren's syndrome in adult patients with moderate to severe primary Sjögren's Syndrome. Patients will be randomized 1:1 to receive either 100 mg RO5459072 or placebo orally twice daily (b.i.d.). Patients will be treated for a maximum of 12 weeks.

Assessments of Sjögren's syndrome disease activity, symptoms and quality of life will be made before, during the study treatment phase and upon completion in order to evaluate the efficacy of RO5459072 treatment. Patients should start study drug treatment one day after the baseline visit. In addition, patients will undergo pharmacodynamic assessments of exocrine gland function and provide samples for measurement of white blood cell numbers and levels of biomarkers associated with Sjögren's syndrome or connected to the mechanism of action of RO5459072, as well as RO5459072 plasma concentrations. Safety and tolerability will be assessed throughout the study.

At selected participating study centers, patients may also enroll into an optional sub-study in which consenting individuals will undergo minor salivary gland (MSG) biopsies for assessing histological changes in labial salivary glands. Biopsies will be taken at baseline and at the end of the study drug treatment period.

Patients may concomitantly receive standard of care symptomatic treatment. Active or recent immunomodulatory therapy will not be allowed.

NUMBER OF PATIENTS

A total of 70 patients will be randomized in a 1:1 ratio to RO5459072 or placebo (35 patients per treatment group; approximately 30 patients will be enrolled into the optional sub-study).

TARGET POPULATION

Adults aged 18 to 75 years with moderate to severe primary Sjögren's syndrome.

INCLUSION/EXCLUSION CRITERIA

Inclusion criteria:

Patients must meet the following criteria for study entry:

1. Males and females 18 to 75 years of age, inclusive.
2. Patient willing to give written informed consent and to comply with the study procedures and restrictions.
3. Primary Sjögren's syndrome diagnosed previously according to the revised American-European Consensus Group (AECG) criteria.
4. European League against Rheumatism (EULAR) Sjögren's Syndrome Disease Activity Index (ESSDAI) score ≥ 5.
5. EULAR Sjögren's Syndrome Patient Reported Index (ESSPRI) score ≥ 5.
6. Elevated serum titers of anti-Sjögren's-syndrome-related antigen A (anti-SSA) and/or anti-SSB antibodies at screening.
7. Negative pregnancy test at screening and baseline (women only), and agreement to comply with measures to prevent pregnancy and restrictions on sperm donation (see Section 4.5.5).

Exclusion criteria:

Patients who meet any of the following criteria will be excluded from study entry:

1. A diagnosis of secondary Sjögren's syndrome according to the revised AECG criteria.
2. Severe complications of Sjögren's syndrome, such as vasculitis with renal, neurologic or cardiac involvement; interstitial lung disease and severe myositis.
3. Systemic immunosuppressant therapy (e.g., abatacept, azathioprine, ciclosporin, etanercept, tofacitinib), cyclophosphamide or B cell depleting therapy (e.g., rituximab anti-CD20 therapy) within 6 months prior to the screening visit. Low dose methotrexate treatment (i.e., oral doses ≤ 20 mg weekly) is, however, permitted.
4. Corticosteroid therapy exceeding 7.5 mg prednisone equivalents per day.
5. Mechanically stimulated whole salivary flow rate at baseline of < 0.1 mL/min.
6. A positive test result for hepatitis B (HBV), hepatitis C (HCV), or human immunodeficiency virus (HIV), *or* tuberculosis, or any other active viral, fungal, yeast or bacterial infection at the screening visit.
7. A history of recurring or chronic infections, any other indication of reduced immune function considered to be clinically significant by the Investigator, or any other underlying conditions which may further predispose patients to serious infection.
8. A history of lymphoma, myeloma (monoclonal hypergammaglobulinemia) or monoclonal gammopathy of unknown significance (MGUS), or any other malignancies within the past 5 years (except basal cell or squamous cell carcinoma of the skin that has been cured).
9. A diagnosis of fibromyalgia, or a diagnosis of significant depression or anxiety that in the opinion of the investigator would confound the interpretation of the study results.
10. Severe renal impairment (e.g., estimated glomerular filtration rate < 30 mL/min), moderate or severe hepatic impairment (e.g., Child-Pugh B or C) or other clinically significant hepatic disease (e.g., AST, ALT or GGT > 2x upper limit of normal).
11. Current or a history of severe, progressive or uncontrolled hematologic, gastrointestinal, endocrine, pulmonary, cardiac, or neurologic disease, or any other concomitant disease or condition or any clinically significant finding at screening that could interfere with, or for which the treatment of might interfere with, the conduct of the study, or that would, in the opinion of the Investigator, pose an unacceptable risk to the individual in this study.
12. Participation in an investigational drug or device study within 3 months prior to screening.
13. Inability to comply with the study protocol for any other reason.
14. *Women who are lactating, breastfeeding or planning to nurse.*
15. *Using other prohibited medication (moderate or potent inhibitors of CYP3A4; strong inducers of CYP3A4; strong inhibitors of the transporter P-glycoprotein [P-gp]; sensitive substrates of CYP3A4 with a narrow therapeutic index).*

LENGTH OF STUDY

The total duration of the study for each patient will be approximately 18 weeks (screening visit to follow-up visit). For each subject the study will consist of:

- Screening visit, up to 4 weeks before the first dose of study drug.

- Baseline visit, immediately preceding the first dose of study drug.
- A 12-week double-blind treatment period, and including three study center visits.
- A follow-up visit, 2 weeks after the last dose of study drug.

END OF STUDY

The end of the study is defined as the date of the last study center visit of the last patient participating in the study (includes the safety follow-up visit).

OUTCOME MEASURES

EFFICACY OUTCOME MEASURES

Efficacy assessments will include the ESSDAI, ESSPRI, and Short Form-36 Health Survey (SF-36). ESSDAI and ESSPRI will also be measured at screening in order to assess eligibility for the study.

The primary efficacy outcome for this study is:

- Proportion of patients showing a clinically relevant decrease in ESSDAI score after 12 weeks from baseline.

where a clinically relevant decrease in ESSDAI score is defined as a decrease of ≥ 3 points.

Secondary efficacy outcomes for this study are as follows:

- Proportion of individuals showing a clinically relevant decrease in ESSPRI score after 12 weeks where a clinically relevant decrease in ESSPRI score is defined as a decrease of ≥ 1 point.
- Change from baseline in ESSDAI, ESSPRI and SF-36 scores after 12 weeks.
- Change from baseline in each of the individual components of the ESSPRI (dryness, fatigue and pain) after 12 weeks.

PHARMACODYNAMIC OUTCOME MEASURES

The pharmacodynamics outcome measures for this study include:

- Tear flow rate using the Schirmer's test will be measured to assess the change from baseline.
- Mechanically stimulated salivary flow rate will be measured to assess the change from baseline.
- Measurement of serum auto-antibody titers from baseline which will include anti-SSA, anti-SSB, anti-nuclear antibodies, and rheumatoid factor, as well as total IgG, *IgM* and cryoglobulins. Auto-antibody titers (anti-SSA, anti-SSB) will also be measured at screening in order to assess eligibility for the study.
- Erythrocyte sedimentation test (ESR) will be measured to assess the change from baseline.
- Change from baseline in cathepsin S mass and activity in plasma, tears and saliva.
- The collection and measurement of circulating numbers of sub-populations of white blood cells from baseline, which will be counted by fluorescence-activated cell sorting (FACS) immunophenotyping of cell surface markers and may include TBNK/monocytes, T_H and T_{FH} cells and B cells.
- Biomarkers connected to Sjögren's syndrome and extracellular matrix degradation will be measured to assess the change from baseline in biomarker concentrations. Biomarkers to be measured will include complement (C3 and C4), β2-microglobulin, cytokines (including - but not limited to - BAFF, IL-4, IL-6 and IL-10), beta-hydroxycholesterol and desmosine.

SAFETY OUTCOME MEASURES

Safety assessments will include recording of adverse events (AEs) and concomitant medication use throughout the study, measurement of vital signs and triplicate 12-lead ECGs and laboratory safety testing.

Laboratory safety tests will include:

- Hematology: Hemoglobin, hematocrit, platelet count, red blood cell (RBC) count, mean corpuscular volume, absolute reticulocyte count, total and differential leucocyte (WBC) absolute count (neutrophils, eosinophils, lymphocytes, monocytes and basophils).
- *Blood* chemistry: Albumin, alkaline phosphatase (ALP), alanine aminotransferase (ALT), *bicarbonate,* aspartate aminotransferase (AST), C-reactive protein (CRP), calcium, chloride, creatinine phosphokinase (CPK), gamma-glutamyl transferase (GGT), glucose, phosphate, potassium, creatinine, sodium, total bilirubin, total protein, troponin, urea*, complement (C3 and C4)*.
- Coagulation: activated partial thromboplastin time (aPTT), prothrombin time (PT)
- Lipids: High-density lipoprotein-cholesterol (HDL-C), low-density lipoprotein cholesterol (LDL-C), triglycerides, total cholesterol.
- Urinalysis: A midstream urine specimen will be collected for dipstick analysis of protein, blood, glucose, and pH.

The safety outcome measures for this study are as follows:

- The incidence and severity of AEs.
- The incidence of SAEs or withdrawals because of AEs.
- The incidence of out-of-reference-range values for vital signs and ECGs, and laboratory tests.
- Change from baseline in vital signs and ECGs, and laboratory tests by visit.

PHARMACOKINETIC OUTCOME MEASURES

RO5459072 plasma concentration data will be pooled with data from other studies (Study WP29542 and Study BP29772) for population pharmacokinetic modelling.

EXPLORATORY OUTCOME MEASURES

Exploratory assessments will include collection of samples for genotyping, including human leukocyte antigen (HLA) haplotyping, at baseline.

The exploratory outcomes for this study are as follows:

- Comparisons of the performance of alternative composite endpoints (e.g., Sjögren’s response index [Sjögren’s syndromeRI-30]) as predictive markers of drug treatment response.
- Change from Week 12 to follow-up for ESSDAI, ESSPRI and SF-36 scores in each treatment group.
- Comparisons of drug treatment effects between genotypes.
- Patterns of use of selected concomitant medications.

OPTIONAL SUB-STUDY OUTCOME MEASURES

Patients consenting to also participate in the optional sub-study will undergo minor salivary gland (MSG) biopsies at baseline and Week 12. Biopsy samples will be processed for histological examination.

The outcomes for the optional sub-study are as follows:

- Change from baseline in focus score (number of lymphocytes per *4* mm^2).
- Change from baseline in numbers of defined sub-populations of infiltrating lymphocytes.
- Subjective evaluation of changes from baseline in minor salivary gland (MSG) structure.

Other exploratory analyses may include, but not be limited to, changes in leukocyte and stromal subpopulation numbers and degree of activation.

INVESTIGATIONAL MEDICINAL PRODUCT(S)

The investigational medicinal products in this study are:

- RO5459072 50 mg capsules for oral administration (Formulation F03)
- Placebo capsules for oral administration (Formulation F04).

All IMPs will be supplied and packaged by the Sponsor.

Study drugs are to be taken orally twice daily (b.i.d.) with food, i.e., with breakfast in the morning and a meal in the evening, for 12 weeks. Capsules should be swallowed whole.

Patients will be randomly assigned to one of two possible blinded study drug treatments:

- 100 mg RO5459072 (2 x F03 capsules) b.i.d. with food for 12 weeks (Weeks 1 to 12).
- Placebo (2 x F04 capsules) b.i.d. with food for 12 weeks (Weeks 1 to 12).

PROCEDURES

A Schedule of Assessments (SoA) is provided (see Appendix 1).

STATISTICAL METHODS

EFFICACY ANALYSES

Efficacy analyses will include modified intent-to-treat (mITT) population which is defined as all randomized *patients* who received at least one dose of double-blind study medication and have evaluable measurement of the parameter of interest at baseline and at least at one post-baseline visit.

The proportion of *patients* who have ≥3-point reduction from baseline in ESSDAI score after 12 weeks of treatment will be compared between the two treatment arms using *a* Pearson Chi-square test *(t*wo-sided p-values*, alpha 0.05*)*. The difference in proportions* and corresponding 95% CI will be provided. Supportive analyses will be conducted if deemed necessary. *Patients with missing data at Week 12 will be treated as non-responders in the analysis. Other* methods of imputation for missing data will be described in the protocol and statistical analyses plan.

Secondary endpoints that are binary will be analyzed using similar methods used for analyzing the primary endpoint.

Continuous secondary endpoints will be analyzed using a Mixed Model for Repeated Measures (MMRM) approach incorporating all observed data up to 12 weeks of treatment. The MMRM will include the absolute change from baseline as the dependent variable. The fixed effects in the model will include treatment, visit (week), treatment-by-visit (weeks) interaction, along with baseline score as continuous covariates. An unstructured variance-covariance structure will be applied to the model.

Additional exploratory analyses will be conducted if deemed appropriate.

PHARMACODYNAMIC ANALYSES

All pharmacodynamic parameters will be presented by listings and descriptive summary statistics separately by treatment group.

PHARMACOKINETIC ANALYSES

The population methods used for modelling of plasma concentration data will be described separately.

SAFETY ANALYSES

Safety data presentations will include all subjects who received at least one dose of the study medication. For these analyses, patients will be grouped according to the treatment actually received.

SAMPLE SIZE JUSTIFICATION

A total of 70 patients will be randomized in a 1:1 ratio to RO5459072 or placebo treatment groups.

A total of 70 patients (35 per treatment group) will provide *a power in the range of at least 58 % to 80 %* to detect a difference in ESSDAI response rates between treatment groups *in the range of 25 % to 33 %* (two-sided Chi-square test assuming alpha of 0. 05 and placebo response rate ≤ 25%). *This sample size was determined by practical considerations.* S*eventy* patients also provides approximately 70% power to detect a difference of 3-points between treatment groups in change from baseline in ESSDAI score (t test assuming two-sided alpha of 0.05 and standard deviation of 5.0).

Approximately 30 patients will be enrolled into the optional sub-study. It is anticipated that enrollment of 30 patients should ensure at least 20 patients (10 per treatment group) complete the study and have biopsy samples taken at both baseline and Week 12, *assuming a maximum drop-out rate of 15%*.

CONCOMITANT MEDICATIONS

In addition to the study drug treatment, patients may continue to concomitantly use existing treatments for Sjögren's syndrome.

Prohibited therapies are:

- Corticosteroid therapy exceeding 7.5 mg prednisone equivalent per day.
- Anti-CD20 therapy (e.g., rituximab) or other B cell-depleting therapy within 6 months of screening.
- Immunosuppressant therapy (e.g., abatacept, azathioprine, ciclosporin, etanercept, tofacitinib). Low dose methotrexate treatment (oral doses ≤ 20 mg weekly) is, however, permitted.
- Cyclophosphamide.

Systemic immunomodulatory therapy (e.g, hydroxychloroquine), low dose corticosteroids, or low dose methotrexate, or topically administered immunosuppressive agents (e.g., cyclosporine eye drops) are permissible providing the patient has been on a stable dose for ≥ 2 months prior to screening and the dose remains unchanged during the study. Similarly patients on non-steroidal anti-inflammatory drugs (NSAIDs), antidepressants, or pilocarpine must have been on a stable dose for 4 weeks prior to screening and expected to remain on this dose throughout the study. If any of these drugs have been discontinued recently, patients must be off drug for at least 8 weeks before screening.

Pilocarpine or drugs with similar pharmacological action should not be used within 12 hours of the assessment visit.

Other prohibited medications are:

- Moderate or potent inhibitors of CYP3A4 (e.g., itraconazole, erythromycin, nefazodone).
- Strong inducers of CYP3A4 (e.g., rifampicin, carbamazepine).
- *Sensitive substrates of CYP3A4 with a narrow therapeutic index (e.g., alfentanil, colchicine, ethosuxamide, fentanyl, and pimozide).*
- Strong inhibitors of the transporter P-glycoprotein (P-gp) (e.g., quinidine, clarithromycin).

CONTRACEPTION

Women of childbearing potential and men with female partners of childbearing potential must agree to remain abstinent (refrain from heterosexual intercourse) or use contraceptive methods that result in a failure rate of $< 1\%$ per year throughout the treatment period and for a specified period after the last dose of study treatment. Men with female partners of childbearing potential, or pregnant female partners, must remain abstinent or use contraceptive measures throughout the treatment period and for a specified period after the last dose of study treatment. Men must also refrain from donating sperm for specified periods. Women of childbearing potential will be required to undergo urine and serum pregnancy testing at each study center visit as defined in the SoA (see Section 4.5.5 and Appendix 1).

LIST OF ABBREVIATIONS AND DEFINITIONS OF TERMS

Abbreviation	Definition
**AE**	Adverse event
**AECG**	American-European Consensus Group
**ALT**	Alanine aminotransferase
**aPTT**	Activated partial thromboplastin time
**AST**	Aspartate aminotransferase
**AUC**	Area under the curve
**BP**	Blood pressure
**CNS**	Central nervous system
**CRO**	Contract research organization
**CSAP**	Clinical statistical analysis plan
**CSR**	Clinical study report
**CT**	Computed tomography
**CTCAE**	Common terminology criteria for adverse events
**DMARDs**	Disease-modifying anti-rheumatic drugs
**DNA**	Deoxyribonucleic acid
**DRF**	Dose-range-finding
**EC**	Ethics Committee
**ECG**	Electrocardiograms
**eCRF**	Electronic case report form
**EDC**	Electronic data capture
**ePRO**	Electronic patient-reported outcome
**EFD**	Embryo-fetal development
**ESF**	Eligibility screening form
**ESR**	Erythrocyte sedimentary test
**ESSDAI**	European League against Rheumatism (EULAR) Sjögren's Syndrome Disease Activity Index
**ESSPRI**	EULAR Sjögren’s Syndrome Patient Reported Index
**EU**	European Union
**FACS**	Fluorescence-activated cell sorting
**FDA**	Food and Drug Administration
**hERG**	Human *Ether-à-go-go*-Related Gene
**HBsAG**	Hepatitis B surface antigen
**HBcAb**	Total hepatitis B core antibody
**HCV**	Hepatitis C
**HDL**	High-density lipoproteins

Abbreviation	Definition
**HIPAA**	Health Insurance Portability and Accountability Act
**HIV**	Human immunodeficiency virus
**HLA**	Human leukocyte antigen
**IB**	Investigator's Brochure
**ICH**	International Conference on Harmonisation
**IMP**	Investigational medicinal product
**IND**	Investigational New Drug (application)
**INR**	International normalized ratio
**IRB**	Institutional Review Board
**IUD**	Intrauterine device
**IV**	Intravenous
**IxRS**	Interactive (voice/web) response system
**LC-MS/MS**	Liquid chromatography coupled to tandem mass spectrometry
**LDH**	Lactate dehydrogenase
**LDL**	Low-density lipoproteins
**LOCF**	Last observation carried forward
**LPLV**	Last patient, last visit
**LSLO**	Last study subject, last observation
**MAD**	Multiple-ascending doses
**MD**	Multiple-doses
**MHC**	Major histocompatibility complex
**mITT**	Modified intent-to-treat
**MMRM**	Mixed model for repeated measures
**MRI**	Magnetic resonance imaging
**mRNA**	Messenger ribonucleic acid
**MSG**	Minor salivary gland
**NOAEL**	No-observed-adverse-effect level
**OTC**	Over-the-counter
**PNS**	Peripheral nervous system
**PRO**	Patient-reported outcome
**PT**	Prothrombin time
**QRS**	QRS Complex
**QT**	QT Interval
**QTc**	QT Corrected for heart rate
**QTcF**	QT Corrected for heart rate using the Fridericia's correction factor

Abbreviation	Definition
**RBC**	Red blood cell
**RNA**	Ribonucleic acid
**SAE**	Serious adverse event
**SoA**	Schedule of assessments
**SPA**	Statistical Programmer
**ULN**	Upper limit of normal
**WBC**	White blood cell

1. BACKGROUND AND RATIONALE

1.1 BACKGROUND ON DISEASE

Sjögren's syndrome is one of the most common rheumatic autoimmune disorders (Fox et al 2000). The estimated prevalence, which differs considerably depending on type of study, diagnostic criteria and other variables between countries ranges from 0.2 to 2.7% (Patel et al 2014). It is a chronic systemic autoimmune disease in which immune-mediated inflammation, characterized by lymphocytic infiltration of exocrine glands and epithelia, causes secretory gland dysfunction leading to dryness of the main mucosal surfaces. It is characterized by considerable heterogeneity and its spectrum extends from only secretory gland dysfunction to systemic involvement with extra-glandular manifestations in as many as 50% of patients. Thus, symptoms may vary considerably from mild sicca symptoms, pain, fatigue and arthralgia to more severe systemic symptoms such as arthritis, vasculitis or glomerulonephritis. Systemic disease manifests chiefly as rheumatologic, neurologic, pulmonary, hematologic, and renal disorders (Kassan et al 2004). Sjögren's syndrome can occur in a primary form (primary Sjögren's syndrome) or in conjunction with an underlying autoimmune condition (secondary Sjögren's syndrome), such as rheumatoid arthritis or systemic lupus erythematosus (Patel et al 2014). Sjögren's syndrome is also associated with a significantly increased risk of non-Hodgkin B cell lymphoma (Liang et al 2014).

As for other autoimmune disorders, Sjögren's syndrome is more prevalent in women (incidence 10 to 20 times that of men) and the age of onset is generally between 40-50 years of age. The cause of primary Sjögren's syndrome is unknown. It is rare in children (de Souza et al 2012). It is believed that, in addition to gender (sex hormones), the combination of genetic and environmental factors play a role in the development of the disease. In addition to a strong association with genes encoding several human leukocyte antigen (HLA) molecules, association with several genes involved in both the innate and adaptive immune responses have been identified in primary Sjögren's syndrome (Lessard et al 2013). Viral infection or reactivation of latent viruses have been suggested to be involved in the onset, triggering and/or perpetuation of primary Sjögren's syndrome in genetically-susceptible individuals (Lucchesi et al 2014). Hallmarks of primary Sjögren's syndrome are mononuclear cell infiltration of the lacrimal and salivary glands with mainly CD4 helper T cells, and to a lesser extent B cells and CD8 cytotoxic T cells, increased cytokine production, abnormal B cell hyperactivity and autoantibody production. Evidence of the involvement of the innate immunity, especially the type 1 interferon (IFN) system, has also been well-established (Ambrosi and Wahren-Herlenius 2015).

The mononuclear cell infiltration of the exocrine glands with CD4 T cells and the strong association of primary Sjögren's syndrome with HLA locus highlight the importance of antigen presentation to T cells. Through the production of cytokines and the maintenance of B cell-mediated responses, T cells contribute to tissue damage and systemic manifestations of the disease. Target tissue epithelial cells, through the

secretion of cytokines, are thought to contribute to the local initiation and/or maintenance of the innate and adaptive immune responses and to the formation of ectopic lymphoid structures (Ambrosi and Wahren-Herlenius 2015). It has also been suggested that, by providing auto-antigens, they contribute to the activation of autoreactive B cells and the formation of plasmacytoid dendritic cells-activating immune complexes in the target tissue.

No therapy has been demonstrated to affect the course of the disease. Treatment is generally symptomatic and supportive, that is, by using stimulators and substitutes of tears and saliva secretion (such as cholinergic agents, e.g., cevimeline and pilocarpine [Gottenberg et al 2014]) to manage ocular and oral symptoms, as well as supportive surgical procedures, intensive oral hygiene, and prevention and treatment of oral infections (Mavragani and Moutsopoulos 2010). Non-steroidal anti-inflammatory drugs may be used to treat musculoskeletal symptoms and corticosteroids or immunosuppressive drugs may be prescribed for severe systemic complications. Traditional disease-modifying anti-rheumatic drugs (DMARDs) have limited effects in primary Sjögren's syndrome. Hydroxychloroquine, an immunomodulating agent, is widely used in the treatment of Sjögren's syndrome; however, a recent clinical trial has shown limited efficacy (Gottenberg et al 2014). No biological agent has yet been approved for primary Sjögren's syndrome treatment.

For further details on the background of Sjögren's Syndrome, please see Section 2.1 of the Investigator's Brochure (IB).

1.2 BACKGROUND ON CATHEPSIN S

Cathepsin S is a cysteine protease of the papain family that is found within the lysosomes/endosomes of cells, but is also secreted (Gupta et al 2008). It is expressed in the spleen, lymph, heart and lung but, the main expression in normal tissue is in antigen-presenting cells (APCs), including macrophages, B cells and dendritic cells (Hsing and Rudensky 2005). Within APCs, cathepsin S has an integral role in major histocompatibility complex class II (MHC-II) antigen peptide complex formation during antigen presentation (Gupta et al 2008; Hsing and Rudsensky 2005). CD74 (also known as the MHC-II invariant chain or Ii) acts as a chaperone during MHC-II maturation and is degraded in several steps; cleavage of a 10 kDa intermediate fragment ("p10") is catalyzed by cathepsin S. Inhibition of cathepsin S activity leads to accumulation of p10, reduced expression of MHC-II at the cell surface, and potentially results in reduced antigen-presenting capacity. Additionally, cathepsin S also catalyzes degradation of all major components of the extracellular matrix (collagen, elastin, proteoglycan). It is hypothesized that cathepsin S participates in extracellular matrix remodeling (Gupta et al 2008).

Cathepsin S is thought to be involved in a number of autoimmune and allergic conditions, and diseases involving inflammation and matrix remodeling. Cathepsin S is highly up-regulated in injured tissue of patients with auto-immune and metabolic diseases.

Cathepsin S-deficient mice have lower susceptibility to a number of experimentally-induced disease conditions and cathepsin S inhibitors have been shown to be effective in a variety of animal models of autoimmunity and/or local tissue destruction. Therefore, inhibition of cathepsin S is believed to be a possible therapeutic approach for treatment of a number of diseases, including autoimmune diseases such as celiac disease, lupus nephritis and Sjögren’s syndrome.

1.3 BACKGROUND ON RO5459072

RO5459072 is a covalent, reversible, selective and potent competitive inhibitor of cathepsin S being developed for the treatment of Sjögren’s syndrome. The inhibition of cathepsin S by RO5459072 is expected to reduce MHC class II-mediated antigen presentation, attenuate the activation of CD4+ T cells, suppress T cell-dependent auto-antibody production, and neutralize the tissue damage caused by activated macrophages and neutrophils. RO5459072 may therefore represent an important new targeted therapy in Sjögren’s syndrome and other autoimmune diseases.

1.3.1 Previous Non-Clinical Studies

For more detailed information, please refer to the Non-clinical Studies section (Section 4) of the IB.

1.3.1.1 Non-Clinical Pharmacology

In human primary B cells, increasing concentrations of RO5459072 resulted in dose-dependent accumulation of p10, which is cleaved by cathepsin S and therefore, provides an indirect measure of intracellular cathepsin S inhibition. Inhibition of p10 cleavage interferes with maturation of MHC class II molecules in APCs. In human peripheral blood mononuclear cells (PBMCs), RO5459072 reduced the amount of pro-inflammatory cytokines produced (e.g., interleukin-2 [IL-2] and interferon-gamma). In C57/Bl6 mice, increasing concentrations of RO5459072 were shown to dose-dependently increase the levels of the p10 fragment in splenocytes. Similarly, in cynomolgus monkeys, RO5459072 treatment increased concentrations of the p10 fragment in circulating B cells. In a spontaneous mouse model of lupus nephritis (MRL/Faslpr/lpr mice), a reduction in activated T cells, B cells and plasma cells was observed in the spleen. In addition, a significant attenuation in total immunoglobulin (Ig) G1, IgG2a and anti-double stranded DNA (anti-dsDNA) auto-antibody levels was observed after 8 weeks of treatment (by food admix) with 10 mg/kg RO5459072. In a chronic atherosclerosis model in mice, increased accumulation of p10 correlated with decreased plasma levels of pro-inflammatory IL-2 and reduced arterial lipid accumulation.

1.3.1.2 Non-Clinical Pharmacokinetics and Product Metabolism

The pharmacokinetics of RO5459072 were investigated in rats, dogs, and monkeys. In all species, RO5459072 showed a low blood clearance (6-11% of the hepatic blood flow), and a moderate volume of distribution (0.6-1.4 L/kg) and half-life (3.5-4.5 hours).

After oral dosing, RO5459072 reached maximum concentration between 1 and 7 hours (t_{max}). Oral bioavailability was active pharmaceutical ingredient (API)-form and formulation-dependent. Following multiple oral doses of amorphous RO5459072 in rats, exposure increased approximately dose-proportionally from 5 to 100 mg/kg/day. In monkeys, exposure increased in a nearly dose-proportional manner with the crystalline API at doses between 5 and 20 mg/kg/day and less than dose-proportional at higher dose levels. In rabbits, exposure increased in a more than dose-proportional manner with the amorphous API whereas, it increased in a less than dose-proportional manner with the crystalline API. In all species, the compound did not accumulate with dose or time and no sex-specific differences were observed for the exposure.

In human plasma, RO5459072 was moderately bound to plasma proteins (15% free-fraction) and mainly to albumin, and in mammalian species tested the free-fraction was between 12% and 35%. Whole blood distribution occurred mainly to the plasma compartment in animal species, but also to erythrocytes in humans. In vitro metabolism was low to moderate in microsomes and low in hepatocytes across species, consistent with in vivo clearance. In microsomes, the major metabolite observed was M1 (N-demethylation); M2 (oxygenation) was only detected in low amounts in the mouse. In hepatocytes, M1 was the only metabolite observed in all tested species. Furthermore, in vitro cytochrome P450 (CYP)-selective inhibition data showed that M1 was formed via CYP3A4. Urine analysis in rats, dogs, and monkeys revealed that only a small amount of unchanged compound was recovered (≤3.6%), indicating that renal elimination does not play a major role in the clearance of RO5459072 in these species.

RO5459072 did not inhibit the metabolism of CYP1A2-, 2B6-, 2C8-, 2C9-, 2C19-, 2D6- and 3A4/5-mediated reactions up to 50 μM and did not show potential for time-dependent inhibition of CYP3A4. RO5459072 induced CYP3A4, 2B6, and to a lesser extent, 1A2 messenger ribonucleic acid (mRNA) and enzymatic activity in human liver. For CYP3A4 and 2B6, mRNA EC_{50} was 19.2 μM and 39.7 μM, respectively. RO5459072 was found to be a strong P-glycoprotein (P-gp) substrate while also showing good passive membrane permeability. Furthermore, the compound inhibited the transport of substrates of multi-drug resistance protein 1 (MDR1), organic anion-transporting polypeptide (OATP) 1B1, OATP1B3, multidrug and toxin extrusion protein 1 (MATE1) and MATE2-K with IC_{50} values of ≥9.5 μM. However, the transport of substrates of OAT1 and OAT3, and organic cation transporter 2 (OCT2) was not inhibited in the presence of RO5459072.

1.3.1.3 Toxicology and Safety Pharmacology

Oral administration of RO5459072 was well-tolerated in rats and cynomolgus monkeys in repeat-dose toxicity studies up to 13 weeks. Based on the non-clinical safety information available to date, the no-observed-adverse-effect level (NOAEL) following repeated dosing is at least 100 mg/kg/day in rats and 200 mg/kg/day in the cynomolgus monkey, the highest doses tested in 13-week good laboratory practice (GLP) toxicity studies. Mean plasma exposures at the NOAELs were 683 μg•h/mL (AUC_{0-24h}) and

59.20 μg/mL (C_{max}) in the rat, and 99.40 μg • h/mL and 5.44 μg/mL in the cynomolgus monkey for $AUC_{0\text{-}24h}$ and C_{max}, respectively. The no-observed-effect level (NOEL) for cardiovascular effects in the monkey is considered to be at least 600 mg/kg/day, the highest dose tested in the single-dose telemetry study in the monkey.

Target organs of toxicity were only identified at very high exposures during the dose-range finding (DRF) studies, in which the amorphous API form was used. In the rat DRF study, the liver and the heart were identified as possible target organs of toxicity; in the cynomolgus monkey, the liver and the kidney were target organs of toxicity. The heart finding observed in the rat DRF study is considered incidental as it was not observed in the 13-week GLP study at similar exposures. Exposures associated with adverse findings were 815/736 μg • h/mL in male and female rats, and 470/289 μg • h/mL in male and female cynomolgus monkeys, respectively.

Embryo-fetal development (EFD) studies revealed a teratogenic potential of RO5459072 in the rat and a RO5459072-related disturbance in axial skeletal development in both the rat and rabbit. Evidence of teratogenicity, characterized by cardiovascular malformations, was observed in the rat at 7-times the predicted human efficacious $AUC_{(0\text{-}24h)}$. The NOAEL for teratogenicity in the rat was established at 115 mg • h/mL (AUC) and the NOAEL for embryotoxicity was 13 mg • h/mL in the rat and 54 mg • h/mL in the rabbit.

In a single-dose modified Irwin test and a respiratory study, no effects on the central nervous system or respiratory function were observed in rats up to the highest dose tested (100 mg/kg oral).

RO5459072 interacted weakly with human ether-à-go-go-related gene (hERG) channels in vitro (IC_{20} = 41.46 μM or 25 μg/mL). In a single-dose telemetry study in cynomolgus monkeys, cardiovascular parameters were not changed in animals dosed up to 300 mg/kg RO5459072 twice daily (b.i.d.). This dose resulted in a C_{max} of 8.7 μg/mL at 10 hours post-dose.

In vitro and in vivo genotoxicity studies revealed no indication for a mutagenic, clastogenic or aneugenic potential of RO5459072.

1.3.2 Previous Clinical Studies

1.3.2.1 Studies with RO5459072

RO5459072 has been administered to humans in two previously completed studies (Study WP29542 and Study BP29772). A detailed description of the design and results of both studies are presented in the IB (Effects in Humans, Section 5).

Study WP29542 was a single-center, randomized, double-blind, placebo-controlled, single-ascending dose (SAD) study to evaluate the safety, tolerability, pharmacokinetic and pharmacodynamic effects of single oral doses of RO5459072 in healthy male and female volunteers. The study employed an interleaved cohort ("leapfrog") cross-over

design. Subjects were recruited in two cohorts of eight subjects (Cohorts A and B) and dosing alternated between two cohorts, with a minimum of 7 days between each dosing period. Study drug and placebo were administered in a total of eight treatment periods, four periods for each cohort. In each period, subjects received a single oral dose of RO5459072 or placebo, with treatments assigned in a 6:2 ratio, i.e., within each cohort of eight subjects in any period, six subjects received active treatment and two received placebo. Hence, for each individual within a cohort, the study was a randomized, placebo-controlled, four-treatment, four-period, four-way cross-over.

Nine treatments were investigated in the study: single 1, 3, 10, 30, 100, 300, and 600 mg doses of RO5459072 administered while fasted, a single 100 mg dose of RO5459072 administered with a high-fat meal, and placebo. A total of 17 healthy volunteers aged 21-59 years were enrolled in the study. Fifteen subjects completed the study as planned and without major protocol violations.

Study BP29772 was a randomized, double-blind, placebo-controlled, parallel group, ascending-dose design in healthy male and female volunteers with adaptive dose and dosing regimen; the study is clinically completed with the report in progress. The main goal was to investigate the effects of RO5459072 on pharmacodynamic measures of cathepsin S activity and immune function. Other objectives included investigation of the safety and tolerability of one week of dosing with RO5459072 and characterization of the steady-state pharmacokinetics of RO5459072. Four cohorts of nine subjects (Cohorts A to D) were to be recruited, although because of an unexpectedly high screen failure rate only six subjects were recruited in Cohort D. Within each cohort, subjects were assigned to receive active and placebo treatments in a 2:1 ratio, i.e., within each cohort of nine subjects, six subjects received active treatment and three received placebo. After baseline assessments (Day -1), each subject received a single oral dose of RO5459072 or placebo with food (Day 1) followed by the same dose of RO5459072 or placebo once or twice daily (o.d. or b.i.d.) with food for 7 days (Day 3 to Day 9). In addition, each subject received intra-dermal administration of four recall antigens and a negative control at screening and during treatment for assessment of delayed-type hypersensitivity responses. Five treatments were investigated in the study: 50, 100, and 200 mg doses of RO5459072 administered twice daily with food, a 100 mg dose of RO5459072 administered once daily with food, and placebo administered once or twice daily with food. A total of 33 healthy volunteers aged 18-60 years were enrolled in the study. All 33 subjects completed the study as planned and without major protocol violations.

Safety

The incidence of adverse events (AEs) was low in both studies: a total of 77 treatment-emergent AEs were recorded in 15 subjects in Study WP29542 and a total of 54 treatment-emergent AEs were recorded in 20 subjects in Study BP29772. There were no deaths or serious adverse events (SAEs), no withdrawals because of AEs and no dose-limiting AEs were identified. There was no apparent relationship to RO5459072

dose or treatment in the pattern or incidence of AEs. All AEs were considered to be mild in intensity except for five moderate events (influenza in three subjects, vomiting in one subject and muscle strain in one subject) in Study WP29542. Only application site irritation and hypersensitivity (due to ECG electrodes), pollakiuria, headache, disturbance in attention, dizziness, influenza and back pain occurred in more than one subject at any dose of RO5459072 in either study but did not show any apparent relationship with dose.

Mean and mean changes from baseline in vital signs, ECG and safety laboratory parameters showed no apparent dose-related trends or differences compared with placebo.

Pharmacokinetics

RO5459072 pharmacokinetics are both dose- and time-dependent (Figure 1 and Table 1). Exposure was not dose-proportional across dose ranges tested; in particular there was a less-than-dose-proportional increase in exposure at doses above 100 mg (Table 1).

Steady-state was achieved by the third day of dosing. Steady-state exposures were 2-4-fold higher than corresponding single dose exposures, and were ~50% greater than predicted from single-dose data.

Variability in exposure was small and did not appear to change over the dose-range tested (e.g. CV% 17-19% in C_{max} and 12-20% in AUC_{tau}). There were no obvious differences in RO5459072 exposure between men and women or between age groups.

Figure 1 Mean Linear RO5459072 Plasma Concentration versus Time Profiles after a Single Dose and at Steady-State after Twice Daily Administration with Food

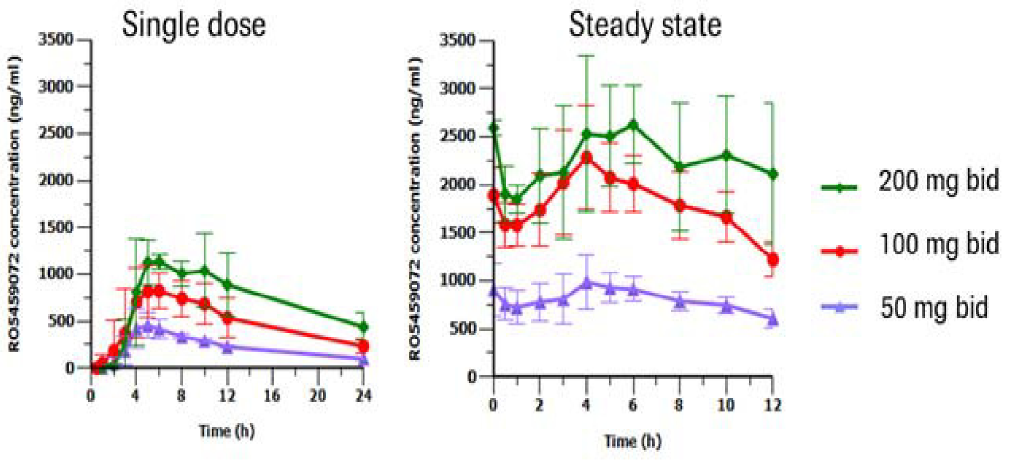

Arithmetic means. Error bars indicate standard deviation.

Table 1 Summary RO5459072 Pharmacokinetic Parameters at Steady-State after Administration with Food

Parameters (Units)	50 mg b.i.d N=6	100 mg b.i.d. N=6	200 mg b.i.d. N=4
$AUC_{0\text{-}12h}$ (ng • h/mL)	9650 (12%)	21500 (14%)	26900 (20%)
C_{max} (ng/mL)	1110 (19%)	2340 (19%)	2950 (18%)
T_{max} (h)	4.5 (0.0-6.0)	4.0 (3.0-6.0)	2.0 (0.0-6.0)

Pharmacokinetic parameters are presented as geometric mean (CV%), except for T_{max} which is shown as median (range).

Dosing with food increased RO5459072 exposure. Average peak and total exposure were 83% and 21% greater, respectively, when a 100 mg dose was administered in fed as compared to fasted state.

Pharmacodynamics

Pharmacodynamic effects were assessed from relative concentrations of 10kDa CD74 intermediate (p10) using a method based on fluorescence-activated cell sorting (FACS) of PBMC preparations. p10 is a substrate for cathepsin S and is therefore, a marker of cathepsin S activity.

Changes in p10 levels in circulating B cells after a single dose (Day 1) and after one week of repeated dosing (Day 9) are shown in Figure 2 .

Figure 2 Mean Change from Baseline in p10 Stimulation Index versus Time Profiles after a Single Oral Dose and after Repeat Dosing with Food

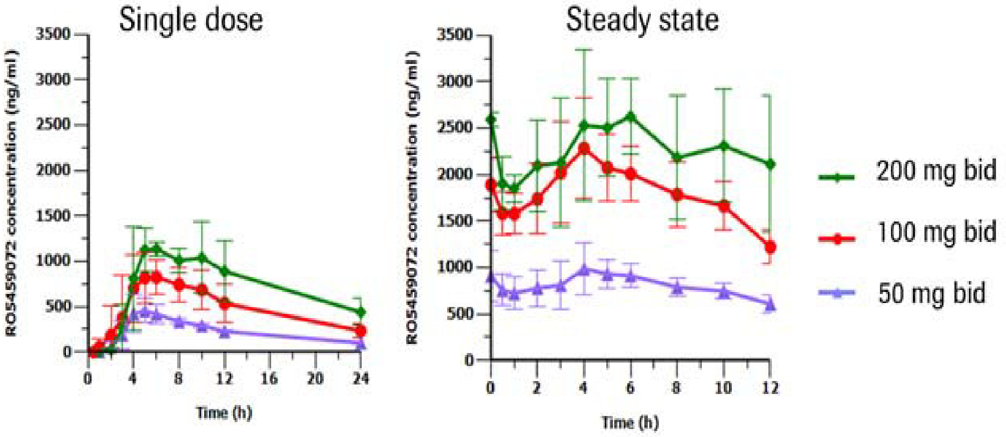

Arithmetic means of change from baseline in p10 after a single oral dose (left graph) and repetitive dosing with food (right graph). Error bars indicate standard deviation.

RO5459072 treatment produced dose-dependent increase in p10 levels in circulating B cells. There were detectable responses to higher doses in all individuals, and

responses were maintained throughout dosing interval after repeat dosing. There was no effect on p10 levels in monocytes.

FACS analysis of PBMCs also indicated that expression of MHC-II (HLA-DR) at the B cell surface was transiently reduced following RO5459072 treatment.

RO5459072 treatment had no apparent effects on delayed type hypersensitivity responses to intradermal recall antigen challenges.

1.3.2.2 Studies with Other Cathepsin S Inhibitors

There is one published report of a clinical study involving dosing of a cathepsin S inhibitor to humans (Payne et al 2014) and the Sponsor has previously conducted a clinical study with another cathepsin S inhibitor structurally related to RO5459072. There were no notable safety findings in either study.

1.4 STUDY RATIONALE AND BENEFIT–RISK ASSESSMENT

1.4.1 Study Rationale

Auto-immune disorders are characterized by an aberrant immunological response to antigens. Sjögren’s syndrome is a chronic systemic autoimmune disease in which an aberrant immune response to self-antigens causes secretory gland dysfunction and systemic symptoms. There is no approved, effective treatment for Sjögren’s syndrome with the exception of products used for managing symptoms of the disorder. Sjögren’s syndrome is characterized by mononuclear cell infiltration of the lacrimal and salivary glands (mainly CD4 helper T cells and to a lesser extent B cells) as well as the presence of circulating autoantibodies. In addition, Sjögren's syndrome is considered a prototypical germinal center auto-immune disorder. Germinal centers in lymphoid tissues are central to B cell development and antibody production in response to antigens, and antigen presentation by B cells to T_{FH} cells plays a significant role in the germinal center reaction. There are multiple lines of evidence that germinal centers are important in Sjögren’s syndrome, including the observation of ectopic "germinal center-like" structures in the salivary glands of approximately 25% of patients. Whilst the exact causes of Sjögren’s syndrome are currently undetermined, together these data indicate the importance of MHC-II antigen presentation as a pathogenic mechanism. Cathepsin S inhibitors have been shown to be effective in non-clinical experimental models of auto-immune disorders including Sjögren’s syndrome (Rupanaqudi et al 2015; Katunuma et al 2003). This study is therefore being conducted with the principal aim of investigating the effects of RO5459072 treatment on disease activity and symptoms of Sjögren’s syndrome. However, because this is an early study in the clinical development of a compound with a novel immunomodulatory mechanism of action, there is a need to profile the pharmacological effects of RO5459072 treatment. The study is, therefore, also intended to characterize the effects of RO5459072 on immune functioning through appraisal of a variety of relevant pharmacodynamic measures and biomarkers.

1.4.2 Benefit-Risk Assessment

Sjögren's syndrome is a chronic systemic auto-immune disease in which an aberrant immune response to self-antigens causes secretory gland dysfunction and systemic symptoms. There is no approved, effective treatment for Sjögren's syndrome. There is, therefore, a reasonable expectation that RO5459072 treatment might ameliorate the signs and symptoms of Sjögren's syndrome. However, this is the first study to test the effects of a cathepsin S inhibitor in patients with Sjögren's syndrome; there are no clinical data to prove that individuals will derive any benefit from RO5459072 treatment. Furthermore, as this is a placebo-controlled study, patients who are randomized to placebo will not derive direct benefit from participation in the study.

The risks of study participation are primarily those associated with adverse reactions to the study drug, although there may also be some discomfort from collection of blood samples and other study procedures. Participants in the optional sub-study will also have additional discomfort and risk of sequelae from the biopsy procedure.

Study BP30037 is the fourth study involving dosing of RO5459072 to humans but the first involving dosing in patients with Sjögren's syndrome. Data from two previously completed clinical studies performed in healthy subjects (Studies WP29542 and BP29772) show a very good tolerability profile over the dose-range tested. There were no notable safety findings either from single doses up to 600 mg or from repeat dosing up to 200 mg b.i.d. administered for one week.

Available data on administration of cathepsin S inhibitors to humans do not indicate any acute safety risks associated with RO5459072 dosing or the mechanism of action. Studies WP29542 and BP29772, and a published report of a single-dose study of a structurally unrelated cathepsin S inhibitor (Payne et al 2014), demonstrate that short-term inhibition of cathepsin S enzyme activity is well-tolerated. Similarly, mice lacking cathepsin S activity (*Ctss* "knockout" mice) have an ostensibly normal phenotype, suggesting that there are no specific risks associated with inhibition of cathepsin S activity. Therefore, repeat-dose non-clinical toxicology studies are the principal guide to potential adverse drug reactions from long-term dosing with RO5459072. Those studies did not reveal any findings of particular concern for dosing patients at the proposed dose of 100 mg b.i.d. RO5459072 up to 12 weeks. The RO5459072 exposures from 100 mg b.i.d. dosing are anticipated to be 2-fold below the corresponding exposures at NOAEL in the cynomolgus monkey and 16-fold below the NOAEL in the rat 13-week study.

The potential for clinically relevant drug-drug interactions between RO5459072 and other concomitant medications is believed to be minimal. Where there is the theoretical potential for pharmacokinetic interactions to occur, specific guidance is given to minimize the potential risk to individuals (e.g., prohibition of potent CYP3A4 inhibitors).

All study participants will undergo safety assessments at intervals throughout the study. Although there were no adverse findings in GLP toxicology studies, effects on the liver

and kidney observed at higher exposures in a non-GLP dose-range finding study in cynomolgus monkeys are considered to be monitorable through a standard battery of laboratory safety tests. The cardiac findings observed in a non-GLP DRF study in rats were not seen in the 13-week GLP toxicity study at similar exposures and are therefore considered incidental. Despite the involvement of cathepsin S in antigen presentation, non-clinical studies and available clinical data reveal no evidence suggesting a risk of clinically important immunosuppression and special measures to monitor immune function are not considered to be necessary. Nevertheless, individuals who are immunosuppressed or are at increased risk of infection will not be enrolled.

Based on the observation that cathepsin S enzyme expression is increased after treatment with a cathepsin S inhibitor, there is a theoretical possibility acute rebound phenomena (i.e., re-emergence of symptoms at a severity worse than at baseline) may occur following discontinuation of RO5459072 treatment. However, because cathepsin S enzyme expression should return to normal soon after discontinuation of RO5459072, any pharmacodynamic rebound should be transient and is considered unlikely to manifest as changes in disease activity or symptoms.

Patients who choose to participate in the optional sub-study will have labial salivary gland biopsies performed at the beginning and end of the study. Labial biopsies are minimally invasive, routinely used for clinical diagnostic purposes (Vitali et al 2002) and have potential utility as an outcome measure in clinical studies (Fisher et al 2015). Nevertheless, there is a risk of sequelae from the biopsy procedure: the incidence of permanent neurological damage is estimated to be ~0.17% (Varela Centelles et al 2014). Therefore, biopsies will only be performed on consenting individuals at selected study centers with experience of performing biopsies, i.e., centers where biopsies are routinely performed as part of normal clinical practice by experienced practitioners.

In summary, non-clinical data provide the necessary rationale that RO5459072 may provide a treatment benefit for patients with Sjögren’s syndrome. No significant safety concerns have been identified about the use of RO5459072 in patients with Sjögren’s syndrome. Where theoretical risks from RO5459072 treatment have been identified from non-clinical studies, appropriate clinical safety monitoring measures are implemented. Hence, the risks to participants in this study are considered acceptable.

2. OBJECTIVES

2.1 PRIMARY OBJECTIVES

The primary objective of this study is:

- To investigate the effects of RO5459072 treatment on disease activity and symptoms of primary Sjögren’s syndrome.

2.2 SECONDARY OBJECTIVES

To investigate the effects of RO5459072 treatment on:

- quality of life measures.
- auto-antibody concentrations.
- pharmacodynamic measures of exocrine gland function.

Additionally:

- To collect samples for population modelling of RO5459072 pharmacokinetics.
- To investigate the safety and tolerability of RO5459072 treatment.

2.3 EXPLORATORY OBJECTIVES

The exploratory objectives for this study are as follows:

To investigate the effects of RO5459072 treatment on:

- cathepsin S enzyme mass and activity.
- sub-populations of circulating white blood cells.
- biomarkers connected to Sjögren's syndrome.
- biomarkers of extracellular matrix degradation.

Additionally:

- To explore the influence of covariates on the effects of RO5459072 treatment.
- To explore alternative composite endpoints as measures for evaluating effects of drug treatment.
- To explore the effects of RO5459072 treatment on the use of concomitant medications.
- To explore the occurrence of acute rebound phenomena on disease activity and symptoms following discontinuation of study drug treatment.

2.4 OPTIONAL SUB-STUDY OBJECTIVE

The objective of the optional sub-study is:

- To investigate the effects of RO5459072 treatment on salivary gland structure, inflammation and the organization of inflammatory foci assessed by histology and immunohistochemistry of labial biopsy samples.

3. STUDY DESIGN

3.1 DESCRIPTION OF STUDY

3.1.1 Overview of Study Design

Study BP30037 is a randomized, double-blind, placebo-controlled, two-treatment arm, parallel-group study designed to evaluate the effects of RO5459072 treatment on disease activity and symptoms of Sjögren's syndrome in adult patients with moderate to severe primary Sjögren's syndrome. Patients will be randomized 1:1 to receive either 100 mg RO5459072 or placebo orally twice daily (b.i.d.) and will be treated for a maximum of 12 weeks. Based on the Investigator judgment, patients may be re-screened and enrolled if the patient meets all of the eligibility criteria at a later time-point.

Assessments of Sjögren's syndrome disease activity, symptoms and quality of life will be made before (baseline [Week -1]), during the study treatment phase, upon completion and, at the early termination visit and at the safety and follow-up visit in order to evaluate the efficacy of RO5459072 treatment. Patients will start study drug treatment one day after the *last* baseline *assessment*. In addition, patients will undergo pharmacodynamic assessments of exocrine gland function and provide samples for measurement of white blood cell numbers and levels of biomarkers associated with Sjögren's syndrome or connected to the mechanism of action of RO5459072, as well as RO5459072 plasma concentrations. Safety and tolerability will be assessed throughout the study.

At selected participating study centers, patients may also enroll into an optional sub-study in which consenting individuals will undergo minor salivary gland (MSG) biopsies for assessing histological changes in labial salivary glands. Biopsies will be taken at baseline (Week -1) and at the end of the study drug treatment period (Week 12, ± 1 week). The data from the sub-study will be analyzed and reported separately.

Patients may concomitantly receive standard of care symptomatic treatment (see Section 4.5.1).

Figure 3 Study Design

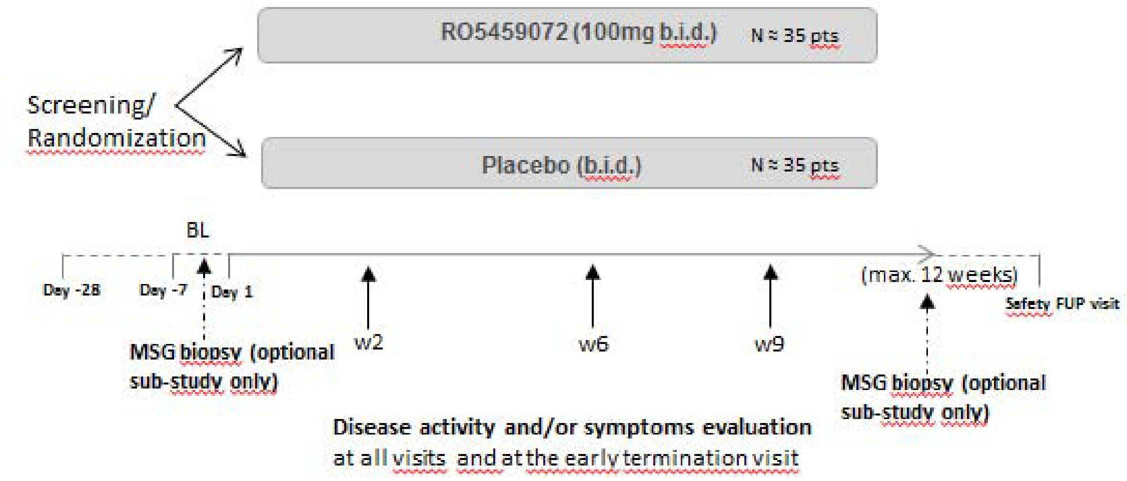

The total duration of the study for each patient will be (up to) 18 weeks:

- Screening visit: Up to 28 days prior to 1st dose.
- Baseline visit (BL): Up to 7 days prior to 1st dose.
- Treatment period: Up to 12 weeks (Days 1 to 84).
- Follow-up visit: 14 days after last dose.

3.1.2 End of Study

The end of the study is defined as the date of the last study center visit of the last patient participating in the study (includes the safety follow-up visit).

3.2 RATIONALE FOR STUDY DESIGN

3.2.1 Rationale for Dosage Selection

A RO5459072 dose of 100 mg b.i.d. has been selected for this study based on pharmacokinetic, pharmacodynamics and safety data from previous clinical studies in healthy volunteers (Studies WP29542 and BP29772; Figure 4).

Figure 4 Simulation of cathepsin S enzyme inhibition over the dosing interval based on population pharmacokinetic/ pharmacodynamic model.

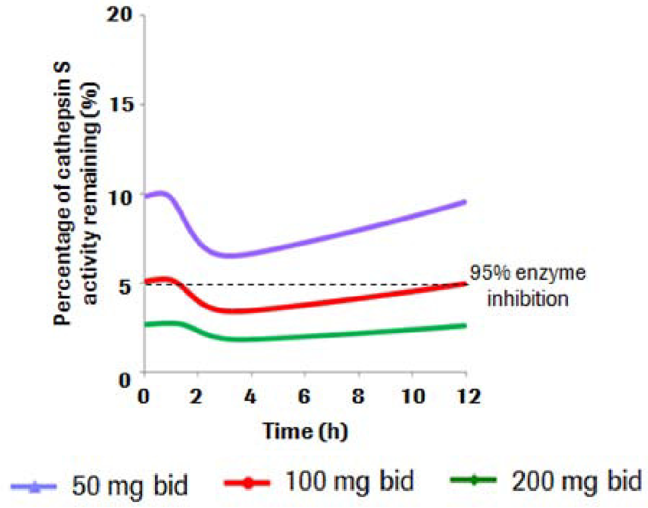

The dose of 100 mg has been well-tolerated when administered as a single-dose under both fasted and fed conditions and in a b.i.d. regimen administered under fed conditions for one week. It also produced robust and sustained effect on a mechanistic marker of intracellular cathepsin S activity (p10 concentrations in circulating B cells). Pharmacokinetic/pharmacodynamic modelling indicate that a 100 mg b.i.d. dose taken with food will maintain ≥95% enzyme inhibition throughout the dosing interval and thereby, maximize the potential for producing functional effects on MHC-II-mediated antigen presentation and immune responses. In turn, this is anticipated to maximize the likelihood to produce treatment benefit on the signs and symptoms of Sjögren's syndrome in a 12-week “proof-of-concept” study. This approach is supported by data from the use of cathepsin S inhibitors in non-clinical experimental models: maximal effects on relevant endpoints are seen at doses which achieve maximal inhibition of intracellular enzyme activity (Investigator's Brochure).

A 100 mg b.i.d. dosing regimen is predicted to result in exposures lower than those observed NOAEL doses in non-clinical toxicology studies. The average steady-state exposures observed at 100 mg b.i.d. dosing in Study BP29772 (2.34 μg/mL and 21.48 μg•h/mL for C_{max} and $AUC_{0\text{-}12h}$, respectively) is 2.3-fold lower than the corresponding exposure at the NOAEL in the most sensitive species in 13-week GLP toxicology studies (cynomolgus monkeys: 5.44 μg/mL and 99.40 μg•h/mL for C_{max} and $AUC_{0\text{-}24h}$, respectively) based on $AUC_{0\text{-}24h}$.

3.2.2 Rationale for Study Population

This trial will enroll adult patients with primary Sjögren's syndrome who present with moderate to severe disease activity and symptoms. This patient population is considered potentially most likely to receive benefit from study drug treatment (Carrubi et al 2013).

Study entry requires a diagnosis of primary Sjögren's syndrome according to the American-European Consensus Group (AECG) diagnostic criteria (Vitali et al 2002); these internationally-recognized consensus criteria based on a combination of subjective and objective measures, are currently considered to be the most appropriate means by which to diagnose Sjögren's syndrome. However, because one of the study aims is to characterize the treatment effects on immune processes, patients are specifically required to have detectable circulating concentrations of anti-Sjögren's-syndrome-related antigen A (anti-SSA) or anti-SSB antibodies. These auto-antibodies are common among patients with Sjögren's syndrome and are considered as a surrogate marker of ongoing germinal center reactions.

It is also required that disease activity and symptoms are considered moderate to severe at the time of enrollment, and therefore, the qualifying participant would be considered a suitable candidate for immunomodulatory pharmacotherapy as part of normal clinical management of the disease. Severity is judged using validated consensus indices which are currently considered as "gold standard" instruments for assessing disease activity and the cardinal symptoms of Sjögren's syndrome (European League against Rheumatism [EULAR] Sjögren's Syndrome Disease Activity Index [ESSDAI] and EULAR Sjögren's Syndrome Patient Reported Index [ESSPRI], respectively. See Section 3.2.5).

Patients who are less likely to benefit from treatment, and those with confounding factors that might mask a drug effect, will not be enrolled. Patients for whom dry eye or xerostomia symptoms are secondary manifestations of other autoimmune disorders (i.e., secondary Sjögren's syndrome) or can be attributed to other causes (e.g., previous radiotherapy or concomitant drug use) will be excluded. Fibromyalgia, significant depression or anxiety are also not permissible, while patients who are using, or have recently used, immunotherapy or immunosuppressive therapy will also not be enrolled. Similarly, although there is no restriction on the length of time a participant has had Sjögren's syndrome, the study will not enroll patients who have no residual salivary gland function because of the expectation that profound salivary gland damage will not be modifiable by drug treatment.

Finally, individuals who may theoretically be placed at additional risk by participating in the study will be excluded. Specific examples are patients with a history of lymphoma, and those who are immunosuppressed or otherwise predisposed to serious infection.

Overall, the entry criteria are consistent with other recently completed and ongoing clinical studies of pharmacological therapies in this patient population (Brown et al 2014).

3.2.3 Rationale for Control Group

Study BP30037 is a placebo-controlled study, and patients will be allocated to RO5459072 and placebo treatment groups in a 1:1 ratio. Study drug treatment will be given on top of the existing symptomatic treatments for Sjögren's syndrome, i.e., patients will be able to continue to use their existing medications. The use of a

placebo comparator is justified because there are currently no approved, effective treatments for Sjögren's syndrome. Furthermore, allocation of equal numbers of patients to the active and placebo groups is considered necessary because of the relative paucity of clinical trial or other longitudinal data available in the public domain for this indication; there is a need to generate an adequate within-study comparator dataset to allow proper evaluation of the magnitude of any treatment effects.

3.2.4 Rationale for Design

The study will employ a parallel group design in which patients will receive either RO5459072 or placebo treatment for 12 weeks. Although maximal inhibition of cathepsin S enzyme activity should occur during the first days of dosing, it is anticipated that any effects on functional pharmacodynamic parameters and clinical endpoints will take weeks or months to become manifest. Similar studies with immunosuppressant compounds (e.g., abatacept, rituximab) suggest that while drug effects may become apparent within 4 to 6 weeks of the start of treatment, full effects only become apparent with longer treatment (Meiners et al 2014, Devauchelle-Pensec et al 2014, Carubbi et al 2013). The maximum duration of study drug treatment is, however, limited by the duration of completed GLP toxicology studies (i.e., 13 weeks).

3.2.5 Rationale for Efficacy Assessments

The primary efficacy outcome for this study is based on assessment of changes in disease activity using the ESSDAI instrument. The ESSDAI is a physician-assessed disease activity index for primary Sjögren's syndrome developed by the EULAR consortium from a worldwide, multicenter study (Seror et al 2010). The ESSDAI has been validated and shows good correlation with a “gold standard” physician global assessment (Seror et al 2015), and has been used in many recent clinical studies (Vissink et al 2012). A score ≥ 5 is considered to represent moderate or severe disease activity and a clinically relevant change in ESSDAI score is defined as an absolute decrease of ≥ 3-points (Seror et al 2014). Disease activity and symptoms are independent aspects of Sjögren's syndrome, and the ESSPRI and ESSDAI scores do not correlate (Seror et al 2013). The ESSDAI has been selected as primary measure because it is a physician-rated index and potentially a more objective and sensitive measure of drug treatment effects.

Secondary efficacy endpoints include assessment of changes in core symptoms of Sjögren's syndrome using the ESSPRI instrument. The ESSPRI is a patient-reported, subjective symptom index for primary Sjögren's syndrome developed by the EULAR consortium from a worldwide, multicenter study (Seror et al 2011). It has also been validated and shows good correlation with a “gold standard” patient global assessment (Seror et al 2015). A score ≥ 5 is considered to represent moderate or severe symptoms and a clinically relevant change in ESSPRI score is defined as an absolute decrease of ≥1-point (Seror et al 2014).

In addition to the disease activity and symptoms specific to Sjögren's syndrome, overall quality of life will be assessed using the SF-36 questionnaire. The SF-36 is a questionnaire developed to evaluate health-related quality of life in individuals with medical disorders (Ware et al 1993). The SF-36 is widely used and validated in a number of different populations and there are existing literature data on use in Sjögren's syndrome as well as other auto-immune disorders: patients with primary Sjögren's syndrome score appreciably worse than matched controls on all domains (Segal et al 2009).

Efficacy and pharmacodynamic assessments will be made at baseline and intervals during the treatment period, thereby allowing changes from baseline in each individual and the speed of onset of any treatment effect to be evaluated. In addition, efficacy assessments will also be made at the follow-up visit in order to explore the occurrence of acute rebound phenomena following discontinuation of study drug treatment. Rebound phenomena (i.e., re-emergence of symptoms at a severity worse than at baseline) are a theoretical possibility based on the observation that cathepsin S enzyme expression is increased after treatment with a cathepsin S inhibitor (Payne et al 2014).

3.2.6 Rationale for Pharmacodynamic Assessments

A range of pharmacodynamic assessments are included in the study in order to profile treatment effects on exocrine gland function, immune function, and other biomarkers connected to Sjögren's syndrome. Impaired tear and saliva production is characteristic of Sjögren's syndrome and production rates will be measured using simple, well-precedented and non-invasive methods. Elevated circulating titers of a range of auto-antibodies are reported in patients with primary Sjögren's syndrome (Tzioufas et al 2012), and as no single type is considered diagnostic, prognostic or predictive a panel including a representative cross-section will be measured, as well as total IgG, IgM and cryoglobulins (Quartuccio et al 2014). FACS immunophenotyping of cell surface markers will be used to determine numbers of cells in defined white blood cell sub-populations of interest and may include TBNK/monocytes, T helper cells and T follicular helper cells and B cells (Szabo et al 2013; Szabo et al 2016). Immunophenotyping will focus on those sub-populations which are reported to be altered in patients with Sjögren's syndrome or which might be expected to change with treatment based on the mechanism of action of RO5459072. Biomarkers to be measured will include erythrocyte sedimentation test (ESR), complement components and β2-microglobulin, which are all reported to be altered in patients with Sjögren's syndrome (Pertovaara and Korpela 2014), along with potentially relevant cytokines (e.g., BAFF, IL-4, IL-6 and IL-10; Pollard et al 2013). Gene expression changes related to interferon response including but not limited to MxA will be measured (Maria et al, 2014).

In addition, markers directly linked to the mechanism of action of RO5459072 will also be measured to quantify the primary and secondary pharmacological effects. These will include cathepsin S mass and activity in plasma, tears and saliva (Hamm-Alvarez et al

2014), and desmosine as a marker of extracellular matrix degradation (Schenke-Layland 2008).

3.2.7 Rationale for Pharmacokinetic Assessments

The plasma concentration data will be pooled with data from other studies (WP29542 and BP29772) for population pharmacokinetic and pharmacokinetic/pharmacodynamic modelling.

3.2.8 Rationale for Safety Assessments

The available clinical and non-clinical data do not indicate any particular safety concerns associated with RO5459072 treatment at the dose proposed and do not suggest any risks that would require specific monitoring. Patients will come to the study center for regular ambulatory visits to undergo standard safety assessments alongside other assessments. Adverse events (AEs) will be collected throughout the study and clinical laboratory assessments, vital signs and triplicate 12-lead ECGs will be monitored as part of a typical approach to evaluating safety in early clinical development studies.

Although inhibition of cathepsin S has the potential to reduce antigen-presenting capacity, non-clinical studies did not reveal any evidence of a risk of clinically important immunosuppression. Therefore, while any infections reported will be carefully evaluated, specific tests of immune function are not considered to be warranted.

3.2.9 Rationale for Other Assessments

It has been reported that the effects of cathepsin S deficiency on MHC-II antigen presentation may be influenced by HLA haplotype and therefore, samples will be collected for genotyping of loci encoding HLAs, along with the cathepsin S gene (*CTss*). The data will then be used to explore how genotype and haplotype may influence drug effects.

3.2.10 Rationale for Biopsy Sub-study

The study will include an optional sub-study to collect MSG biopsy samples. MSG biopsies are routinely used for clinical diagnostic purposes (Vitali et al 2002) and have potential utility as an outcome measure in clinical studies (Fisher et al 2015). For example, it has been reported that long-term rituximab treatment reduces ectopic lymphoid structures and germinal center-like structures in MSG biopsies (Carubbi et al 2013). For recruitment reasons, the MSG biopsies are optional and non-participation in the sub-study will not preclude the inclusion of the patient into the main study.

3.3 OUTCOME MEASURES

3.3.1 Efficacy Outcome Measures

Efficacy assessments will include the ESSDAI, ESSPRI, and the SF-36. ESSDAI and ESSPRI will also be measured at screening in order to assess eligibility for the study.

The primary efficacy outcome for this study is:

- Proportion of patients showing a clinically relevant decrease in ESSDAI score from baseline after 12 weeks.

where a clinically relevant decrease in ESSDAI score is defined as a decrease of ≥ 3 points.

Secondary efficacy outcomes for this study are as follows:

- Proportion of individuals showing a clinically relevant decrease in ESSPRI score after 12 weeks where a clinically relevant decrease in ESSPRI score is defined as a decrease of ≥ 1 point.
- Change from baseline in ESSDAI, ESSPRI and SF-36 scores after 12 weeks.
- Change from baseline in each of the individual components of the ESSPRI (dryness, fatigue and pain) after 12 weeks.

where a clinically relevant decrease in ESSPRI score is defined as a decrease of≥ 1 point.

3.3.2 Pharmacodynamic Outcome Measures

The pharmacodynamic outcome measures for this study are as follows:

- Change from baseline in tear flow rate.
- Change from baseline in salivary flow rate.
- Change from baseline in auto-antibody titres.
- Change from baseline in erythrocyte sedimentation test (ESR).
- Change from baseline in cathepsin S mass and activity in plasma, tears and saliva.
- Change from baseline in circulating numbers of defined sub-populations of white blood cells.
- Change from baseline in biomarker concentrations.

3.3.3 Pharmacokinetic Outcome Measures

RO5459072 plasma concentration data will be pooled with data from other studies (Study WP29542 and Study BP29772) for population pharmacokinetic modelling. The modelling results will be reported separately.

3.3.4 Safety Outcome Measures

The safety outcome measures for this study are as follows:

- The incidence and severity of AEs.
- The incidence of SAEs or withdrawals because of AEs.

- The incidence of out-of-reference-range values for vital signs and ECGs, and laboratory tests.
- Change from baseline in vital signs and ECGs, and laboratory tests by visit.

3.3.5 Exploratory Outcome Measures

The exploratory outcomes for this study are as follows:

- Comparisons of the performance of alternative composite endpoints (e.g., Sjögren's response index [SSRI-30]) as predictive markers of drug treatment response.
- Change from Week 12 to follow-up for ESSDAI, ESSPRI and SF-36 scores in each treatment group.
- Comparisons of drug treatment effects between genotypes.
- Patterns of use of selected concomitant medications.

3.3.6 Optional Sub-study Outcome Measures

Patients consenting to also participate in the optional sub-study will undergo MSG biopsies at baseline and Week 12. Biopsy samples will be processed for histological examination.

The outcomes for the optional sub-study are as follows:

- Change from baseline in focus score (number of lymphocytes per 4 mm^2).
- Change from baseline in numbers of defined sub-populations of infiltrating lymphocytes.
- Subjective evaluation of changes from baseline in minor salivary gland (MSG) structure.

Other exploratory analyses may include, but not be limited to, changes in leukocyte and stromal subpopulation numbers and degree of activation.

4. MATERIALS AND METHODS

4.1 CENTERS

This is a multi-center, multi-country study to be conducted in the US and Europe. Additional sites may be included for back-up purposes and may be activated if needed.

Administrative and Contact Information, and List of Investigators are provided separately.

4.2 STUDY POPULATION

Patients with primary Sjögren's Syndrome will be enrolled into this study. Adults between 18 and 75 years old, diagnosed according to AECG criteria, with moderate to severe disease activity and symptoms based on ESSDAI and ESSPRI score can be enrolled in the study. The study will require approximately 70 patients.

Approximately 30 patients will be enrolled in the optional sub-study.

4.2.1 Recruitment Procedures

Patients will be identified for potential recruitment per site-specific recruitment plans.

4.2.2 Inclusion Criteria

Patients must meet all of the following criteria for study entry:

1. Males and females 18 to 75 years of age, inclusive.
2. Patient willing to give written informed consent and to comply with the study procedures and restrictions.
3. Primary Sjögren's syndrome diagnosed previously according to the revised American-European Consensus Group (AECG) criteria.
4. European League against Rheumatism (EULAR) Sjögren's Syndrome Disease Activity Index (ESSDAI) score ≥ 5.
5. EULAR Sjögren’s Syndrome Patient Reported Index (ESSPRI) score ≥ 5.
6. Elevated serum titers at screening of anti-Sjögren's-syndrome-related antigen A (anti-SSA) and/or anti-SSB antibodies at screening.
7. Negative pregnancy test at screening and baseline (women only), and agreement to comply with measures to prevent pregnancy and restrictions on sperm donation (see Section 4.5.5).

4.2.3 Exclusion Criteria

1. A diagnosis of secondary Sjögren's syndrome according to the revised AECG criteria.
2. Severe complications of Sjögren's syndrome, such as vasculitis with renal, neurologic or cardiac involvement; interstitial lung disease and severe myositis,
3. Systemic immunosuppressant therapy (e.g., abatacept, azathioprine, ciclosporin, etanercept, tofacitinib), cyclophosphamide or B cell depleting therapy (e.g., rituximab anti-CD20 therapy) within 6 months prior to the screening visit. Low dose methotrexate treatment (i.e., oral doses ≤ 20 mg weekly) is, however, permitted.
4. Corticosteroid therapy exceeding 7.5 mg prednisone equivalents per day.
5. Mechanically stimulated whole salivary flow rate at baseline of $<$ 0.1 mL/min.
6. A positive test result for hepatitis B (HBV), hepatitis C (HCV), or human immunodeficiency virus (HIV), *or* tuberculosis, or any other active viral, fungal, yeast or bacterial infection at the screening visit.
7. A history of recurring or chronic infections, any other indication of reduced immune function considered to be clinically significant by the Investigator, or any other underlying conditions which may further predispose patients to serious infection.

8. A history of lymphoma, myeloma (monoclonal hypergammaglobulinemia) or monoclonal gammopathy of unknown significance (MGUS), or any other malignancies within the past 5 years (except basal cell or squamous cell carcinoma of the skin that has been cured).
9. A diagnosis of fibromyalgia, or a diagnosis of significant depression or anxiety that in the opinion of the investigator would confound the interpretation of the study results.
10. Severe renal impairment (e.g., estimated glomerular filtration rate < 30 mL/min), moderate or severe hepatic impairment (e.g., Child-Pugh B or C) or other clinically significant hepatic disease (e.g., AST, ALT or GGT > 2x upper limit of normal).
11. Current or a history of severe, progressive or uncontrolled hematologic, gastrointestinal, endocrine, pulmonary, cardiac, or neurologic disease, or any other concomitant disease or condition or any clinically significant finding at screening that could interfere with, or for which the treatment of might interfere with, the conduct of the study, or that would, in the opinion of the Investigator, pose an unacceptable risk to the individual in this study.
12. Participation in an investigational drug or device study within 3 months prior to screening.
13. Inability to comply with the study protocol for any other reason.
14. *Women who are lactating, breastfeeding or planning to nurse.*
15. *Using other prohibited medication (moderate or potent inhibitors of CYP3A4; strong inducers of CYP3A4; strong inhibitors of the transporter P-glycoprotein [P-gp]; sensitive substrates of CYP3A4 with a narrow therapeutic index).*

4.3 METHOD OF TREATMENT ASSIGNMENT AND BLINDING

4.3.1 Treatment Assignment

Subjects will be randomly assigned to RO5459072 or placebo treatment groups in a 1:1 ratio using interactive voice/web-based response system IxRS (IvRS and IwRS).

4.3.2 Blinding

This is a double-blind study. Blinding to study treatment allocation will be achieved through use of matching-placebo capsules. Neither the patient, nor the Investigator, nor the Sponsor will be aware of the treatment allocation. The randomization list will, however, be made available to the staff at bioanalytical laboratories, to the Sponsor personnel responsible for pharmacokinetic and pharmacodynamic bioanalysis and to programmers.

4.3.3 Emergency Unblinding

In the event of an emergency, the treatment code for an individual patient will be readily available to the Investigator and Sponsor through the IxRS. If unblinding is necessary for patient management (in the case of a serious adverse event), the Investigator will be

able to break the treatment code by contacting the IxRS. Treatment codes should not be broken except in emergency situations. If the Investigator wishes to know the identity of the study drug for any other reason, he or she should contact the Medical Monitor directly. The Investigator should document and provide an explanation for any premature unblinding (e.g., accidental unblinding, unblinding due to a serious adverse event).

As per Health Authority reporting requirements, the Sponsor will break the treatment code for all unexpected serious adverse events (see Section 5.1) that are considered by the Investigator to be related to study drug.

Whenever disclosure of the identity of the study drug is necessary, adequate procedures will be in place to ensure integrity of the data. Any unblinding, at the investigating site end, will be documented in the study report with date, reason for identifying the drug and the name of all the person(s) who had to be unblinded.

4.4 STUDY TREATMENT

4.4.1 Formulation, Packaging, and Handling

4.4.1.1 RO5459072 and Placebo

The investigational medicinal products (IMPs) in this study are:

- RO5459072 50 mg capsules for oral administration (Formulation F03).
- Placebo capsules for oral administration (Formulation F04).

All IMPs will be supplied and packaged by the Sponsor.

Reddish brown, hard gelatin capsules for oral administration will be provided. The capsule fill consists of RO5459072 drug substance only. Matching-placebo capsules are also provided. The capsule fill for the matching-placebo consists only of the inactive ingredient lactose monohydrate of compendial grade.

Study drug packaging will be overseen by the Roche clinical trial supplies department and bear a label with the identification required by local law, the protocol number, drug identification and dosage. Capsules of RO5459072/Placebo should be stored according to the details on the product label ("Do not store above 25°C, protected from light, keep container tightly closed"; see Pharmacy Manual for further details).

The packaging and labeling of the study medication will be in accordance with Roche standard and local regulations.

Upon arrival of investigational products at the site, site personnel should check them for damage and verify proper identity, quantity, integrity of seals and temperature conditions, and report any deviations or product complaints to the monitor upon discovery.

For further details, see the Investigator's Brochure (Section 3.2).

4.4.2 Dosage, Administration, and Compliance

4.4.2.1 RO5459072 and Placebo

Patients will be randomly assigned to one of two possible blinded study drug treatments:

- 100 mg RO5459072 (2 x F03 capsules) b.i.d. with food for 12 weeks.
- Placebo (2 x F04 capsules) b.i.d. with food for 12 weeks.

Study drugs are to be taken orally twice daily (b.i.d.) with food, i.e., with breakfast in the morning and a meal in the evening, for 12 weeks. Patients should start study drug treatment one day after the baseline visit. Capsules should be swallowed whole.

The qualified individual responsible for dispensing the study drug will allocate drug supplies according to the assigned patient randomization number. This individual will write the date dispensed and subject number on the Drug Dispensing Form. This individual will also record the study drug MEDNO received by each subject during the study.

Accountability and subject compliance will be assessed by maintaining adequate study drug dispensing records. The Investigator is responsible for ensuring that dosing is administered in compliance with the protocol. Delegation of this task must be clearly documented and approved by the Investigator.

4.4.3 Investigational Medicinal Product Accountability

All IMPs required for completion of this study (RO5459072 and placebo) will be provided by the Sponsor. The investigational site will acknowledge receipt of IMPs, to confirm the shipment condition and content. Any damaged shipments will be replaced.

The Investigator is responsible for the control of drugs under investigation. Adequate records of the receipt (e.g., Drug Receipt Record) and disposition (e.g., Drug Dispensing Log) of the study drug must be maintained. The Drug Dispensing Log must be kept current and should contain the following information:

- The identification of the patient to whom the study drug was dispensed (for example patient initials and date of birth).
- The date(s), quantity of the study drug dispensed to the patient.
- The date(s) and quantity of the study drug returned by the patient.
- All records and drug supplies must be available for inspection by the Roche Monitor at every monitoring visit.

IMPs will either be disposed of at the study site according to the study site's institutional standard operating procedure or returned to the Sponsor with the appropriate documentation. The site's method of IMP destruction must be agreed upon by the

Sponsor. Local or institutional regulations may require immediate destruction of used investigational medicinal product for safety reasons. In these cases, it may be acceptable for investigational study site staff to destroy dispensed investigational product before a monitoring inspection provided that source document verification is performed on the remaining inventory and reconciled against the documentation of quantity shipped, dispensed, returned, destroyed and provided that adequate storage and integrity of drug has been confirmed.

The site must obtain written authorization from the Sponsor before any IMP is destroyed, and IMP destruction must be documented on the appropriate form.

Written documentation of destruction must contain the following:

- Identity medication number (MEDNO) of investigational product[s] destroyed.
- Quantity of investigational product[s] destroyed.
- Date of destruction.
- Method of destruction.
- Name and signature of responsible person [or company] who destroyed investigational product[s].

Accurate records of all IMPs received at, dispensed from, returned to, and disposed of by the study site should be recorded on the Drug Inventory Log.

4.4.4 Post-Trial Access to RO5459072

RO5459072 will not be provided to participants after conclusion of the study.

4.5 CONCOMITANT THERAPY AND FOOD

4.5.1 Permitted Therapy

In addition to the study drug treatment, patients may continue to concomitantly use existing treatments for Sjögren's syndrome. With exception of the prohibited therapies described in Section 4.5.2, systemic immunomodulatory therapy (e.g., hydroxychloroquine), low dose corticosteroids ($\leq$ 7.5 mg prednisone equivalent dose per day), or low dose methotrexate, or topically administered immunosuppressive agents (e.g., cyclosporine eye drops) are permissible provided the patient has been on a stable dose for $\geq$2 months prior to screening and the dose is expected to remain unchanged during the study. Similarly patients on non-steroidal anti-inflammatory drugs (NSAIDs), antidepressants, or pilocarpine must also have been on a stable dose for 4 weeks prior to screening and be expected to remain on this dose throughout the study. If any of these drugs have been discontinued recently, patients must be off drug for at least 8 weeks before screening.

All medications (prescription and over-the-counter [OTC]) taken within 30 days of study screening visit, until the follow-up visit, will be recorded on the appropriate eCRF. The

dose and/or frequency of use of selected medications commonly prescribed to patients with Sjögren's syndrome will be specifically captured in the eCRF.

4.5.2 Prohibited Therapy

Use of concomitant therapies which systemically suppress the immune system are not permitted during the study. Prohibited therapies are:

- Corticosteroid therapy exceeding 7.5 mg prednisone equivalent per day (see Appendix 3).
- Anti-CD20 therapy (e.g., rituximab) or other B cell-depleting therapy within 6 months of screening.
- Immunosuppressant therapy (e.g., abatacept, azathioprine, ciclosporin, etanercept, tofacitinib). Low dose methotrexate treatment (oral doses $\leq$ 20 mg weekly) is, however, permitted.
- Cyclophosphamide.

Pilocarpine or drugs with similar pharmacological action should not be used within 12 hours prior to an assessment visit.

Because RO5459072 is a substrate of CYP3A4 and P-gp, other prohibited medications are:

- Moderate or potent inhibitors of CYP3A4 (e.g., itraconazole, erythromycin, nefazodone).
- Strong inducers of CYP3A4 (e.g., rifampicin, carbamazepine).
- *Sensitive substrates of CYP3A4 with a narrow therapeutic index (e.g., alfentanil, colchicine, ethosuxamide, fentanyl, and pimozide).*
- Strong inhibitors of the transporter P-glycoprotein (P-gp) (e.g., quinidine, clarithromycin).

A full list of prohibited concomitant medications is provided in Appendix 4.

RO5459072 weakly induces CYP3A4 activity in vitro. RO5459072 treatment therefore has the theoretical potential to cause a small decrease in exposure of concomitantly administered CYP3A4 substrates. For this reason, caution is advised when initiating study drug treatment in individuals already established on a stable dose of a CYP3A4 substrate, or starting dosing with a CYP3A4 substrate in individuals receiving study drug treatment. See Section 4.2.2 regarding use of hormonal contraception.

In vitro data indicate that RO5459072 weakly inhibits the transport of substrates of MDR1 (P-gp), OATP1B1 and MATE2-K transporters. The potential for RO5459072 to alter the pharmacokinetics of substrates of these transporters (e.g., digoxin, pravastatin, metformin, etc) in vivo cannot be entirely discounted. Therefore, caution is advised when

administering study drug treatment together with medications known to be substrates of these transporters.

RO5459072 was found to induce CYP2B6 expression and enzyme activity in vitro but its M1 metabolite was also found to be a moderate inhibitor of CYP2B6 in vitro. It is therefore possible that RO5459072 treatment may lead to changes in exposure of concomitant medications which are predominantly metabolized by CYP2B6. For this reason, caution is advised when administering RO5459072 treatment together with medications known to be sensitive substrates of CYP2B6 (e.g., efavirenz, bupropion).

4.5.3 Vaccination

Investigators should be aware of the theoretical potential for study drug treatment to impair vaccine efficacy. Investigators should therefore follow local guidelines and standards of care for immunization of individuals receiving immunosuppressant therapies. The use of live vaccines is not recommended.

4.5.4 Food

Study drugs are to be taken with food. Food and beverages containing grapefruit or grapefruit juice should be avoided during the study because of the potential effects on CYP3A4 activity.

4.5.5 Contraception

Embryo-fetal development studies identified a teratogenic potential of RO5459072. Enrolment in the study is therefore contingent on agreement to comply with measures to prevent pregnancy. All women are required to have a negative pregnancy test at screening and baseline and any woman who becomes pregnant should be immediately withdrawn from the study (see Sections 4.7.1 and 5.4.3).

Women of childbearing potential must agree to remain abstinent (refrain from heterosexual intercourse) or use a contraception method that results in a failure rate of $<$ 1% per year, during the treatment period and for at least 28 days after the last dose of study drug.

- Examples of non-hormonal contraceptive methods with a failure rate of $<$ 1% per year include bilateral tubal ligation, male sterilization, and copper intra uterine devices.
- Examples of hormonal contraceptive methods with a failure rate of $<$ 1% per year include established, proper use of hormonal contraceptives that inhibit ovulation, hormone-releasing intra-uterine devices. However, because of the theoretical potential for drug-drug interactions which might impair the efficacy of hormonal contraception, these must be supplemented with a barrier method such as male or female condom with spermicide.

A woman is considered not to be of childbearing potential if she is post-menopausal ($\geq$ 12 months of non−therapy-induced amenorrhea, with no identified cause other than menopause) or has undergone surgical sterilization (removal of ovaries and/or uterus).

Men with female partners of childbearing potential or pregnant female partners, men must remain abstinent (refrain from heterosexual intercourse) or use a condom during the treatment period and for at least 28 days after the last dose of study drug to avoid exposing the embryo. All men must refrain from donating sperm during the treatment period and for at least 28 days after the last dose of study drug.

The reliability of sexual abstinence should be evaluated in relation to the duration of the clinical trial and the preferred and usual lifestyle of the patient. Periodic abstinence (e.g., calendar, ovulation, symptothermal, or post ovulation methods) and withdrawal are not acceptable methods of contraception.

4.6 STUDY ASSESSMENTS

4.6.1 Description of Study Assessments

All examinations listed below will be performed according to the schedule of assessments outlined in Appendix 1. Instruction manuals and supply kits will be provided for all central laboratory assessments.

4.6.1.1 Medical History and Demographic Data

Medical history includes clinically significant diseases, all surgeries, reproductive status, smoking history, use of alcohol and drugs of abuse and all concomitant medications (e.g., prescription drugs, OTCs, herbal or homeopathic remedies, nutritional supplements) used by the patient within 30 days prior to the screening visit.

Demographic data will include age, sex, and self-reported race/ethnicity.

4.6.1.2 Physical Examinations

Any abnormality identified at baseline should be recorded on the General Medical History and Baseline Conditions eCRF.

At subsequent visits (or as clinically indicated), limited, symptom-directed physical examinations should be performed. Changes from baseline abnormalities should be recorded in patient's notes. New or worsened clinically significant abnormalities should be recorded as adverse events on the Adverse Event eCRF.

A complete physical examination should include an evaluation of the head, eyes, ears, nose, throat, neck and lymph nodes, and the cardiovascular, dermatological, musculoskeletal, respiratory, gastrointestinal, genitourinary, and neurological systems.

A physical examination will include careful attention to areas of known and possible malignancy.

4.6.1.3 Disease-Specific Assessments

4.6.1.3.1 ESSDAI

EULAR Sjögren's Syndrome Disease Activity Index (ESSDAI) will be employed to assess disease activity at baseline and during the treatment phase. The ESSDAI is a physician-assessed disease activity index for primary Sjögren's syndrome developed by the EULAR consortium (Seror et al 2010; Seror et al 2015). It consists of 44 items in 12 organ-specific 'domains' contributing to disease activity (constitutional, lymphadenopathy, articular, muscular, cutaneous, glandular, pulmonary, renal, peripheral nervous system [PNS], central nervous system [CNS], hematological, biological). Each domain is assessed for activity level (i.e., no, low, moderate, high) and assigned a numerical score based on pre-determined weighting of each individual domain. An overall score is then calculated as the sum of all individual weighted domain scores.

4.6.1.3.2 ESSPRI

EULAR Sjögren's Syndrome Patient Reported Index (ESSPRI) will be used to assess Sjögren's syndrome symptom severity at baseline and during the treatment phase. The ESSPRI is a patient-reported, subjective symptom index for primary Sjögren's syndrome developed by the EULAR consortium (Seror et al 2011, Seror et al 2015). It consists of three questions covering the cardinal symptoms of Sjögren's syndrome: dryness, fatigue and pain (articular and/or muscular). Each domain scored on scale of 0-10, and an overall score is calculated as the mean of the three individual domains where all domains carry the same weight.

A clinically relevant change in ESSPRI score is defined as an absolute decrease of $\geq$1 point (Seror et al 2014).

4.6.1.3.3 SF-36 V2 (Acute Version)

The Short Form 36 Health Survey (SF-36 V2) will be used to assess health-related quality of life at baseline and at on-treatment visits. The SF-36 consists of 36 questions covering 8 domains (general health, physical functioning, role-functioning physical, bodily pain, social functioning, role-functioning emotional, mental health and vitality), with each domain scored on a scale 0-100 (Ware et al 1993). The acute version has a 1-week recall period, asking patients to consider the past week in responding to questions.

4.6.1.3.4 Salivary and lacrimal gland function

- Mechanically Stimulated Salivary Flow

Stimulated salivary flow will be measured at baseline and at on-treatment visits using a mechanical stimulation method. The test should be performed at a similar time of day at each visit and conducted under standard conditions; patients should refrain from oral hygiene procedures, smoking, eating and drinking for at least 2 hours before the test and be seated in an upright position. Patients will be provided with a piece of neutral wax, paraffin, silicone, unflavored chewing gum, or similar chewable, unflavored, non-absorbent material and instructed to chew for a period of 5 minutes. Saliva will be

collected into a pre-weighed collecting container. The container is then re-weighed to determine the weight of saliva. The stimulated salivary flow rate is calculated assuming a specific gravity of 1 (i.e., 1 mL saliva = 1 g) and expressed in mL per minute. The sample will be used for biomarker measurements. The samples can be frozen (for further details, see the Laboratory Manual).

- Tear production

Un-stimulated tear production rate will be measured from both eyes (without the use of analgesics/ anesthetic drops) at baseline and at on-treatment visits using the Schirmer method. A thin strip of filter paper (Schirmer strip, e.g., 35 x 5 mm) will be placed at the junction of the lateral and middle thirds of the lower eyelid of each eye and the eyes should be gently closed. The strips will be left in place for 5 minutes so that tear fluid is absorbed. The maximum length of wetting along the strip at the end of the test period will be measured and recorded in millimeters. The sample will be used for biomarker assessments too.

4.6.1.4 Laboratory Assessments

Laboratory safety tests shall be collected at visits specified in the Schedule of Assessments (Appendix 1). On each occasion patients must have fasted for at least 4 h before sampling.

Additional blood or urine samples may be taken at the discretion of the Investigator if the results of any test fall outside the reference ranges, or clinical symptoms necessitate additional testing to monitor patient's safety. Where the clinical significance of abnormal lab results is considered uncertain, screening lab tests may be repeated before randomization to confirm eligibility. If there is an alternative explanation for a positive urine or blood test for drugs of abuse, e.g., previous occasional intake of a medication or food containing for example codeine, benzodiazepines or opiates, the test could be repeated to confirm washout.

In the event of unexplained abnormal clinically significant laboratory test values, the tests should be repeated immediately and followed up until they have returned to the normal range and/or an adequate explanation of the abnormality is found. Results of clinical laboratory testing will be recorded on the eCRF or be received as electronically produced laboratory reports submitted directly from the central laboratory. Situations may arise when local laboratories are utilized such as for safety labs. In these cases, reference ranges must be submitted where applicable.

With the exception of the urine pregnancy and ESR tests, which will be locally assessed, the samples for the following laboratory tests will be sent to the central laboratory for analysis:

- **Hematology:** Hemoglobin, hematocrit, platelet count, red blood cell (RBC) count, mean corpuscular volume, absolute reticulocyte count, total and differential

leucocyte (WBC) absolute count (neutrophils, eosinophils, lymphocytes, monocytes and basophils).

- ***Blood*** **chemistry:** Albumin, alkaline phosphatase (ALP), alanine aminotransferase (ALT), aspartate aminotransferase (AST), *bicarbonate,* C-reactive protein (CRP), calcium, chloride, creatinine phosphokinase (CPK), gamma-glutamyl transferase (GGT), glucose, phosphate, potassium, creatinine, sodium, total bilirubin, total protein, troponin, urea*, complement (C3 and C4)*.
- **Coagulation:** activated partial thromboplastin time (aPTT), prothrombin time (PT)
- **Lipids:** High-density lipoprotein-cholesterol (HDL-C), low-density lipoprotein cholesterol (LDL-C), triglycerides, total cholesterol.
- **Urinalysis** A midstream urine specimen will be collected for dipstick analysis of protein, blood, glucose, and pH.
- At Screening only: HIV (specific tests HIV-1 antibody, HIV-1/2 antibody, HIV-2 antibody), hepatitis B surface antigen (HBsAg), total hepatitis B core antibody (HBcAb), hepatitis C virus (HCV) antibody*, tuberculosis testing*.
- **Pregnancy test:** All women (including those who have had a tubal ligation) will have a serum pregnancy test at screening and at baseline. A urine pregnancy test will also be done at baseline prior to randomization. Women of childbearing potential will have serum and urine pregnancy tests at all subsequent visits (see SoA table, Appendix 1).

4.6.1.5 Pharmacokinetic Assessments

Blood samples for measurement of plasma concentrations of RO5459072 using a validated assay (and its metabolites as appropriate, in an exploratory manner) will be collected at visits specified in the SoA (Appendix 1). Samples will be sent to a central laboratory for analysis. The date and time of each sample collection will be recorded in the eCRF.

Plasma concentrations of RO5459072 will be measured by a specific and validated liquid chromatography-tandem mass spectrometry (LC-MS/MS) method. Metabolites may also be measured in exploratory manner using qualified analytical methods.

The plasma concentration data will be pooled with data from other studies (e.g., WP29542 and BP29772) for population pharmacokinetic and pharmacokinetic/pharmacodynamic modelling. The results of that population modelling will be reported separately.

4.6.1.6 Pharmacodynamic Assessments

Samples for the pharmacodynamics assessments will be either locally analyzed, sent to one or several central laboratories or to the Sponsor for analysis.

Pharmacodynamic assessments will include the measurement of tear flow rate using the Schirmer's test, stimulated salivary flow rate, serum auto-antibody titers, ESR, collection

of samples for measurement of circulating numbers of sub-populations of white blood cells, biomarkers connected to Sjögren's syndrome and extracellular matrix degradation Cathepsin S mass and activity will be measured in plasma, tears and saliva samples (please see SoA, Appendix 1). Auto-antibody titers will also be measured at screening in order to assess eligibility for the study.

Based on continuous analysis of the data in this study from the samples collected, samples not considered to be critical for safety may be amended or stopped during study conduct.

Auto-antibodies (anti-SSA/SSB)

Serum will be collected for the determination of anti-SSA/SSB autoantibodies as indicated in the SoA (Appendix 1). These measurements will include anti-SSA, anti-SSB, ANA, and rheumatoid factor, as well as total IgG, *IgM* and cryoglobulins.

Immunophenotyping

Whole blood samples will be collected to quantify white blood cell counts by flow cytometry (CD4+ T cells and follicular T helper cells, B cell subsets and natural killer [NK] cells) at the visits indicated in the SoA (Appendix 1). Sub-populations of white blood cells will be counted by FACS immunophenotyping of cell surface markers and may include TBNK/monocytes, T_H and T_{FH} cells and B cells.

Soluble Biomarker Assessments

Plasma samples - Samples for measurement of biomarkers connected to Sjögren's syndrome and extracellular matrix degradation will be collected at specified visits as indicated in the SoA (Appendix 1). Biomarkers to be measured will include β2-microglobulin, cytokines (including - but not limited to - BAFF, IL-4, IL-6 and IL-10), beta-hydroxycholesterol and desmosine.

For sampling procedures, storage conditions, and shipment instructions, refer to the separate central laboratory documentation.

Cathepsin S Mass and Activity

Plasma samples - Samples for measurement of cathepsin S mass and activity will be collected at specified visits as indicated in the SoA (Appendix 1).

Saliva and tear samples – Cathepsin S mass and activity will be measured in saliva and tear fluids collected as part of pharmacodynamic assessments of salivary and lacrimal gland flow (see Section 4.6.1.3).

Erythrocyte Sedimentation Ratio

Samples for determining the ESR value and inflammation related to Sjögren's syndrome will be collected at specified visits as indicated in the SoA (Appendix 1).

mRNA Sampling

A whole blood sample will be collected for mRNA extraction as indicated in the SoA (Appendix 1). The mRNA may be used to measure gene expression changes related to interferon response including, but not limited, to MxA.

4.6.1.7 Clinical Genotyping

A whole blood sample will be collected for DNA extraction, as indicated in the SoA (Appendix 1). The DNA may be used for genotyping of alleles in genes encoding transporters and drug metabolizing enzymes (e.g., CYP3A4, CYP3A5) and transporters (e.g., MDR1), human leukocyte antigens (i.e., HLA gene family) and cathepsin S (CTSS). The DNA will be destroyed after analysis and the data arising from clinical genotyping will be subject to the confidentiality standards described in Section 8.4.

4.6.1.8 Electrocardiograms

Triplicate 12-lead ECG recordings (i.e., three useful ECGs without artifacts) will be obtained within approximately 2-5 minutes at each specified visits. The average of the three readings will be used to determine ECG intervals (e.g., PR, QRS, QT). Additional unscheduled ECGs should be performed in case of abnormalities and if clinical symptoms occur. Whenever possible, the same brand/model of a standard high-quality, high-fidelity electrocardiograph machine equipped with computer-based interval measurements should be used for each patient. The conditions should be as close as possible to the baseline ECG; this includes, but is not limited to, food intake, activity level, stressors and room temperature.

To minimize variability, it is important that patients be in a resting position (semi-recumbent) for ≥10 minutes prior to each ECG evaluation. Body position should be consistently maintained for each ECG evaluation to prevent changes in heart rate. Environmental distractions (e.g., television, radio, conversation) should be avoided during the pre‑ECG resting period and during ECG recording. In some cases, it may be appropriate to repeat abnormal ECGs to rule out improper lead placement as contributing to the ECG abnormality.

For safety monitoring purposes, the Investigator or designee must review, sign, and date all ECG tracings. Paper or electronic copies will be kept as part of the patient's permanent study file at the site. If considered appropriate by Roche, ECGs may be analyzed retrospectively at a central laboratory.

ECG characteristics, including heart rate, QRS duration, and PR, and QT intervals, will be recorded on the eCRF. QTcF (Fridericia's correction) and RR will be calculated by the Sponsor/recorded on the eCRF. Changes in T-wave and U-wave morphology and overall ECG interpretation will be documented on the eCRF. T-wave information will be captured as normal or abnormal, U-wave information will be captured in two categories: absent/normal or abnormal.

4.6.1.9 Vital Signs

Vital signs will include resting blood pressure (BP), heart rate, respiratory rate and body temperature and will be recorded at the visits specified in the Schedule of Assessments (Appendix 1).

Blood pressure, heart rate and respiratory rate should be obtained in a quiet room at a comfortable temperature, with the patient's arm unconstrained by clothing or other material. All measurements will be obtained from the same arm and, with the same cuff size, using a well-calibrated automatic instrument with a digital readout, throughout the study (the "ideal" cuff should have a bladder length that is 80% and a width that is at least 40% of arm circumference [a length-to-width ratio of 2:1]). The automatic cuff should be placed on the designated arm prior to the patient being in a resting position. The patient should be asked to remove all clothing that covers the location of cuff placement. The individual should be comfortably seated, with the legs uncrossed, and the back and arm supported, such that the middle of the cuff on the upper arm is at the level of the right atrium (the mid-point of the sternum). After the patient has been resting in semi-recumbent position for ≥ 10 minutes, blood pressure, heart rate and respiratory rate will be obtained.

4.6.1.10 Administering the SF36 and ESSPRI (Patient-Reported Outcomes)

The SF36 & ESSPRI are PRO instruments which will be completed by patients in this study to more fully characterize the clinical profile of RO5459072. The PRO instruments, translated as required in the local language, will be distributed in paper PRO booklet format by the Investigator staff and completed in their entirety by the patient at specified visits during the study.

Entries should be reviewed for completeness by the site staff during the visit and the patient/caregiver should be requested to complete any blank items. Changes to the form should not be made once the patient/caregiver has left the site for that visit.

The paper PRO booklets are part of the source documentation. A visit-specific paper PRO booklet, containing the PRO assessments for that study day, will be provided. Patients should complete the PRO booklets at the beginning of each study visit, prior to other study assessments or discussion of health status.

4.6.1.11 MSG Biopsies (Optional Sub-study)

At selected participating study centers, patients may also enroll into an optional sub-study in which consenting individuals will undergo labial (MSG) biopsies for assessing histological and structural changes in labial salivary glands using, e.g., immunohistochemistry.

Biopsies will be taken using a minimally invasive technique according to standard practice at each study center, e.g., multiple 2-3mm incisions on the buccal side of the lip

to collect the glandular tissue using forceps. Biopsy sample handling and shipment instructions will be provided in a separate laboratory manual. Histology and immunohistochemistry methods will be described and reported separately.

4.6.1.12 Timing of Assessments - Screening and Pretreatment Assessments

Written informed consent for participation in the study must be obtained before performing any study-specific screening tests or evaluations. Informed Consent Forms for enrolled patient and for patients who are not subsequently enrolled will be maintained at the study site.

All screening and pre-treatment assessments must be completed and reviewed to confirm that patients meet all eligibility criteria. The Investigator will maintain a screening log to record details of all patients screened and to confirm eligibility or record reasons for screening failure. Based on the Investigator judgment, patients may be re-screened and enrolled if the patient meets all of the eligibility criteria at a later time-point.

An Eligibility Screening Form (ESF) documenting the Investigator's assessment of each screened patient with regard to the protocol's inclusion and exclusion criteria is to be completed by the Investigator and kept at the investigational site.

Screening and pre-treatment assessments will be performed within 28 days prior to first dose of study drug treatment, unless otherwise specified. After the study completion/early termination visit, adverse events should be followed as outlined in Sections 5.5, 5.4.3 and 5.6.

For assessments, and timing of assessments, please see the SoA table (Appendix 1).

4.7 PATIENT, STUDY, AND SITE DISCONTINUATION

4.7.1 Patient Discontinuation

The Investigator has the right to discontinue a patient from RO5459072 or withdraw a patient from the study at any time. In addition, patients have the right to voluntarily discontinue study drug or withdraw from the study at any time for any reason. Reasons for discontinuation of study drug or withdrawal from the study may include, but are not limited to, the following:

- Patient withdrawal of consent at any time.
- Pregnancy.
- Any medical condition that the Investigator or Sponsor determines may jeopardize the patient's safety if he or she continues in the study.
- Investigator or Sponsor determines it is in the best interest of the patient.
- The patient requires corticosteroid therapy exceeding 7.5 mg prednisone equivalent per day, or the dose of existing corticosteroid therapy is increased during the study

treatment period (see Section 4.5.2 and Appendix 3), or institution of any other immunosuppressive therapy, or an increase in dose of existing therapy.

Every effort should be made to obtain information on patients who withdraw from the study. The primary reason for withdrawal from the study should be documented on the appropriate eCRF.

Patients will not be followed for any reason after consent has been withdrawn. Patients who withdraw from the study will not be replaced.

Under no circumstances will patients who enroll in this study and have completed treatment as specified, be permitted to be allocated a new randomization number and re-enroll in the study.

4.7.2 Study and Site Discontinuation

The Sponsor has the right to terminate this study at any time. Reasons for terminating the study may include, but are not limited to, the following:

- The incidence or severity of adverse events in this or other studies indicates a potential health hazard to patients.
- Patient enrollment is unsatisfactory.

The Sponsor will notify the investigator and Health Authorities if the study is placed on hold, or if the Sponsor decides to discontinue the study or development program.

The Sponsor has the right to replace a site at any time. Reasons for replacing a site may include, but are not limited to, the following:

- Excessively slow recruitment.
- Poor protocol adherence.
- Inaccurate or incomplete data recording.
- Non-compliance with the International Conference on Harmonisation (ICH) guideline for Good Clinical Practice.

5. ASSESSMENT OF SAFETY

5.1 SAFETY PARAMETERS AND DEFINITIONS

Safety assessments will consist of monitoring and recording adverse events, including serious adverse events and non-serious adverse events of special interest; measurement of protocol‑specified safety laboratory assessments; measurement of protocol-specified vital signs, ECGs; and other protocol-specified tests that are deemed critical to the safety evaluation of the study.

Certain types of events require immediate reporting to the Sponsor, as outlined in Section 5.4

5.1.1 Adverse Events

According to the ICH guideline for Good Clinical Practice, an adverse event is any untoward medical occurrence in a clinical investigation subject administered a pharmaceutical product, regardless of causal attribution. An adverse event can therefore be any of the following:

- Any unfavorable and unintended sign (including an abnormal laboratory finding), symptom, or disease temporally associated with the use of a medicinal product, whether or not considered related to the medicinal product.
- Any new disease or exacerbation of an existing disease (a worsening in the character, frequency, or severity of a known condition).
- Recurrence of an intermittent medical condition (e.g., headache) not present at baseline.
- Any deterioration in a laboratory value or other clinical test (e.g., ECG, X-ray) that is associated with symptoms or leads to a change in study treatment or concomitant treatment or discontinuation from study drug.
- Adverse events that are related to a protocol-mandated intervention, including those that occur prior to assignment of study treatment (e.g., screening invasive procedures such as biopsies).

5.1.2 Serious Adverse Events (Immediately Reportable to the Sponsor)

A serious adverse event is any adverse event that meets any of the following criteria:

- Fatal (i.e., the adverse event actually causes or leads to death).
- Life-threatening (i.e., the adverse event, in the view of the Investigator, places the patient at immediate risk of death).

This does not include any adverse event that had it occurred in a more severe form or was allowed to continue might have caused death.

- Requires or prolongs in-patient hospitalization (see Section 5.3.5.9).
- Results in persistent or significant disability/incapacity (i.e., the adverse event results in substantial disruption of the patient's ability to conduct normal life functions).
- Congenital anomaly/birth defect in a neonate/infant born to a mother exposed to study drug.
- Significant medical event in the Investigator's judgment (e.g., may jeopardize the patient or may require medical/surgical intervention to prevent one of the outcomes listed above).

The terms "severe" and "serious" are not synonymous. Severity refers to the intensity of an adverse event (rated as mild, moderate, or severe, see Section 5.3.3); the event itself

may be of relatively minor medical significance (such as severe headache without any further findings).

Severity and seriousness need to be independently assessed for each adverse event recorded on the eCRF.

Serious adverse events are required to be reported by the investigator to the Sponsor immediately (i.e., no more than 24 hours after learning of the event; see Section 5.4.2 for reporting instructions).

5.1.3 Non-Serious Adverse Events of Special Interest (Immediately Reportable to the Sponsor)

Non-serious adverse events of special interest are required to be reported by the Investigator to the Sponsor immediately (i.e., no more than 24 hours after learning of the event; see Section 5.4.2 for reporting instructions). Adverse events of special interest for this study include the following:

- Cases of elevated transaminases (ALT or AST) in combination with either an elevated bilirubin or clinical jaundice.
- Suspected transmission of an infectious agent by the study drug, as defined below:

  Any organism, virus, or infectious particle (e.g., prion protein transmitting transmissible spongiform encephalopathy), pathogenic or non-pathogenic, is considered an infectious agent. A transmission of an infectious agent may be suspected from clinical symptoms or laboratory findings that indicate an infection in a patient exposed to a medicinal product. This term applies only when a contamination of the study drug is suspected.

5.2 SAFETY PLAN

5.2.1 Management of Specific Adverse Events

No dosage modifications or interruptions are planned for patients participating in this study.

5.3 METHODS AND TIMING FOR CAPTURING AND ASSESSING SAFETY PARAMETERS

The Investigator is responsible for ensuring that all adverse events (see Section 5.1 for definition) are recorded on the Adverse Event eCRF and reported to the Sponsor in accordance with instructions provided in this section and in Sections 5.4-5.6 .

For each adverse event recorded on the Adverse Event eCRF, the Investigator will make an assessment of seriousness (see Section 5.1.2 for seriousness criteria), severity (see Section 5.3.3), and causality (see Section 5.3.4).

5.3.1 Adverse Event Reporting Period

Investigators will seek information on adverse events at each patient contact. All adverse events, whether reported by the patient or noted by study personnel, will be recorded in the patient's medical record. Adverse events will then be reported on the Adverse Event eCRF as follows:

After informed consent has been obtained **but prior to initiation of study drug**, only serious adverse events caused by a protocol-mandated intervention should be reported (e.g., serious adverse events related to invasive procedures such as biopsies). Any other adverse event should not be reported.

After initiation of study drug, all adverse events, regardless of relationship to study drug, will be reported until 28 days after the last dose of study drug. *No additional active monitoring is required after the follow-up visit.*

After a period of 28 days from the last dose of study drug, investigators should report any deaths, serious adverse events, or other adverse events of concern that are believed to be related to prior treatment with study drug (see Section 5.6).

5.3.2 Eliciting Adverse Event Information

A consistent methodology of non-directive questioning should be adopted for eliciting adverse event information at all patient evaluation time-points. Examples of non-directive questions include the following:

"How have you felt since your last clinic visit?"

"Have you had any new or changed health problems since you were last here?"

5.3.3 Assessment of Severity of Adverse Events

Table 2 below provides guidance for assessing adverse event severity.

Table 2 Adverse Event Severity Grading Scale

Severity	Description
Mild	Discomfort noticed, but no disruption of normal daily activity.
Moderate	Discomfort sufficient to reduce or affect normal daily activity.
Severe	Incapacitating with inability to work or to perform normal daily activity.

Note: Regardless of severity, some events may also meet seriousness criteria. Refer to definition of a serious adverse event (see Section 5.1.2).

5.3.4 Assessment of Causality of Adverse Events

Investigators should use their knowledge of the patient, the circumstances surrounding the event, and an evaluation of any potential alternative causes to determine whether or

not an adverse event is considered to be related to the study drug, indicating "yes" or "no" accordingly. The following guidance should be taken into consideration:

- Temporal relationship of event onset to the initiation of study drug.
- Course of the event, considering especially the effects of dose-reduction, discontinuation of study drug, or reintroduction of study drug.
- Known association of the event with the study drug or with similar treatments.
- Known association of the event with the disease under study.
- Presence of risk factors in the patient or use of concomitant medications known to increase the occurrence of the event.
- Presence of non-treatment-related factors that are known to be associated with the occurrence of the event.

5.3.5 Procedures for Recording Adverse Events

Investigators should use correct medical terminology/concepts when recording adverse events on the Adverse Event eCRF. Avoid colloquialisms and abbreviations.

Only one adverse event term should be recorded in the event field on the Adverse Event eCRF.

5.3.5.1 Diagnosis versus Signs and Symptoms

A diagnosis (if known) should be recorded on the Adverse Event eCRF rather than individual signs and symptoms (e.g., record only liver failure or hepatitis rather than jaundice, asterixis, and elevated transaminases). However, if a constellation of signs and/or symptoms cannot be medically characterized as a single diagnosis or syndrome at the time of reporting, each individual event should be recorded on the Adverse Event eCRF. If a diagnosis is subsequently established, all previously reported adverse events based on signs and symptoms should be nullified and replaced by one adverse event report based on the single diagnosis, with a starting date that corresponds to the starting date of the first symptom of the eventual diagnosis.

5.3.5.2 Adverse Events Occurring Secondary to Other Events

In general, adverse events occurring secondary to other events (e.g., cascade events or clinical sequelae) should be identified by their primary cause, with the exception of severe or serious secondary events. However, medically significant adverse events occurring secondary to an initiating event that are separated in time should be recorded as independent events on the Adverse Event eCRF. For example:

- If vomiting results in mild dehydration with no additional treatment in a healthy adult, only vomiting should be reported on the eCRF.
- If vomiting results in severe dehydration, both events should be reported separately on the eCRF.

- If a severe gastrointestinal hemorrhage leads to renal failure, both events should be reported separately on the eCRF.
- If dizziness leads to a fall and subsequent fracture, all three events should be reported separately on the eCRF.

All adverse events should be recorded separately on the Adverse Event eCRF if it is unclear as to whether the events are associated.

5.3.5.3 Persistent or Recurrent Adverse Events

A persistent adverse event is one that extends continuously, without resolution, between patient evaluation time-points. Such events should only be recorded once on the Adverse Event eCRF. The initial severity of the event should be recorded, and the severity should be updated to reflect the most extreme severity any time the event worsens. If the event becomes serious, the Adverse Event eCRF should be updated to reflect this.

A recurrent adverse event is one that resolves between patient evaluation time-points and subsequently recurs. Each recurrence of an adverse event should be recorded separately on the Adverse Event eCRF.

5.3.5.4 Abnormal Laboratory Values

Not every laboratory abnormality qualifies as an adverse event. A laboratory test result should be reported as an adverse event if it meets any of the following criteria:

- Accompanied by clinical symptoms.
- Results in a change in study treatment (e.g., dosage modification, treatment interruption, or treatment discontinuation).
- Results in a medical intervention (e.g., potassium supplementation for hypokalemia) or a change in concomitant therapy.
- Clinically significant in the Investigator's judgment.

It is the Investigator's responsibility to review all laboratory findings. Medical and scientific judgment should be exercised in deciding whether an isolated laboratory abnormality should be classified as an adverse event.

If a clinically significant laboratory abnormality is a sign of a disease or syndrome (e.g., alkaline phosphatase and bilirubin 5-times the upper limit of normal (ULN) associated with cholecystitis), only the diagnosis (i.e., cholecystitis) should be recorded on the Adverse Event eCRF.

If a clinically study significant laboratory abnormality is not a sign of a disease or syndrome, the abnormality itself should be recorded on the Adverse Event eCRF, along with a descriptor indicating if the test result is above or below the normal range (e.g., "elevated potassium", as opposed to "abnormal potassium"). If the laboratory abnormality can be

characterized by a precise clinical term per standard definitions, the clinical term should be recorded as the adverse event. For example, an elevated serum potassium level of 7.0 mEq/L should be recorded as “hyperkalemia”.

Observations of the same clinically significant laboratory abnormality from visit to visit should not be repeatedly recorded on the Adverse Event eCRF, unless the etiology changes. The initial severity of the event should be recorded, and the severity or seriousness should be updated any time the event worsens.

5.3.5.5 Abnormal Vital Sign Values

Not every vital sign abnormality qualifies as an adverse event. A vital sign result should be reported as an adverse event if it meets any of the following criteria:

- Accompanied by clinical symptoms.
- Results in a change in study treatment (e.g., dosage modification, treatment interruption, or treatment discontinuation).
- Results in a medical intervention or a change in concomitant therapy.
- Clinically significant in the Investigator’s judgment.

It is the Investigator’s responsibility to review all vital sign findings. Medical and scientific judgment should be exercised in deciding whether an isolated vital sign abnormality should be classified as an adverse event.

If a clinically significant vital sign abnormality is a sign of a disease or syndrome (e.g., high blood pressure), only the diagnosis (i.e., hypertension) should be recorded on the Adverse Event eCRF.

Observations of the same clinically significant vital sign abnormality from visit to visit should not be repeatedly recorded on the Adverse Event eCRF, unless the etiology changes. The initial severity of the event should be recorded, and the severity or seriousness should be updated any time the event worsens.

5.3.5.6 *Abnormal Liver Function Tests*

The finding of an elevated ALT or AST (> 3 × ULN) in combination with either an elevated total bilirubin (> 2 × ULN) or clinical jaundice in the absence of cholestasis or other causes of hyperbilirubinemia is considered to be an indicator of severe liver injury. Therefore, investigators must report as an adverse event the occurrence of either of the following:

- *Treatment-emergent ALT or AST > 3 × ULN in combination with total bilirubin > 2 × ULN*
- *Treatment-emergent ALT or AST > 3 × ULN in combination with clinical jaundice*

The most appropriate diagnosis or (if a diagnosis cannot be established) the abnormal laboratory values should be recorded on the Adverse Event eCRF (see Section 5.3.5.1) and reported to the Sponsor immediately (i.e., no more than 24 hours after learning of the event), either as a serious adverse event or a non-serious adverse event of special interest (see Section 5.4.2).

5.3.5.7 Deaths

All deaths that occur during the protocol-specified adverse event reporting period (see Section 5.3.1), regardless of relationship to study drug, must be recorded on the Adverse Event eCRF and immediately reported to the Sponsor (see Section 5.4.2).

Death should be considered an outcome and not a distinct event. The event or condition that caused or contributed to the fatal outcome should be recorded as the single medical concept on the Adverse Event eCRF. Generally, only one such event should be reported. The term “sudden death” should only be used for the occurrence of an abrupt and unexpected death due to presumed cardiac causes in a patient with or without preexisting heart disease, within one hour of the onset of acute symptoms or, in the case of an unwitnessed death, within 24 hours after the patient was last seen alive and stable. If the cause of death is unknown and cannot be ascertained at the time of reporting, “unexplained death” should be recorded on the Adverse Event eCRF. If the cause of death later becomes available (e.g., after autopsy), “unexplained death” should be replaced by the established cause of death.

5.3.5.8 Preexisting Medical Conditions

A preexisting medical condition is one that is present at the screening visit for this study. Such conditions should be recorded on the General Medical History and Baseline Conditions eCRF.

A preexisting medical condition should be recorded as an adverse event only if the frequency, severity, or character of the condition worsens during the study. When recording such events on the Adverse Event eCRF, it is important to convey the concept that the preexisting condition has changed by including applicable descriptors (e.g., “more frequent headaches”).

5.3.5.9 Hospitalization or Prolonged Hospitalization

Any adverse event that results in hospitalization or prolonged hospitalization should be documented and reported as a serious adverse event (per the definition of serious adverse event in Section 5.3.5.9), except as outlined below.

The following hospitalization scenarios are <u>not</u> considered to be serious adverse events:

- Hospitalization for respite care.
- Hospitalization for a preexisting condition, provided that all of the following criteria are met:

The hospitalization was planned prior to the study.

The patient has not suffered an adverse event.

The following hospitalization scenarios are not considered to be serious adverse events, but should be reported as adverse events instead:

Hospitalization for an adverse event that would ordinarily have been treated in an outpatient setting had an outpatient clinic been available.

5.3.5.10 Overdoses

Study drug overdose is the accidental or intentional use of the drug in an amount higher than the dose being studied. An overdose or incorrect administration of study drug is not an adverse event unless it results in untoward medical effects.

Any study drug overdose or incorrect administration of study drug should be noted on the Study Drug Administration eCRF.

All adverse events associated with an overdose or incorrect administration of study drug should be recorded on the Adverse Event eCRF. If the associated adverse event fulfills serious criteria, the event should be reported to the Sponsor immediately (i.e., no more than 24 hours after learning of the event; see Section 5.4.2).

5.4 IMMEDIATE REPORTING REQUIREMENTS FROM INVESTIGATOR TO SPONSOR

Certain events require immediate reporting to allow the Sponsor to take appropriate measures to address potential new risks in a clinical trial. The Investigator must report such events to the Sponsor immediately; under no circumstances should reporting take place more than 24 hours after the Investigator learns of the event. The following is a list of events that the Investigator must report to the Sponsor within 24 hours after learning of the event, regardless of relationship to study drug:

- Serious adverse events.
- Non-serious adverse events of special interest.
- Pregnancies.

The Investigator must report new significant follow-up information for these events to the Sponsor immediately (i.e., no more than 24 hours after becoming aware of the information). New significant information includes the following:

- New signs or symptoms or a change in the diagnosis.
- Significant new diagnostic test results.
- Change in causality based on new information.
- Change in the event’s outcome, including recovery.
- Additional narrative information on the clinical course of the event.

Investigators must also comply with local requirements for reporting serious adverse events to the local Health Authority and IRB/EC.

5.4.1 Emergency Medical Contacts

To ensure the safety of study patients, access to the Medical monitors is available 24 hours a day 7 days a week.

Medical monitors contact details are listed in the "Protocol Administrative and Contact Information & List of Investigators".

5.4.2 Reporting Requirements for Serious Adverse Events and Non-Serious Adverse Events of Special Interest

For reports of serious adverse events and non-serious adverse events of special interest (see Sections 5.1.2 and 5.1.3), investigators should record all case details that can be gathered on the Serious Adverse Reporting Form and forward this form to the SAE Responsible within 24 hours.

5.4.3 Reporting Requirements for Pregnancies

5.4.3.1 Pregnancies in Female Patients

Female patients of childbearing potential will be instructed to immediately inform the investigator if they become pregnant during the study or within 28 days after the last dose of study drug. A Clinical Trial Pregnancy Reporting Form should be completed by the Investigator and submitted to the Sponsor within 24 hours after learning of the pregnancy. Pregnancy should not be recorded on the Adverse Event eCRF. The Investigator should discontinue study drug and counsel the patient, discussing the risks of the pregnancy and the possible effects on the fetus. Monitoring of the patient should continue until conclusion of the pregnancy.

5.4.3.2 Pregnancies in Female Partners of Male Patient

Male patients will be instructed through the Informed Consent Form to immediately inform the Investigator if their partner becomes pregnant during the study or within 28 days after the last dose of study drug. A Clinical Trial Pregnancy Reporting Form should be completed by the Investigator and submitted to the Sponsor within 24 hours after learning of the pregnancy. Attempts should be made to collect and report details of the course and outcome of any pregnancy in the partner of a male patient exposed to study drug. The pregnant partner will need to sign an Authorization for Use and Disclosure of Pregnancy Health Information to allow for follow-up on her pregnancy. Once the authorization has been signed, the Investigator will update the Clinical Trial Pregnancy Reporting Form with additional information on the course and outcome of the pregnancy. An Investigator who is contacted by the male patient or his pregnant partner may provide information on the risks of the pregnancy and the possible effects on the fetus, to support an informed decision in cooperation with the treating physician and/or obstetrician.

5.4.3.3 Abortions

Any spontaneous abortion should be classified as a serious adverse event (as the Sponsor considers spontaneous abortions to be medically significant events), recorded on the Adverse Event eCRF, and reported to the Sponsor immediately (i.e., no more than 24 hours after learning of the event; see Section 5.4.2).

Any induced abortion due to maternal toxicity and/or embryo-fetal toxicity should also be classified as serious adverse event, recorded on the Adverse Event eCRF, and reported to the Sponsor immediately (i.e., no more than 24 hours after learning of the event; see Section 5.4.2).

Elective abortion not associated with toxicity (e.g., induced abortion for personal reasons) does not require expedited reporting but should be reported as outcome of pregnancy on the Clinical Trial Pregnancy Reporting Form.

5.4.3.4 Congenital Anomalies/Birth Defects

Any congenital anomaly/birth defect in a child born to a female patient or female partner of a male patient exposed to study drug should be classified as a serious adverse event, recorded on the Adverse Event eCRF, and reported to the Sponsor immediately (i.e., no more than 24 hours after learning of the event; see Section 5.4.2).

5.5 FOLLOW-UP OF PATIENTS AFTER ADVERSE EVENTS

5.5.1 Investigator Follow-Up

The Investigator should follow each adverse event until the event has resolved to baseline grade or better, the event is assessed as stable by the Investigator, the patient is lost to follow-up, or the patient withdraws consent. Every effort should be made to follow all serious adverse events considered to be related to study drug or trial-related procedures until a final outcome can be reported.

During the study period, resolution of adverse events (with dates) should be documented on the Adverse Event eCRF and in the patient's medical record to facilitate source data verification. If, after follow-up, return to baseline status or stabilization cannot be established, an explanation should be recorded on the Adverse Event eCRF.

All pregnancies reported during the study should be followed until pregnancy outcome and reported according to the instructions provided in Section 5.4.3.

5.5.2 Sponsor Follow-Up

For serious adverse events, non-serious adverse events of special interest, and pregnancies, the Sponsor or a designee may follow up by telephone, fax, electronic mail, and/or a monitoring visit to obtain additional case details and outcome information (e.g., from hospital discharge summaries, consultant reports, autopsy reports) in order to perform an independent medical assessment of the reported case.

5.6 POST-STUDY ADVERSE EVENTS

The Investigator is not required to actively monitor patients for adverse events after the end of the adverse event reporting period (defined as 28 days after the last dose of study drug). However, the Sponsor should be notified if the Investigator becomes aware of any death or any other serious adverse event occurring after the end of the adverse event reporting period (*either by faxing, or by scanning and emailing, the Serious Adverse Event Reporting Form using the fax number or email address provided to the investigators)*, if the event is believed to be related to prior study drug treatment.

5.7 EXPEDITED REPORTING TO HEALTH AUTHORITIES, INVESTIGATORS, INSTITUTIONAL REVIEW BOARDS, AND ETHICS COMMITTEES

The Sponsor will promptly evaluate all serious adverse events and non-serious adverse events of special interest against cumulative product experience to identify and expeditiously communicate possible new safety findings to investigators, IRBs, ECs, and applicable Health Authorities based on applicable legislation.

To determine reporting requirements for single adverse event cases, the Sponsor will assess the expectedness of these events using the following reference document:

- RO5459072 Investigator's Brochure

The Sponsor will compare the severity of each event and the cumulative event frequency reported for the study with the severity and frequency reported in the applicable reference document.

Reporting requirements will also be based on the Investigator's assessment of causality and seriousness, with allowance for upgrading by the Sponsor as needed.

6. STATISTICAL CONSIDERATIONS AND ANALYSIS PLAN

The database will be cleaned and frozen when all patients have completed the study or discontinued. The treatment assignment will then be unblinded and analyses will be performed on all data.

All efficacy outcomes will be analyzed using modified intent-to-treat population (mITT) which is defined as all randomized subjects who received any study medication and have evaluable measurement of the parameter of interest at baseline and at least one post-baseline visit. For all efficacy analyses, the treatment groups will be based on the treatment assigned at randomization.

All safety analyses will include all randomized patients who have received any study drug. For these analyses, patients will be grouped according to the treatment actually received.

There will be no adjustment made for any multiple comparisons. To aid the interpretation from the formal hypothesis testing, two-sided 95% confidence intervals will be provided.

Descriptive summaries will include mean, standard deviation, median and range for continuous variables and counts and percentages for categorical variables.

Details of statistical analyses will be provided in the statistical analysis plan.

6.1 DETERMINATION OF SAMPLE SIZE

A total of 70 patients will be randomized in a 1:1 ratio to RO5459072 or placebo (35 patients per treatment group).

Seventy patients (35 per treatment group) will provide *power in the range of* at least 58 *% to* 80 % to detect a difference in ESSDAI response rates between treatment groups *in the range of 25 % to 33 %* (two-sided Chi-square test assuming alpha of 0.*05* and placebo rate ≤ 25%). *This sample size was determined by practical considerations.* S*eventy* patients are also sufficient to provide *approximately 70*% power to detect a difference of 3 points between treatment arms in change from baseline in ESSDAI score (t-test assuming two-sided alpha 0.*0*5 and standard deviation of 5.0).

Approximately 30 patients will be enrolled into the optional sub-study. It is anticipated that enrollment of a minimum of 30 patients should ensure at least 20 patients (10 per treatment group) complete the study and have biopsy samples taken at both baseline and Week 12*, assuming a maximum drop-out rate of 15%*.

6.2 SUMMARIES OF CONDUCT OF STUDY

Protocol deviations will be listed. The number of *patients* who were randomized, discontinued or completed the study will be summarized by treatment groups.

6.3 ANALYSIS POPULATIONS

6.3.1 Efficacy Analysis Population

All efficacy outcomes will be analyzed according to the mITT principle. The mITT population will comprise of all patients who are randomized, received study drug and have evaluable measurement of the parameter of interest at baseline and at least one post-baseline visit. For all efficacy analyses, the treatment groups will be based on the treatment assigned at randomization.

6.3.2 Pharmacodynamic Analysis Population

All patients who have received at least one dose of the study medication and who have pharmacodynamic data available will be included in pharmacodynamic analyses. For these analyses, patients will primarily be grouped according to the treatment actually received; unless otherwise specified in the statistical analysis plan.

6.3.3 Pharmacokinetic Analysis Population

The population methods used for modelling of plasma concentration data will be described separately.

6.3.4 Safety Analysis Population

All patients who have received at least one dose of the study medication will be included in the safety analysis population. For these analyses, patients will be grouped according to the treatment actually received.

6.4 SUMMARIES OF TREATMENT GROUP COMPARABILITY

Demographic and baseline characteristics such as age, race, gender, ESSPRI score, ESSDAI score, salivary flow rate, etc. will be summarized for all randomized patients by treatment group.

6.5 SAFETY ANALYSES

Safety analysis will be performed for all patients in the safety analysis population. All safety parameters will be listed by patient and summarized in tables. Safety will be determined by AEs, laboratory tests, vital signs, ECGs and physical examinations.

There will be no formal statistical analyses of safety data; data will be presented using appropriate summary statistics and graphical plots as appropriate.

Exposure to study medication will be summarized by total duration of study medication and cumulative dose using descriptive statistics. No statistical testing is planned for safety analyses. Dose modifications, interruptions and the reasons will be presented.

6.5.1 Adverse Events

The original terms recorded on the eCRF by the Investigator for adverse events will be standardized by the Sponsor.

AE data will be reported in listings and presented in frequency tables by MedDRA terms. AEs will be summarized by mapped term and appropriate thesaurus level. If applicable, summaries of AE by seriousness and relationship to study treatment will be presented, as well as summaries of AEs leading to death, premature withdrawal from study treatment.

6.5.2 Clinical Laboratory Test Results

All clinical laboratory data will be stored on the database in the units in which they were reported. Patients' listings and summary statistics at each assessment time will be presented using the International System of Units (SI units; Système International d'Unités). Laboratory data not reported in SI units will be converted to SI units before processing.

Laboratory test values will be presented by individual listings with flagging of values outside the normal ranges. Summary tables of change from baseline over time based on SI (Standard International) units will be displayed. Shifts in grade from baseline to the worst grade observed during treatment will be presented for selected laboratory parameters.

6.5.2.1 Standard Reference Ranges and Transformation of Data

Roche standard reference ranges, rather than the reference ranges of the central lab/Investigator, as applicable, will be used. For most parameters, the measured laboratory test result will be assessed directly using the Roche standard reference range. Certain laboratory parameters will be transformed to Roche's standard reference ranges.

A transformation will be performed on certain laboratory tests that lack sufficiently common procedures and have a wide range of Investigator ranges (if applicable), e.g., enzyme tests that include AST, ALT, and alkaline phosphatase and total bilirubin. Since the standard reference ranges for these parameters have a lower limit of zero, only the upper limits of the ranges will be used in transforming the data.

6.5.2.2 Definition of Laboratory Abnormalities

For all laboratory parameters included, there exists a Roche predefined standard reference range. Laboratory values falling outside this standard reference range will be labeled "H" for high or "L" for low in patient listings of laboratory data.

In addition to the standard reference range, a marked reference range has been predefined by Roche for each laboratory parameter. The marked reference range is broader than the standard reference range. Values falling outside the marked reference range that also represent a defined change from baseline will be considered marked laboratory abnormalities (i.e., potentially clinically relevant). If a baseline value is not available for a patient, the midpoint of the standard reference range will be used as the patient's baseline value for the purposes of determining marked laboratory abnormalities. Marked laboratory abnormalities will be labeled in the patient listings as "HH" for very high or "LL" for very low.

6.5.3 Vital Signs

Vital signs data will be presented by individual listings with flagging of values outside the normal ranges and flagging of marked abnormalities. In addition, tabular summaries will be used, as appropriate.

6.5.4 ECG Data Analysis

ECG data will be presented by individual listings with flagging of values outside the normal ranges and flagging of marked abnormalities. In addition, the 12-lead triplicates will be averaged out. All analyses of QT data will be based on averages of the triplicate

measures at each scheduled time-point. These averages will be derived prior to any further data manipulation. Tabular summaries will be used, as appropriate.

6.5.5 Concomitant Medications

The original terms recorded on the patient's eCRF by the Investigator for concomitant medications will be standardized by the Sponsor by assigning preferred terms.

Concomitant medications will be presented in summary tables and listings. The dose and/or frequency of use of selected medications commonly prescribed to patients with Sjögren's syndrome will also be summarized.

6.6 EFFICACY ANALYSES

6.6.1 Primary Efficacy Endpoint

The primary endpoint is the proportion of patients who have $\geq$3 point reduction from baseline in ESSDAI score after 12 weeks of treatment. The proportion of *patients* who have $\geq$3 point reduction from baseline in ESSDAI score after 12 weeks of treatment will be compared between the two treatment arms using *a* Pearson Chi-square test *(two sided p-values, alpha 0.05). The difference in proportions* and corresponding 95% confidence interval (CI) will be provided. *Patients with missing data at Week 12 will be treated as non-responders in the analysis.*

Sensitivity analyses will be conducted if deemed necessary, including sensitivity analyses *for handling* missing data (e.g.*, completer's* analysis or *last observation carried forward (LOCF) method* for missing observations).

In addition; the proportion of patient who have$\geq$*3 point reduction from baseline in ESSDAI score after 12 weeks of treatment will be analyzed using a logistic regression model, adjusting for baseline ESSDAI score.*

6.6.2 Secondary Efficacy Endpoints

Secondary endpoints that are binary will be analyzed using similar methods used for analyzing the primary endpoint.

Continuous secondary endpoints will be analyzed using a Mixed Model for Repeated Measures (MMRM) approach incorporating all observed data up to 12 weeks of treatment. The MMRM will include the absolute change from baseline as the dependent variable. The fixed effects in the model will include treatment, visit (week), treatment-by-visit (weeks) interaction, along with baseline score as continuous covariates. An unstructured variance-covariance structure will be applied to model the within-patient errors, and the restricted maximum likelihood method will be used for estimating the variance components. The denominator degrees of freedom will be estimated using *the Kenward-Roger* approximation.

The least squares mean of change from baseline for each treatment group together with a 95% CI will be calculated from the MMRM model for each visit. At Week 12, the placebo-corrected difference will be estimated from the same MMRM model and will be used to test the null hypothesis of zero difference.

6.7 PHARMACODYNAMIC ANALYSES

All pharmacodynamic parameters will be presented by listings and descriptive summary statistics separately by treatment group.

Pharmacodynamic variables at each assessment time-point along with corresponding changes from baseline will be listed by individual and summarized using descriptive summary statistics. Descriptive summary statistics will include the calculation of arithmetic means, standard deviation, coefficient variation, median, and range, as appropriate. Graphical plots of individual and mean absolute and change from baseline versus time profiles will also be produced for each treatment.

6.8 PHARMACOKINETIC ANALYSES

The population methods used for modelling of plasma concentration data will be described separately in the modelling report.

6.9 EXPLORATORY ANALYSES

Exploratory analyses will be conducted as deemed appropriate.

6.10 INTERIM ANALYSES

No interim analyses are planned for this study.

7. DATA COLLECTION AND MANAGEMENT

7.1 DATA QUALITY ASSURANCE

The Sponsor will be responsible for data management of this study, including quality checking of the data. Sites will be responsible for data entry into the Electronic Data Capture (EDC) system.

A comprehensive validation check program will verify the data. Discrepancies will be generated automatically in the system at the point of entry or added manually for resolution by the Investigator.

The Sponsor will produce a Data Handling Manual that describes the quality checking to be performed on the data. Laboratory electronic data will be sent directly to the Sponsor, using the Sponsor’s standard procedures to handle and process the electronic transfer of these data.

System backups for data stored by the Sponsor and records retention for the study data will be consistent with the Sponsor’s standard procedures.

The Sponsor will be responsible for data management of this study, including quality checking of the data. Sites will be responsible for data entry into the EDC system. In the event of discrepant data, the CRO will request data clarification from the sites, which the sites will resolve electronically in the EDC system.

7.2 ELECTRONIC CASE REPORT FORMS

Data for this study will be captured via an on line EDC system. The data collected in the source documents is entered onto the study eCRF. An audit trail will maintain a record of initial entries and changes made; reasons for change; time and date of entry; and user name of person authorizing entry or change. For each patient enrolled, an eCRF must be completed and electronically signed by the Principal Investigator or authorized delegate from the study staff. If a patient withdraws from the study, the reason must be noted on the eCRF. If a patient is withdrawn from the study because of a treatment-limiting adverse event, thorough efforts should be made to clearly document the outcome.

The Investigator should ensure the accuracy, completeness and timeliness of the data reported to the Sponsor/CRO in the eCRFs and in all required reports.

eCRFs will be submitted electronically to the Sponsor/CRO and should be handled in accordance with instructions from the Sponsor/CRO.

At the end of the study, the Investigator will receive patient data for his or her site in a readable format on a compact disc that must be kept with the study records. Acknowledgement of receipt of the compact disc is required.

7.3 PATIENT-REPORTED OUTCOME DATA

Data from paper PRO questionnaires will be entered into the EDC system by site staff. Paper PRO questionnaires are source data and should be handled according to Section 7.4.

7.4 SOURCE DATA DOCUMENTATION

Study monitors will perform ongoing source data verification to confirm that critical protocol data (i.e., source data) entered into the eCRFs by authorized site personnel are accurate, complete, and verifiable from source documents.

Source documents (paper or electronic) are those in which patient data are recorded and documented for the first time. They include, but are not limited to, hospital records, clinical and office charts, laboratory notes, memoranda, patient-reported outcomes, evaluation checklists, pharmacy dispensing records, recorded data from automated instruments, copies of transcriptions that are certified after verification as being accurate and complete, microfiche, photographic negatives, microfilm or magnetic media, X-rays, patient files, and records kept at pharmacies, laboratories, and medico-technical departments involved in a clinical trial.

Before study initiation, data to be entered directly into the eCRFs (i.e., no prior written or electronic record of the data) and considered source data must be defined in the Trial Monitoring Plan.

Source documents that are required to verify the validity and completeness of data entered into the eCRFs must not be obliterated or destroyed and must be retained per the policy for retention of records described in Section 7.6.

To facilitate source data verification, the investigators and institutions must provide the Sponsor direct access to applicable source documents and reports for trial-related monitoring, Sponsor audits, and IRB/EC review. The investigational site must also allow inspection by applicable Health Authorities.

7.5 USE OF COMPUTERIZED SYSTEMS

When clinical observations are entered directly into an investigational site's computerized medical record system (i.e., in lieu of original hardcopy records), the electronic record can serve as the source document if the system has been validated in accordance with Health Authority requirements pertaining to computerized systems used in clinical research. An acceptable computerized data collection system allows preservation of the original entry of data. If original data are modified, the system should maintain a viewable audit trail that shows the original data as well as the reason for the change, name of the person making the change, and date of the change.

7.6 RETENTION OF RECORDS

Records and documents pertaining to the conduct of this study and the distribution of IMP, including eCRFs, ePRO data (if applicable), informed consent forms, laboratory test results, and medication inventory records, must be retained by the Principal Investigator for at least 15 years after completion or discontinuation of the study, or for the length of time required by relevant national or local Health Authorities, whichever is longer. After that period of time, the documents may be destroyed, subject to local regulations. No records may be disposed of without the written approval of the Sponsor. Written notification should be provided to the Sponsor prior to transferring any records to another party or moving them to another location.

8. ETHICAL CONSIDERATIONS

8.1 COMPLIANCE WITH LAWS AND REGULATIONS

This study will be conducted in full conformance with the ICH E6 guideline for Good Clinical Practice and the principles of the Declaration of Helsinki, or the laws and regulations of the country in which the research is conducted, whichever affords the greater protection to the individual. The study will comply with the requirements of the ICH E2A guideline (Clinical Safety Data Management: Definitions and Standards for Expedited Reporting). Studies conducted in the United States or under a U.S. Investigational New Drug (IND) application will comply with U.S. FDA regulations and

applicable local, state, and federal laws. Studies conducted in the EU/EEA will comply with the EU Clinical Trial Directive (2001/20/EC).

8.2 INFORMED CONSENT

The Sponsor's sample Informed Consent Form will be provided to each site. If applicable, it will be provided in a certified translation of the local language. The Sponsor or its designee must review and approve any proposed deviations from the Sponsor's sample Informed Consent Forms or any alternate consent forms proposed by the site (collectively, the "Consent Forms") before IRB/EC submission. The final IRB/EC-approved Consent Forms must be provided to the Sponsor for health authority submission purposes according to local requirements.

The Consent Forms must be signed and dated by the patient or the patient's legally authorized representative before his or her participation in the study. The case history or clinical records for each patient shall document the informed consent process and that written informed consent was obtained prior to participation in the study.

The Consent Forms should be revised whenever there are changes to study procedures or when new information becomes available that may affect the willingness of the patient to participate. The final revised IRB/EC-approved Consent Forms must be provided to the Sponsor for Health Authority submission purposes.

Patients must be re-consented to the most current version of the Consent Forms (or to a significant new information/findings addendum in accordance with applicable laws and IRB/EC policy) during their participation in the study. For any updated or revised Consent Forms, the case history or clinical records for each patient shall document the informed consent process and that written informed consent was obtained using the updated/revised Consent Forms for continued participation in the study.

A copy of each signed Consent Form must be provided to the patient or the patient's legally authorized representative. All signed and dated Consent Forms must remain in each patient's study file or in the site file and must be available for verification by study monitors at any time.

For sites in the United States, each Consent Form may also include patient authorization to allow use and disclosure of personal health information in compliance with the U.S. Health Insurance Portability and Accountability Act of 1996 (HIPAA). If the site utilizes a separate Authorization Form for patient authorization for use and disclosure of personal health information under the HIPAA regulations, the review, approval, and other processes outlined above apply except that IRB review and approval may not be required per study site policies.

8.3 INSTITUTIONAL REVIEW BOARD OR ETHICS COMMITTEE

This protocol, the Informed Consent Forms, any information to be given to the patient, and relevant supporting information must be submitted to the IRB/EC by the Principal Investigator and reviewed and approved by the IRB/EC before the study is initiated. In addition, any patient recruitment materials must be approved by the IRB/EC.

The Principal Investigator is responsible for providing written summaries of the status of the study to the IRB/EC annually or more frequently in accordance with the requirements, policies, and procedures established by the IRB/EC. Investigators are also responsible for promptly informing the IRB/EC of any protocol amendments (see Section 9.5).

In addition to the requirements for reporting all adverse events to the Sponsor, investigators must comply with requirements for reporting serious adverse events to the local Health Authority and IRB/EC. Investigators may receive written IND safety reports or other safety-related communications from the Sponsor. Investigators are responsible for ensuring that such reports are reviewed and processed in accordance with Health Authority requirements, and the policies and procedures established by their IRB/EC, and archived in the site's study file.

8.4 CONFIDENTIALITY

The Sponsor maintains confidentiality standards by coding each patient enrolled in the study through assignment of a unique patient identification number. This means that patient names are not included in data sets that are transmitted to any Sponsor location.

Patient medical information obtained by this study is confidential and may only be disclosed to third parties as permitted by the Informed Consent Form (or separate authorization for use and disclosure of personal health information) signed by the patient, unless permitted or required by law.

Medical information may be given to a patient's personal physician or other appropriate medical personnel responsible for the patient's welfare, for treatment purposes.

Data generated by this study must be available for inspection upon request by representatives of the U.S. FDA and other national and local Health Authorities, Sponsor monitors, representatives, and collaborators, and the IRB/EC for each study site, as appropriate.

8.5 FINANCIAL DISCLOSURE

Investigators will provide the Sponsor with sufficient, accurate financial information in accordance with local regulations to allow the Sponsor to submit complete and accurate financial certification or disclosure statements to the appropriate Health Authorities. Investigators are responsible for providing information on financial interests during the course of the study and for one year after completion of the study (i.e., LPLV).

9. STUDY DOCUMENTATION, MONITORING, AND ADMINISTRATION

9.1 STUDY DOCUMENTATION

The Investigator must maintain adequate and accurate records to enable the conduct of the study to be fully documented, including but not limited to the protocol, protocol amendments, ICFs, and documentation of IRB/EC and governmental approval. In addition, at the end of the study, the Investigator will receive the patient data, which includes an audit trail containing a complete record of all changes to data.

Roche shall also submit a Development Update Safety Report (DSUR) once a year to the IRB/EC and Health Authorities according to local regulatory requirements and timelines of each country participating in the study.

It is the understanding of the Sponsor that this protocol (and any modifications) as well as appropriate consent procedures and advertisements, will be reviewed and approved by an IRB/EC. This board must operate in accordance with the current Federal Regulations and/or in accordance with Good Clinical Practice. The Sponsor will be sent a letter or certificate of approval prior to initiation of the study, and also whenever subsequent amendments/modifications are made to the protocol.

9.2 SITE INSPECTIONS

Site visits will be conducted by the Sponsor or an authorized representative for inspection of study data, patients' medical records, and eCRFs. The Investigator will permit national and local Health Authorities, Sponsor monitors, representatives, and collaborators, and the IRBs/ECs to inspect facilities and records relevant to this study.

9.3 ADMINISTRATIVE STRUCTURE

The Sponsor of the trial is F. Hoffmann-La Roche Ltd. The Sponsor is responsible for the study management (monitoring), data management, statistical analysis and medical writing for this clinical study report. A clinical study report will be written and distributed to Health Authorities as required by applicable regulatory requirements.

The protocol will be submitted to country/ institutional ethics committees.

9.4 PUBLICATION OF DATA AND PROTECTION OF TRADE SECRETS

The results of this study may be published or presented at scientific meetings. If this is foreseen, the Investigator agrees to submit all manuscripts or abstracts to the Sponsor prior to submission. This allows the Sponsor to protect proprietary information and to provide comments based on information from other studies that may not yet be available to the Investigator.

The Sponsor will comply with the requirements for publication of study results. In accordance with standard editorial and ethical practice, the Sponsor will generally support publication of multicenter trials only in their entirety and not as individual center data. In this case, a coordinating Investigator will be designated by mutual agreement.

Any formal publication of the study in which contribution of Sponsor personnel exceeded that of conventional monitoring will be considered as a joint publication by the Investigator and the appropriate Sponsor personnel.

Authorship will be determined by mutual agreement and in line with International Committee of Medical Journal Editors authorship requirements.

Any inventions and resulting patents, improvements, and/or know-how originating from the use of data from this study will become and remain the exclusive and unburdened property of the Sponsor, except where agreed otherwise.

9.5 PROTOCOL AMENDMENTS

Any protocol amendments will be prepared by the Sponsor. Protocol amendments will be submitted to the IRB/EC and to regulatory authorities in accordance with local regulatory requirements.

Approval must be obtained from the IRB/EC and regulatory authorities (as locally required) before implementation of any changes, except for changes necessary to eliminate an immediate hazard to patients or any non-substantial changes, as defined by regulatory requirements.

10. REFERENCES

Ambrosi A, Wahren-Herlenius M. Update on the immunobiology of Sjögren's syndrome. Curr Opin Rheumatol. 2015;27:468-475.

Brown S, Navarro Coy N, Pitzalis C, et al. The TRACTISS protocol: a randomised double blind placebo controlled clinical trial of anti-B celltherapy in patients with primary Sjögren's Syndrome. BMC Musculoskelet Disord. 2014;15:21.

Carubbi F, Cipriani P, Marrelli A, et al. Efficacy and safety of rituximab treatment in early primary Sjögren's syndrome: a prospective, multi-center, follow-up study. Arthritis Res Ther. 2013;15(5):R172.

Clinical Study Summary – Study WP29542 – A single-center, randomized, double-blind, placebo-controlled, single-ascending dose study to investigate the pharmacokinetics, pharmacodynamic effects, safety and tolerability of single doses of RO5459072 in healthy volunteers. Roche Report No. 1065113, in preparation.

Clinical Study Summary - Study BP29772 - A single-center, randomized, double-blind, placebo-controlled, ascending dose study to investigate the pharmacokinetics, pharmacodynamic effects, safety and tolerability of repeated dosing of RO5459072 in healthy subjects. Roche Report No. 1067538. October 2016.

de Souza TR, Silva IH, Carvalho AT, et al. Juvenile Sjögren syndrome: distinctive age, unique findings. Pediatr Dent. 2012;34:427-430.

Devauchelle-Pensec V, Mariette X, Jousse-Joulin, et al. Treatment of primary Sjögren syndrome with rituximab: a randomized trial. Ann Intern Med. 2014;160:233-242.

Fisher BA, Brown RM, Bowman SJ, et al. A review of salivary gland histopathology in primary Sjögren's syndrome with a focus on its potential as a clinical trials biomarker. Ann Rheum Dis. 2015;74:1645–1650.

Fox RI, Stern M, Michelson P. Update in Sjögren syndrome. Curr Opin Rheumatol. 2000;12:391-398.

Gottenberg JE, Ravaud P, Puéchal X, et al. Effects of hydroxychloroquine on symptomatic improvement in primary Sjögren syndrome: the JOQUER randomized clinical trial. JAMA. 2014;312:249-258.

Gupta S, Singh RK, Dastidar S, et al. Cysteine cathepsin S as an immunomodulatory target: present and future trends. Expert Opin Ther Targets. 2008;12(3):291-299.

Hamm-Alvarez SF, Janga SR, Edman MC, et al. Tear Cathepsin S as a Candidate Biomarker for Sjögren's Syndrome. Arthritis Rheum. 2014;66:1872-1881.

Hsing LC, Rudensky AY. The lysosomal cysteine proteases in MHC class II antigen presentation. Immunol Rev. 2005;207:229-241.

Investigator's Brochure RO5459072 version 2, September 2015.

Kassan SS, Moutsopoulos HM. Clinical manifestations and early diagnosis of Sjögren’s syndrome. Arch Intern Med. 2004;164:1275–1284.

Katunuma N, Matsunaga Y, Himeno K, et al. Insights into the roles of cathepsins in antigen processing and presentation revealed by specific inhibitors. Biol Chem. 2003;384:883-890.

Lessard CJ, Li H, Adrianto I, et al. Variants at multiple loci implicated in both innate and adaptive immune responses are associated with Sjögren's syndrome. Nat Genet. 2013;45:1284-1292.

Liang Y, Yang Z, Baodong Q. Primary Sjögren’s syndrome and malignancy risk: a systemic review and meta-analysis. Ann Rheum Dis. 2014;73:1151-1156.

Lucchesi D, Pitzalis C, Bombardieri M. EBV and other viruses as triggers of tertiary lymphoid structures in primary Sjögren’s syndrome. Expert Rev Clin Immunol. 2014;10:445–455.

Maria NL, Brkic Z, Waris M, et al. MxA as a clinically applicable biomarker for identifying systemic interferon type I in primary Sjogren's syndrome. Ann Rheum Dis. 2014;73(6):1052-1059.

Mavragani CP, Moutsopoulos HM. The geoepidemiology of Sjögren's syndrome. Autoimmun Rev. 2010;9:A305-A310.

Meiners PM, Vissink A, Kroese FG, et al. Abatacept treatment reduces disease activity in early primary Sjögren’s syndrome (open-label proof of concept ASAP study). Ann Rheum Dis. 2014;73:1393–1396.

Patel R, Shahane A. The epidemiology of Sjögren's syndrome. Clin Epidemiol. 2014;6:247–255.

Payne CD, Deeg MA, Chan M, et al. Pharmakokinetics and pharmacodynamics of cathepsin S inhibitor, LY3000328, in healthy subjects. Br J Clin Pharmacol. 2014;78(6):1334-1342.

Pertovaara M, Korpela M. ESSPRI and other patient-reported indices in patients with primary Sjogren's syndrome during 100 consecutive outpatient visits at one rheumatological clinic. Rheumatol (Oxford). 2014;53(5):927-931.

Pollard RPE, Abdulahad WH, Bootsma H, et al. Predominantly proinflammatory cytokines decrease after B cell depletion therapy in patients with primary Sjögren's syndrome. Ann Rheum Dis. 2013;72:2048-2050.

Quartuccio L, Isola M, Baldini C, et al. Biomarkers of lymphoma in Sjögren’s syndrome and evaluation of the lymphoma risk in prelymphomatous conditions: Results of a multicenter study. J Autoimmunity. 2014;51:75-80.

Rupanaqudi KV, Kulkarni OP, Lichtnekert J, et al. Cathepsin S inhibition suppresses systemic lupus erythematosus and lupus nephritis because cathepsin S is essential for MHC class II-mediated CD4 T-cell and B-cell priming. Ann Rheum Dis. 2015;74:452-463.

Schenke-Layland K, Xie J, Angelis E, et al. Increased degradation of extracellular matrix structures of lacrimal glands implicated in the pathogenesis of Sjögren's syndrome. Matrix Biol. 2008;27:53-66.

Segal B, Bowman SJ, Fox PC et al. Primary Sjögren’s Syndrome: health experiences and predictors of health quality among patients in the United States. Health and Quality of Life Outcomes. 2009;7:46.

Seror R, Ravaud P, Bowman S, et al. EULAR Sjögren’s Syndrome Disease Activity Index (ESSDAI): development of a consensus systemic disease activity index in primary Sjögren’s syndrome. Ann Rheum Dis. 2010;69:1103–1109.

Seror R, Ravaud P, Mariette X, et al. EULAR Sjögren’s Syndrome Patient Reported Index (ESSPRI): development of a consensus patient index for primary Sjögren’s syndrome. Ann Rheum Dis. 2011;70:968–972.

Seror R, et al. European League Against Rheumatism Sjögren's Syndrome Disease Activity Index and European League Against Rheumatism Sjögren's Syndrome Patient-Reported Index: a complete picture of primary Sjögren's syndrome patients. Arthritis Care Res. 2013;65(8):1358-1364.

Seror R, Bootsma H, Saraux A, et al. Defining disease activity states and clinically meaningful improvement in primary Sjögren's syndrome with EULAR primary Sjögren's syndrome disease activity (ESSDAI) and patient-reported indexes (ESSPRI). Ann Rheum Dis. 2014;0:1–8.

Seror R, Theander E, Brun JG, et al. Validation of EULAR primary Sjögren's syndrome disease activity (ESSDAI) and patient indexes (ESSPRI). Ann Rheum Dis. 2015;74(5):859-866.

Szabó K, Papp G, Barath S, et al. Follicular helper T cells may play an important role in the severity of primary Sjögren’s syndrome. Clin Immunol. 2013;147:95-104.

Szabó, K, Papp G, Szántó A, et al. A comprehensive investigation on the distribution of circulating follicular T helper cells and B cell subsets in primary Sjögren’s syndrome and systemic lupus erythematosus. Clin Exp Immuno. 2016;183(1):76-89.

Tzioufas AG, Tatouli IP, Moutsopoulos HM. Autoantibodies in Sjögren's syndrome: clinical presentation and regulatory mechanisms. Presse Med. 2012;41(9 Pt 2):e451-460.

Varela Centelles P, Sánchez-Sánchez M, Costa-Bouzas J, et al. Neurological adverse events related to lip biopsy in patients suspicious for Sjögren's syndrome: a systematic review and prevalence meta-analysis. Rheumatol (Oxford). 2014;53(7):1208-1214.

Vissink A, Bootsma H, Kroese FG, et al. How to assess treatment efficacy in Sjögren's syndrome? Curr Opin Rheumatol. 2012;24(3):281-289.

Vitali C, Bombardieri S, Jonsson R, et al. Classification criteria for Sjögren's syndrome: a revised version of the European criteria proposed by the American-European Consensus Group. Ann Rheum Dis. 2002;61(6):554-558.

Ware JE, Snow KK, Kosinski M, et al. SF-36 Health Survey. Manual and interpretation guide. Boston, MA: The Health Institute, New England Medical Center, 1993.

Appendix 1
Schedule of Assessments

			Treatment Period					
**Visit**[8]	**Screening**	**Baseline**[1]	**Week 2**	**Week 6**	**Week 9**	**Week 12**	**Follow-Up Visit**	**Early Termination Visit**
**Days**	-28 to -8	-7 to -1	14 (± 7)	42 (± 7)	63 (± 7)	84 (± 7)	98 (± 7)	(NA)
**Informed Consent**	x							
**Randomization**		x						
**Study drug dispensing**		x	x	x				
**Study drug return**				x		x		x
**Entry criteria**	x	x						
**Demography**	x							
**Medical History**	x							
**Physical Examination**[2]	x						x	x
**Pregnancy testing**[3]	x	x	x	x	x	x	x	x
**Tuberculosis testing**	x							
**HIV, HBV and HCV testing**	x							
**Clinical Genotyping**		x						
**ESSDAI score**	x	x		x		x	x	x
**ESSPRI score**	x	x	x	x	x	x	x	x
**SF-36**		x		x		x		
**Salivary samples**[4]		x	x	x		x		
**Saliva flow assessment**		x	x	x		x		
**Tear samples**[4]		x	x	x		x		
**Tear flow assessment**		x	x	x		x		
**mRNA sample**		x	x	x		x		
**Autoantibodies**	x	x		x		x	x[5]	x[5]
**Soluble biomarkers**		x	x	x		x		
**Immunophenotyping**		x	x	x		x		
**Cathepsin S activity/ mass**		x	x	x		x		
**ESR test**		x	x	x		x		
**PK sample**			x	x		x		
**Safety laboratory assessments**[6]	x	x	x	x	x	x	x	x
**ECG - 12 Lead**	x	x	x	x	x	x	x	x
**Vital Signs**	x	x	x	x	x	x	x	x
**MSG biopsy (optional sub-study only)**		x				x[7]		
**Adverse Event or Intercurrent Illness**					x			
**Previous and Concomitant Treatments**								

Appendix 1
Schedule of Assessments (cont.)

1	First study drug administration should be one day after the *last* baseline *assessment*.
2	Physical examination includes weight, heart and respiratory rate, and body temperature.
3	All women will have serum pregnancy tests performed at screening and baseline. A urine pregnancy test will also be done at baseline prior to randomization. All women of childbearing potential will have serum and urine pregnancy tests at each subsequent visit.
4	The same salivary or tear sample is used for volume and biomarker assessments *(see Section 4.6.1.3.4.)*.
5	*Only markers required for ESSDAI scoring will be collected.*
6	Safety laboratory assessment consists of hematology, blood chemistry, coagulation, lipids and urinalysis.
7	MSG biopsies procedures for the optional substudy may be performed at separate study center visits providing the procedures are carried out within the windows specified for the main study.
8	Screening, baseline and on-treatment visit schedules are all relative to the first day of study drug administration (Day 1). The follow-up visit is scheduled relative to the Week 12 end-of-treatment visit (+14 days after the end-of-treatment visit [± 7 days]).

Appendix 2
Disease-Specific Assessments

ESSDAI

Domains	Weights	No Activity 0	Low Activity 1	Moderate Activity 2	High Activity 3
Constitutional symptoms	3	☐	☐	☐	☐
Lymphadenopathy	4	☐	☐	☐	☐
Glandular swelling	2	☐	☐	☐	☐
Articular	2	☐	☐	☐	☐
Cutaneous	3	☐	☐	☐	☐
Pulmonary	5	☐	☐	☐	☐
Renal	5	☐	☐	☐	☐
Muscular	6	☐	☐	☐	☐
PNS	5	☐	☐	☐	☐
CNS	5	☐	☐	☐	☐
Hematological	2	☐	☐	☐	☐
Biological	1	☐	☐	☐	☐

ESSPRI

1) How severe has your dryness been during the last 2 weeks ?

No dryness	☐ 0	☐ 1	☐ 2	☐ 3	☐ 4	☐ 5	☐ 6	☐ 7	☐ 8	☐ 9	☐ 10	Maximal imaginable dryness

2) How severe has your fatigue been during the last 2 weeks ?

No fatigue	☐ 0	☐ 1	☐ 2	☐ 3	☐ 4	☐ 5	☐ 6	☐ 7	☐ 8	☐ 9	☐ 10	Maximal imaginable fatigue

3) How severe has your pain (joint or muscular pains in your arms or legs) been during the last 2 weeks ?

No pain	☐ 0	☐ 1	☐ 2	☐ 3	☐ 4	☐ 5	☐ 6	☐ 7	☐ 8	☐ 9	☐ 10	Maximal imaginable pain

AECG

16. Primary Sjögren's syndrome diagnosed according to the revised American-European Consensus Group (AECG) criteria i.e. presence of any 4 of the 6 items below, with a least items d and f, or presence of any 3 of items c, d, e and f:

a) Ocular dryness symptoms.

b) Oral dryness symptoms.

c) Schirmer’s test $\leq$5 mm/5min.

d) Focus score $\geq$1 focus/4 mm^2 on minor salivary gland biopsy.

e) Unstimulated whole salivary flow $\leq$0.1 mL/min.

f) Positive anti-SSA or SSB antibodies.

Appendix 3
Dose-Equivalents of Systemic Corticosteroids

	Relative Glucocorticoid Activity	Approximate Equivalent Dose* (mg)
Short-acting		
Hydrocortisone	1	30
Cortisone	0.8	37.5
Intermediate-acting		
**Prednisone**	4	7.5
Prednisolone	4	7.5
Methylprednisolone	5	6
Triamcinolone	5	6
Dexamethasone	30	1
Betamethasone	30	1

*Equivalent dose shown is for oral or IV administration.

Table adapted from:

Liu D, Ahmet A, Ward L, et al. A practical guide to the monitoring and management of the complications of systemic corticosteroid therapy. Allergy Asthma Clin Immunol. 2013;9(1):30.

Appendix 4
Prohibited Concomitant Medications

Corticosteriods for systemic use (ATC H02)

(Note that prednisone or prednisone equivalent of ≤7.5mg is permitted.)

Aldosterone, betamethasone, cloprednol, cortisone, cortivazol, deflazacort, desoxycortone, dexamethasone, fludrocortisone, fluocortolone, hydrocortisone, meprednisone, methylprednisolone, paramethasone, prednisolone, prednisone, prednylidene, rimexolone, triamcinolone, trilostane.

Immunosuppressants (ATC L04A)

(Note that low dose methotrexate ≤ 20 mg is permitted as per Section 4.5.2 , Prohibited Therapy.)

Abatacept, abetimus, adalimumab, afelimomab, alefacept, alemtuzumab, anakinra, anti-lymphocyte immunoglobulin, apremilast, azathioprine, basiliximab, belatacept, belimumab, briakinumab, canakinumab, certolizumab pegol, ciclosporin, daclizumab, eculizumab, efalizumab, etanercept, everolimus, fingolimod, golimumab, gusperimus, infliximab, leflunomide, lenalidomide, mepolizumab, methotrexate, muromonab-CD3, mycophenolic acid, natalizumab, pirfenidone, pomalidomide, rilonacept, secukinumab, siltuximab, sirolimus, tacrolimus, teriflunomide, thalidomide, tocilizumab, tofacitinib, ustekinumab, vedolizumab, voclosporin.

CYP3A4 inducers

Barbiturates, carbamazepine, efavirenz, systemic glucocorticoids, modafinil, nevirapine, oxcarbazepine, phenobarbital, phenytoin, pioglitazone, rifabutin, rifampin, St. John's wort (hyperforin).

CYP3A4 inhibitors

Aprepitant, clarithromycin, diltiazem, erythromycin, fluconazole, indinavir, itraconazole, ketoconazole, nefazodone, nelfinavir, ritonavir, saquinavir, telithromycin, verapamil.

Appendix 4
Prohibited Concomitant Medications (cont.)

MDR1 (P-gp) inhibitors

Amiodarone, azithromycin, carvedilol, clarithromycin, dronedarone, erythromycin, fluvoxamine, indinavir, itraconazole, ketoconazole, lapatinib, propafenone, quercetin, quinidine, quinine, ranolazine, ritonavir (and ritonavir combinations), telaprevir, verapamil, vorapaxar.

Sensitive substrates of CYP3A4 with a narrow therapeutic index

Alfentanil, colchicine,ethosuxamide, fentanyl, pimozide.

AMENDMENT HISTORY FOR PROTOCOL BP30037, VERSION 3

RATIONALE

Protocol BP30037 has been amended to incorporate the following changes to the protocol:

Section 4.2.3: Implementation of changes to eligibility criteria as recommended by the US Food and Drug Administration (FDA).

The FDA strongly recommended that tuberculosis testing be considered at screening. The eligibility criteria of the protocol have therefore been amended to mandate testing for tuberculosis and exclude patients with positive results.

As per FDA feedback the eligibility criteria have also been amended to exclude women who are breast-feeding or planning to nurse.

The eligibility criteria have been amended to explicitly exclude patients who are using strong inhibitors of CYP3A4 and P-glycoprotein (P-gp), as well as compounds inducing CYP3A4. These medications were already specified as prohibited therapies. For consistency with changes to Section 4.5.2, patients using sensitive substrates of CYP3A4 with a narrow therapeutic window are also explicitly excluded.

Section 4.5.2: Update on the use of prohibited therapy as recommended by US FDA

Explicit prohibition of the use of sensitive substrates of CYP3A4 with a narrow therapeutic index has been added to existing cautionary statements about the concomitant use of CYP3A4 substrates.

Section 4.6.1.4: Addition of bicarbonate to laboratory test panel

The European League against Rheumatism (EULAR) Sjögren's Syndrome Disease Activity Index (ESSDAI) assessment includes evaluation of renal acidosis. Measurement of bicarbonate is important for diagnosis of renal acidosis and has consequently been added to the laboratory test panel.

Section 4.6.1.6: Addition of IgM to antibody titre measurements

The ESSDAI assessment includes, among others, evaluation of hypergammaglobulinemia.

Measurement of IgM is required for this assessment and hence total IgM has been added to the list of antibody titres to be measured. As samples for the above were not collected at all visits where ESSDAI was evaluated, additional assessment time points for cryoglobulin, IgG and IgM have been added to the Schedule of Assessment (SoA)

tables and the complement C3/4 assessment has been moved to the blood biochemistry panel.

Section 5.3.5.6: Liver Function Testing (LFTs)

A section on interpretation and reporting of abnormal liver function tests has been inserted to provide more specific guidance for investigators.

Section 6: Statistical Considerations and Analysis Plan

The alpha level for any hypothesis testing has been clarified as 2-sided at the 0.05 level to align with the use of 95% confidence intervals (CIs). Additionally the primary analysis will use a non-responder approach for missing data and an exploratory analysis was pre-specified adjusting for baseline ESSDAI score.

Section 6.7: Pharmacodynamic Analyses

This section has been modified to clarify that pharmacodynamic data will primarily be summarized and listed according to treatment actually received. Limited exploratory analyses employing methods used for efficacy endpoints on the mITT population may be carried out, but will be detailed in the statistical analysis plan.

SUMMARY OF CHANGES

PROTOCOL SYNOPSIS

The protocol synopsis has been updated to reflect the changes to the protocol, where applicable.

SECTION 3.2.6: Rationale for Pharmacodynamic Assessments

Elevated circulating titers of a range of auto-antibodies are reported in patients with primary Sjögren's syndrome (Tzioufas et al 2012), and as no single type is considered diagnostic, prognostic or predictive a panel including a representative cross-section will be measured, as well as total IgG*, IgM* and cryoglobulins (Quartuccio et al 2014).

SECTION 4.2.3: Exclusion Criteria

The exclusion criteria have been updated as follows:

1. A positive test result for hepatitis B (HBV), hepatitis C (HCV), or human immunodeficiency virus (HIV), ~~a history of~~*or* tuberculosis, or any other active viral, fungal, yeast or bacterial infection at the screening visit.

   ....

14. Women who are lactating.

15. Use of other prohibited medication (moderate or potent inhibitors of CYP3A4; strong inducers of CYP3A4; strong inhibitors of the transporter P-glycoprotein [P-gp]; sensitive substrates of CYP3A4 with a narrow therapeutic index).

SECTION 4.5.2: Prohibited Therapy

Because RO5459072 is a substrate of CYP3A4 and P-gp, other prohibited medications are:

- Moderate or potent inhibitors of CYP3A4 (e.g., itraconazole, erythromycin, nefazodone).
- Strong inducers of CYP3A4 (e.g., rifampicin, carbamazepine).
- *Sensitive substrates of CYP3A4 with a narrow therapeutic index (e.g., alfentanil, colchicine, ethosuxamide, fentanyl, and pimozide).*
- Strong inhibitors of the transporter P-glycoprotein (P-gp) (e.g., quinidine, clarithromycin).

A full list of prohibited concomitant medications is provided in Appendix 4.

SECTION 4.6.1.4: Laboratory Assessments

- ***Blood***~~Serum~~ **chemistry:** Albumin, alkaline phosphatase (ALP), alanine aminotransferase (ALT), aspartate aminotransferase (AST), *bicarbonate,* C-reactive protein (CRP), calcium, chloride, creatinine phosphokinase (CPK), gamma-glutamyl transferase (GGT), glucose, phosphate, potassium, creatinine, sodium, total bilirubin, total protein, troponin, urea*, complement (C3 and C4),*
- **Coagulation:** activated partial thromboplastin time (aPTT), prothrombin time (PT)
- **Lipids:** High-density lipoprotein-cholesterol (HDL-C), low-density lipoprotein cholesterol (LDL-C), triglycerides, total cholesterol.
- **Urinalysis** A midstream urine specimen will be collected for dipstick analysis of protein, blood, glucose, and pH.
- At Screening only: HIV (specific tests HIV-1 antibody, HIV-1/2 antibody, HIV-2 antibody), hepatitis B surface antigen (HBsAg), total hepatitis B core antibody (HBcAb), hepatitis C virus (HCV) antibody*, tuberculosis testing*.

SECTION 4.6.1.6: Pharmacodynamic Assessments

Auto-antibodies (anti-SSA/SSB)

Serum will be collected for the determination of anti-SSA/SSB autoantibodies as indicated in the SoA (Appendix 1). These measurements will include anti-SSA, anti-SSB, ANA, and rheumatoid factor, as well as total IgG*, IgM* and cryoglobulins.

….

Soluble Biomarker Assessments

Plasma samples - Samples for measurement of biomarkers connected to Sjögren’s syndrome and extracellular matrix degradation will be collected at specified visits as indicated in the SoA (Appendix 1). Biomarkers to be measured will include~~ complement (C3 and C4),~~ β2-microglobulin, cytokines (including, but not limited to, BAFF, IL-4, IL-6 and IL-10), beta-hydroxycholesterol and desmosine.

SECTION 5.3.5.6: Abnormal Liver Function Tests

The finding of an elevated ALT or AST (> 3 × ULN) in combination with either an elevated total bilirubin (> 2 × ULN) or clinical jaundice in the absence of cholestasis or other causes of hyperbilirubinemia is considered to be an indicator of severe liver injury. Therefore, investigators must report as an adverse event the occurrence of either of the following:

- *Treatment-emergent ALT or AST > 3 × ULN in combination with total bilirubin > 2 × ULN*
- *Treatment-emergent ALT or AST > 3 × ULN in combination with clinical jaundice*

The most appropriate diagnosis or (if a diagnosis cannot be established) the abnormal laboratory values should be recorded on the Adverse Event eCRF (see Section 5.3.5.1) and reported to the Sponsor immediately (i.e., no more than 24 hours after learning of the event), either as a serious adverse event or a non-serious adverse event of special interest (see Section 5.4.2).

SECTION 6.1: Determination of Sample Size

A total of 70 patients will be randomized in a 1:1 ratio to RO5459072 or placebo (35 patients per treatment group). ~~Assuming a maximum drop-out rate of 15%, it is anticipated that enrollment of 70 patients would ensure at least 60 patients complete the study.~~

Seventy~~Sixty~~ patients (3*5*~~0~~ per treatment group) will provide *power in the range of* at least 58 *% to* ~~at least~~ 80 % ~~power~~ to detect a ~~33%~~ difference in ESSDAI response rates between treatment groups *in the range of 25 % to 33 %* (two-sided Chi-square test assuming alpha of 0.*05*~~15~~ and placebo rate ≤ 25%). *This sample size was determined by practical considerations.* S*eventy*~~ixty~~ patients are also sufficient to provide *approximately* ~~8~~*70*~~0~~% power to detect a difference of 3 points between treatment arms in change from baseline in ESSDAI score (t-test assuming two-sided alpha 0.*0*~~1~~5 and standard deviation of 5.0).

Approximately 30 patients will be enrolled into the optional sub-study. It is anticipated that enrollment of a minimum of 30 patients should ensure at least 20 patients (10 per treatment group) complete the study and have biopsy samples taken at both baseline and Week 12*, assuming a maximum drop-out rate of 15%*. ~~This sample size was determined by practical consideration.~~

SECTION 6.3.2: Pharmacodynamic Analysis Population

All patients who have received at least one dose of the study medication and who have pharmacodynamic data available will be included in pharmacodynamic analyses. For these analyses, patients will primarily be grouped according to the treatment actually received; unless otherwise specified in the statistical analysis plan.

~~Analyses of pharmacodynamic data will employ similar methods as used for continuous efficacy endpoints, i.e., analyses will employ the mITT population.~~

SECTION 6.6.1: Primary Efficacy Endpoint

The primary endpoint is the proportion of patients who have ≥ 3 point reduction from baseline in ESSDAI score after 12 weeks of treatment. The proportion of ~~subjects~~ *patients* who have ≥ 3 point reduction from baseline in ESSDAI score after 12 weeks of treatment will be compared between the two treatment arms using *a* Pearson Chi-square test *(two sided p-values, alpha 0.05). The difference in proportions* ~~Two-sided p-values will be reported. Absolute reduction in risk~~ and corresponding 95% confidence interval

(CI) will be provided. *Patients with missing data at Week 12 will be treated as non--responders in the analysis*~~Missing data will be imputed by last observation carried forward (LOCF) method~~.

Sensitivity analyses will be conducted if deemed necessary, including sensitivity analyses ~~for imputational method~~*for handling* ~~for~~ missing data (e.g.*,* ~~.,~~ ~~completers~~*completer's* analysis or *last observation carried forward (LOCF) method*~~imputing non-responders~~ for missing observations).

In addition; the proportion of patient~~subject~~s who have $\geq$ *3 point reduction from baseline in ESSDAI score after 12 weeks of treatment will be analyzed using a logistic regression model, adjusting for baseline ESSDAI score.*

SECTION 6.6.2: Secondary Efficacy Endpoint

The denominator degrees of freedom will be estimated using *the Kenward-Roger.* ~~Satterthwaite's~~ approximation.

SECTION 6.7: PHARMACODYNAMIC ANALYSES

All pharmacodynamic parameters will be presented by listings and descriptive summary statistics separately by treatment group.

Pharmacodynamic variables at each assessment time-point along with corresponding changes from baseline will be listed by individual and summarized using descriptive summary statistics. Descriptive summary statistics will include the calculation of arithmetic means, standard deviation, coefficient variation, median, and range, as appropriate. Graphical plots of individual and mean absolute and change from baseline versus time profiles will also be produced for each treatment.

~~Analyses of pharmacodynamic data will employ similar methods as used for continuous efficacy endpoints, i.e., change from baseline at on-treatment visits will be compared between the treatment groups using a MMRM approach in the mITT population.~~

APPENDIX 1: Schedule of Assessments

The schedule of assessments has been revised to reflect the changes to the protocol.

APPENDIX 4: Prohibited Concomitant Medication

MDR1 (P-gp) inhibitors

Amiodarone, azithromycin, carvedilol, clarithromycin, dronedarone, erythromycin, fluvoxamine, indinavir, itraconazole, ketoconazole, lapatinib, propafenone, quercetin,

quinidine, quinine, ranolazine, ritonavir (and ritonavir combinations), telaprevir, verapamil, vorapaxar.

Sensitive substrates of CYP3A4 with a narrow therapeutic index

Alfentanil, colchicine,ethosuxamide, fentanyl, pimozide.

Various sections:

Correction of minor inconsistencies between protocol sections and typographical errors has been done. Additional tuberculosis test at screening has been included. For clarity, tear and salivary flow assessments have been indicated in the SoA table (in line with salivary and tear sample collection time points), and additional biomarker sample collection time points (for the ESSDAI score) have been entered for the early termination/ follow-up visit in the SoA table. Furthermore, Figure 3 Study Design, was adjusted for improved clarity regarding the safety and follow-up visit.

MASTER INFORMED CONSENT FORM

The master Informed Consent Form been revised to reflect the changes to the protocol.
