# Supplementary material for: A randomized, double-blind, placebo-controlled, parallel group study on the effects of a cathepsin S inhibitor in primary Sjögren’s syndrome
Source: Rheumatology (Oxford). 2023 Mar 2;62(11):3644–53. doi: 10.1093/rheumatology/kead092 (PMC10629789; doi:10.1093/rheumatology/kead092)
Supplement: kead092_Supplementary_Data [file kead092_supplementary_data.zip › kead092_Supplementary_Data/rhe-22-2329-File004.docx]

Supplementary Figure S1. Breakdown of ESSDAI scores at baseline (ITT population)

Study arm

(PBO, placebo; 100 mg, RO5459072 100 mg BID)

Study arm

(PBO, placebo; 100 mg, RO5459072 100 mg BID)

CNS, central nervous system; ESSDAI, EULAR Sjögren's Syndrome Disease Activity Index; ITT, intent-to-treat; PNS, peripheral nervous system.

Supplementary Figure S2. Mean plot of ESSDAI scores at baseline (ITT population)

Study arm

(PBO, placebo; 100 mg, RO5459072 100 mg BID)

CNS, central nervous system; ESSDAI, EULAR Sjögren's Syndrome Disease Activity Index; ITT, intent-to-treat; PNS, peripheral nervous system.

Supplementary Figure S3. Adjusted Mean Plot of Change from Baseline in ESSDAI (mITT Population).


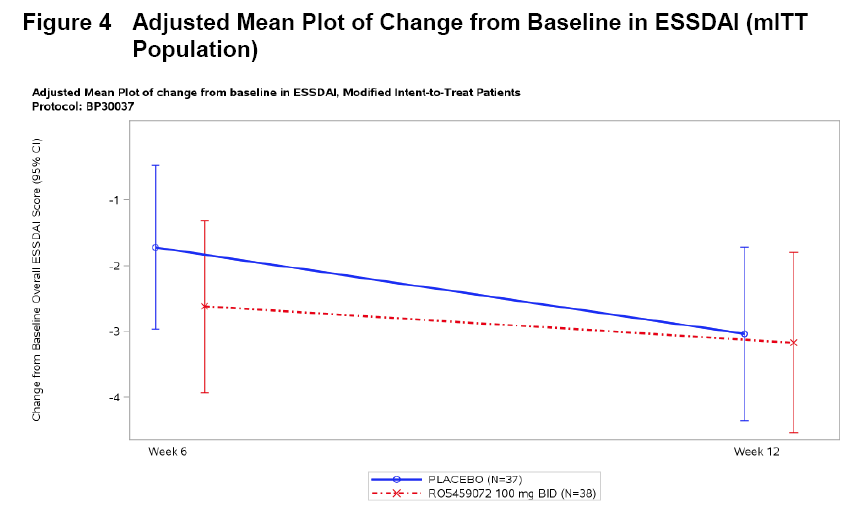


Negative change represents an improvement.

Supplementary Figure S4. Adjusted Mean Plot of Change from Baseline in ESSPRI (mITT Population).


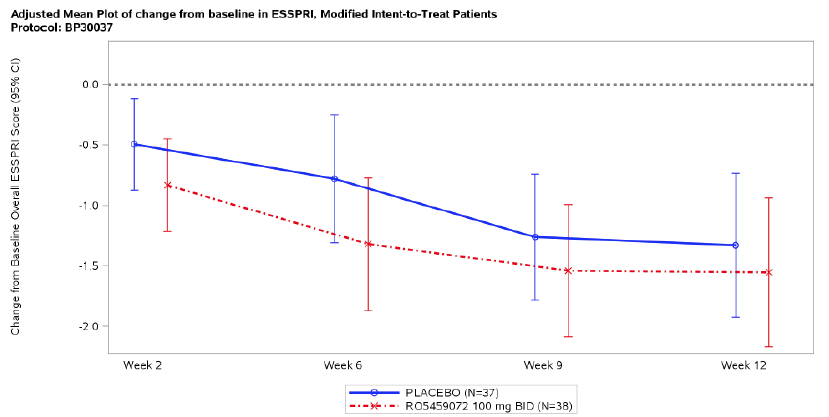


Negative change represents an improvement.
